# Bi-directional highways and super-seeder tissues underpin parasite dissemination in experimental *Leishmania donovani* infection

**DOI:** 10.1038/s41467-026-77072-4

**Published:** 2026-08-26

**Authors:** Ciara Loughrey, Juliana B. T. Carnielli, Shoumit Dey, Helen Ashwin, Sally James, Lesley Gilbert, Samantha Donninger, Alastair Droop, Grant Calder, Karen Hogg, Jon Pitchford, Jeremy C. Mottram, Paul M. Kaye

**Affiliations:** 1 York Biomedical Research Institute, Hull York Medical School, University of York, York, UK; 2 York Biomedical Research Institute, Department of Biology, University of York, York, UK; 3 Biosciences Technology Facility, Department of Biology, University of York, York, UK; 4 Department of Mathematics, University of York, York, UK

**Keywords:** Parasite host response, Microbial ecology

## Abstract

Visceral leishmaniasis (VL) is a life-threatening parasitic disease caused by *Leishmania donovani* and *L. infantum*. Although VL is characterised by organ-specific immunopathology and parasitism of the spleen, liver and bone marrow, other tissues may harbour parasites without overt pathology. The mechanisms governing patterns of within-host dissemination and tissue tropism are largely unknown. Here, we use a barcoded library of isogenic *L. donovani* and an ecological analysis framework, to evaluate parasite population diversity across tissues and to map parasite dissemination in a murine infection model. We reveal an unexpected high degree of inter-connectivity between parasite populations demonstrating: i) continual bi-directional dissemination between viscera and skin; ii) the existence of super-seeder sites that act as hubs fuelling systemic spread; and iii) rerouting of dominant dissemination routes or highways following immune perturbation. These findings change our understanding of *L. donovani* pathogenesis, providing critical insights into infection dynamics, with implications for relapse, treatment failure and transmission.

## Abstract (web summary)

Ecological analysis of murine *Leishmania donovani* infection reveals complex parasite within-host dynamics, with super-seeder sites fuelling dissemination between skin and viscera, and with key connections malleable after further immune perturbation.

## Introduction

The leishmaniases are vector-borne neglected tropical diseases, caused by intracellular protozoan parasites of the genus *Leishmania*. In mammalian hosts, these parasites primarily reside within myeloid cells, notably macrophages, monocytes, dendritic cells and neutrophils^1–7^, although evidence indicates they can also infect other cell types including haematopoietic stem cells (HSCs) and non-phagocytic cells such as fibroblasts^8–11^.

Visceral leishmaniasis (VL), caused by *L. donovani* and *L. infantum*, is a life-threatening systemic form of leishmaniasis and is endemic in ~60 countries. Clinically, VL is characterised by gross pathology and high parasite loads in the liver, spleen and bone marrow^12,13^. Studies in experimental models^14–18^, reservoir species^19^ and patients, especially those immunocompromised by HIV co-infection^20–22^, indicate that parasites can disseminate widely even in the absence of overt local tissue pathology. The skin is particularly relevant, acting as an alternate route to blood for transmission^17,23^ and as the target tissue for post kala-azar dermal leishmaniasis (PKDL)^24–26^, a post-treatment sequela observed in 50–60% of VL patients in Sudan and up to 10% in India^26^. Beyond PKDL, the concept of persistent parasites residing in sanctuary tissues has also been suggested as a mechanism underpinning VL relapse and transmission, even after drug cure^9,27–29^.

Despite its clinical importance, little is known about within-host pathways of parasite dissemination into diverse tissues. Ecological and population dynamics principles offer a powerful framework for in vivo investigations to address the dynamics of microbial dissemination and colonisation patterns^30^. The adaptation of macro-ecological concepts such as alpha and beta diversity, bottlenecks and assembly processes can enable elucidation of complex interactions between pathogens and hosts, and aid the identification of mechanisms involved in shaping pathogenesis and spread^31–34^.

Recent advances in CRISPR genome editing have enabled genetic barcoding and the creation of isogenic libraries of pathogens with neutral, distinguishable alleles. This technique allows for detailed tracking of pathogen population dynamics in experimental models^31^, including through imaging^35–37^, lineage tracing^38^, experimental evolution^39^ and competition-based screening^40–45^. Sequence-tag based analysis of microbial population dynamics (STAMP) was initially developed by Abel et al.^46^ and was described in the context of bottleneck quantification in a *Vibrio cholerae* rabbit infection model; the analytical approach involved the adaptation of previous work by Cavalli-Sforza and Edwards^47^ and Krimbas and Tsakas^48^ to analyse colonisation bottlenecks and relationships between pathogen populations in different tissue niches. The approach has since been used with other bacterial pathogens including *Pseudomonas aeruginosa*^49,50^, *Citrobacter rodentium*^51^, *Listeria monocytogenes*^52–54^*, Salmonella enterica*^55^*, Klebsiella pneumoniae*^56^*, Yersinia pseudotuberculosis*^57^ and *Escherichia coli*^33^, but to date has only been applied to one eukaryotic pathogen, *Toxoplasma gondii*^58^.

Here we leveraged this approach by generating an isogenic barcoded library of *L. donovani* to map systemic dissemination in a C57BL/6 J murine experimental model of VL. We developed a network-based analytical approach to infer parasite population spread between tissues. Our findings reveal an unexpected degree of bi-directional communication between the skin and visceral organs and identify specific super-seeder tissues which act as major sources for widespread dissemination within a host. Furthermore, using a re-infection model, we demonstrate that dissemination routes can be significantly perturbed by secondary exposure. These data provide fundamental insights into *L. donovani* pathogenesis, offering potential explanations for observed treatment failure and relapse in human disease.

## Results

### Generation of an isogenic, barcoded L. donovani library

**Fig. 1:**
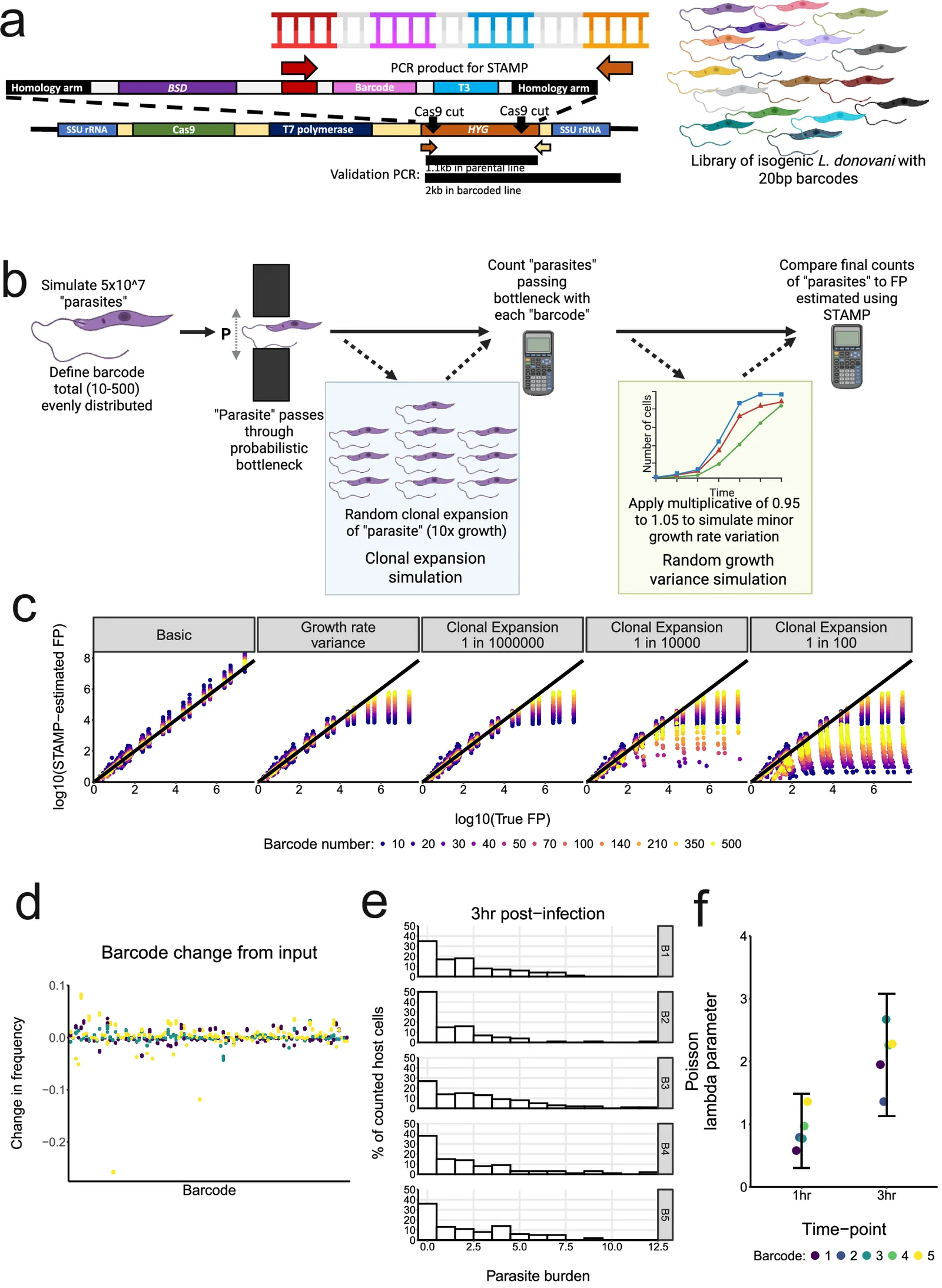
Use of CRISPR-cas9 genome editing to create a library of neutrally barcoded *L. donovani* promastigotes. **a** Barcode insertion strategy for *L. donovani*. 20 bp barcodes were inserted alongside a blasticidin resistance gene (BSD), replacing the hygromycin resistance gene within the T7 cas9 cassette of the parental line. Coloured promastigote parasites indicate different barcodes. A size-based PCR was used to validate integration into the desired location. Primer binding sites shown in orange and beige. Primers for amplification of barcodes for Illumina sequencing are shown in red and orange. Created in BioRender. Loughrey, C. (2026) (https://BioRender.com/8b74bbl). **b** Overview of the simulation strategy for assessing optimal library size. Simulations tested Founder Population (FP) calculation accuracy with: an even replication rate post-bottleneck; a 5% random variance in overall growth; and 5% random variance plus random clonal expansion of 1 in 1000000 (rare), 1 in 10000 (medium) and 1 in 100 (common) individuals, with a clonal expansion growth rate simulated as 10 times greater than that of other individuals. Created in BioRender. Loughrey, C. (2026) https://BioRender.com/pa0poy8**c** STAMP-calculated estimates of FP against the true counted FP from the simulations in b. Colours indicate the number of barcodes in the library tested as shown in the key. Dark grey line indicates perfect estimation accuracy (true FP is equal to STAMP-estimated FP). **d** Frequency change from input frequency for each barcode in barcoded *L. donovani* library pools cultured as promastigotes for 7 days (*n* = 5 for each input pool) minus total counts for each in the input culture pool. Colour indicates experiment (three independent replicate experiments). For each barcode, each point indicates one of five replicates within an experiment. **e** Histograms of in vitro parasite infection burdens after a 3-hr incubation of promastigote parasites with murine bone marrow derived macrophages for five individual barcoded parasite lines, termed B1-5. **f** Poisson-fitted lambda values for the distribution of parasites per macrophage for each barcoded line infection for 1 or 3 hrs (as described in **e**). Colour indicates individual barcoded parasite line in f. Error bars indicate mean +/− 2x standard deviation for lambda values for the five independent parasite lines described in e at each time-point. Source data are provided as a Source Data file.

To track parasite dissemination in vivo, we engineered an isogenic barcoded library of *L. donovani* promastigotes using CRISPR-Cas9 genome editing. We inserted 20 bp barcodes into a neutral exogenous locus (Fig. 1a). To determine the necessary library size for accurate Founder Population (FP) estimation using STAMP, we combined prior knowledge of the limitations of macro-ecological analysis (predominantly designed for relatively small numbers of “species”)^59^ and previous similar studies^52–54,58^ with a MATLAB-based simulation (see “Methods” for details). These simulations modelled various library sizes (10–500 barcodes) and scenarios of even and varied growth of individual parasites (Fig. 1b). We modelled the impact of random clonal expansion by selecting either 1 in 100 (common), 1 in 10000 (medium) or 1 in 1000000 (rare) individual parasites post-bottleneck to replicate at an enhanced rate of ten times the standard. Except in cases of common clonal expansion (which we anticipate is unlikely to occur in amastigote *Leishmania*), we observed that the accuracy of FP estimates was consistently high for all library sizes up to estimated FPs of 5000 (Fig. 1c and Supplementary Data 1 and 2; Kendall correlation for FP values below 5000: all tau values above 0.83, *p* < 0.0001 for simulations other than common clonal expansion). Whilst FPs above this value cannot be discriminated effectively without a larger library, FPs below this point can be quantified using libraries within the limits tested here. We generated a library with 102 unique barcodes, with successful integration validated by PCR, and for a selected few, Sanger sequencing (Fig. 1a and Supplementary Fig. 1a).

We confirmed that the inserted barcodes did not induce changes in parasite fitness. Culturing the full library for 7 days showed no evidence of any individual barcodes exhibiting a trend to increase or decrease beyond what would be expected to occur at random (Fig. 1d). Furthermore, in vitro infectivity assays using murine bone marrow derived macrophages (BMDMs) demonstrated that all lines assessed (*n* = 5) had similar distributions of parasites per macrophage (Fig. 1e and Fig. 1f and Supplementary Fig. 1b; lambda parameters within 2 standard deviations at 1- and 3 h post-infection [p.i]), confirming the neutrality of barcoded lines for in vivo studies.

### Tissue parasite population diversity reflects stage of infection

**Fig. 2:**
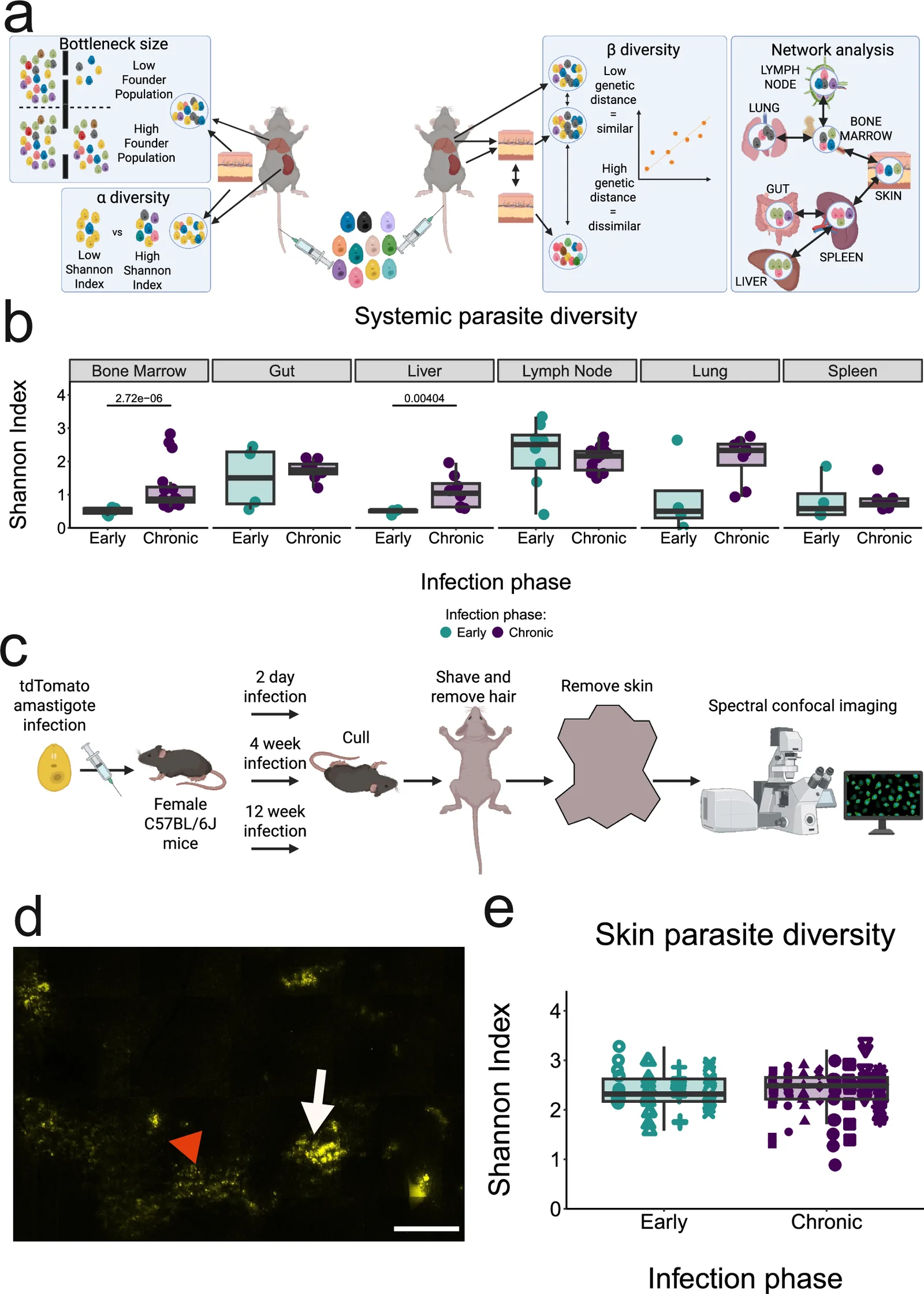
Parasite population structure varies both spatially and temporally. **a** Schematic illustrating macro-ecology and graph theory concepts applied in this study. Created in BioRender. Loughrey, C. (2026) (https://BioRender.com/5ooxaha). **b** Parasite diversity (Shannon Index [SI]) in systemic tissues of female C57BL/6 J mice infected with barcoded amastigotes for 4 weeks (early; *n* = 4; turquoise) or 10–12 weeks (chronic; *n* = 8; purple). For each mouse, two lymph nodes and two bone marrow samples were analysed and as such *n* number is doubled for visualisation and statistical analysis. **c** Schematic of protocol for analysis of skin parasite distribution. *n* = 4 each for 2 day and 4 week infections. *n* = 3 for 12 week infections. Created in BioRender. Loughrey, C. (2026) (https://BioRender.com/dnqj3nd). **d** Representative image (after stitching of panels) of a section of late infected skin. The full size confocal image and images of the two other 12 week infected mice (total *n* = 3) are shown in Supplementary Fig. 2. Parasites are identified by high (white arrows) and low (red arrowheads) tdTomato signal (yellow). Scale bar represents 2000 μm. **e** Parasite diversity (SI) in skin biopsies (12 per mouse) for the mice described in panel b (early; *n* = 4 and chronic; *n* = 8); all 12 data points per mouse are shown and used for statistical comparison. Shapes indicate individual mice. All boxplots show the median, 25th and 75th quartiles, with whiskers representing all data-points within 1.5x IQR of the hinges; points above or below the whiskers represent outlying data values. *p* values from a two-tailed Mann-Whitney-Wilcoxon test for within tissue time-point comparisons. Source data are provided as a Source Data file.

To examine temporal and spatial changes in parasite populations over the course of infection, we injected amastigotes of barcoded *L. donovani* intravenously into female C57BL/6 J mice (Fig. 2a), as this is an established model for generating reproducible systemic infections^60^. Our broad aim was to apply diversity measures both within and between tissue samples within an individual animal, and to use this information to perform network analysis on the ecosystem of parasites within the host (Fig. 2a).

We first used the concept of alpha (within site) diversity^61–63^ to analyse parasite populations at 4 weeks (early) and 10–12 weeks (chronic) p.i (Fig. 2b). Our analysis revealed a significant increase in parasite diversity (quantified using the Shannon Index, which measures both richness and evenness of the population^61,63^) in bone marrow (*p* < 0.0001) and liver (*p* = 0.00404) (Fig. 2b and Supplementary Data 3). Early post-infection, spleen, liver and bone marrow parasite populations showed a relatively consistent degree of diversity between mice, whereas both median diversity and inter-mouse variation was greater in the lymph node and gut. This may reflect different kinetics or routes for colonisation in these tissues. These findings are important as they suggest that tissue colonisation is not governed by a single bottleneck event but rather reflects a continuous process of recruitment and colonisation throughout the course of infection.

We have previously shown that parasites are located in patches in the skin of immuno-compromised *B6.Rag2*^*-/-*^ mice^17,64^. We therefore adopted the same analytical approach to visualise skin parasites during early and chronic infection in immuno-competent C57BL/6 J mice, using a tdTomato-expressing *L. donovani* line (Fig. 2c). Confocal imaging detected parasite patches only during chronic infection (defined here as 12 weeks p.i) and not at the two earlier time-points assessed (4 weeks and 2 days p.i) (Fig. 2d, Supplementary Figs. 2, 3 and 4). Of note, heterogeneity in the tdTomato signal was observed. This may reflect differences in patch parasite burden and/or the presence of quiescent parasites with reduced metabolic activity^8,28,29^.

To examine parasite population diversity in the skin, we sequenced 12 spatially adjacent biopsies per mouse taken from randomly selected locations on the flank. The average parasite diversity in these biopsies was similar between early and chronic infection. However, the diversity of biopsies derived from a single mouse was highly heterogeneous, with individual mouse being the best predictor of parasite diversity in skin biopsies, rather than time post-infection (Fig. 2e; Compound Poisson Generalised Linear Mixed Model [GLMM], see Supplementary Note). Overall, the diversity in skin, lymph nodes, lung and gut was higher and more variable than in the bone marrow, spleen and liver (Fig. 2b), supporting a model of stochastic and continuous seeding of the skin, gut and lymph nodes from visceral tissues^17,18,64^.

### Skin patches vary in their similarity to parasite populations in other tissues

**Fig. 3:**
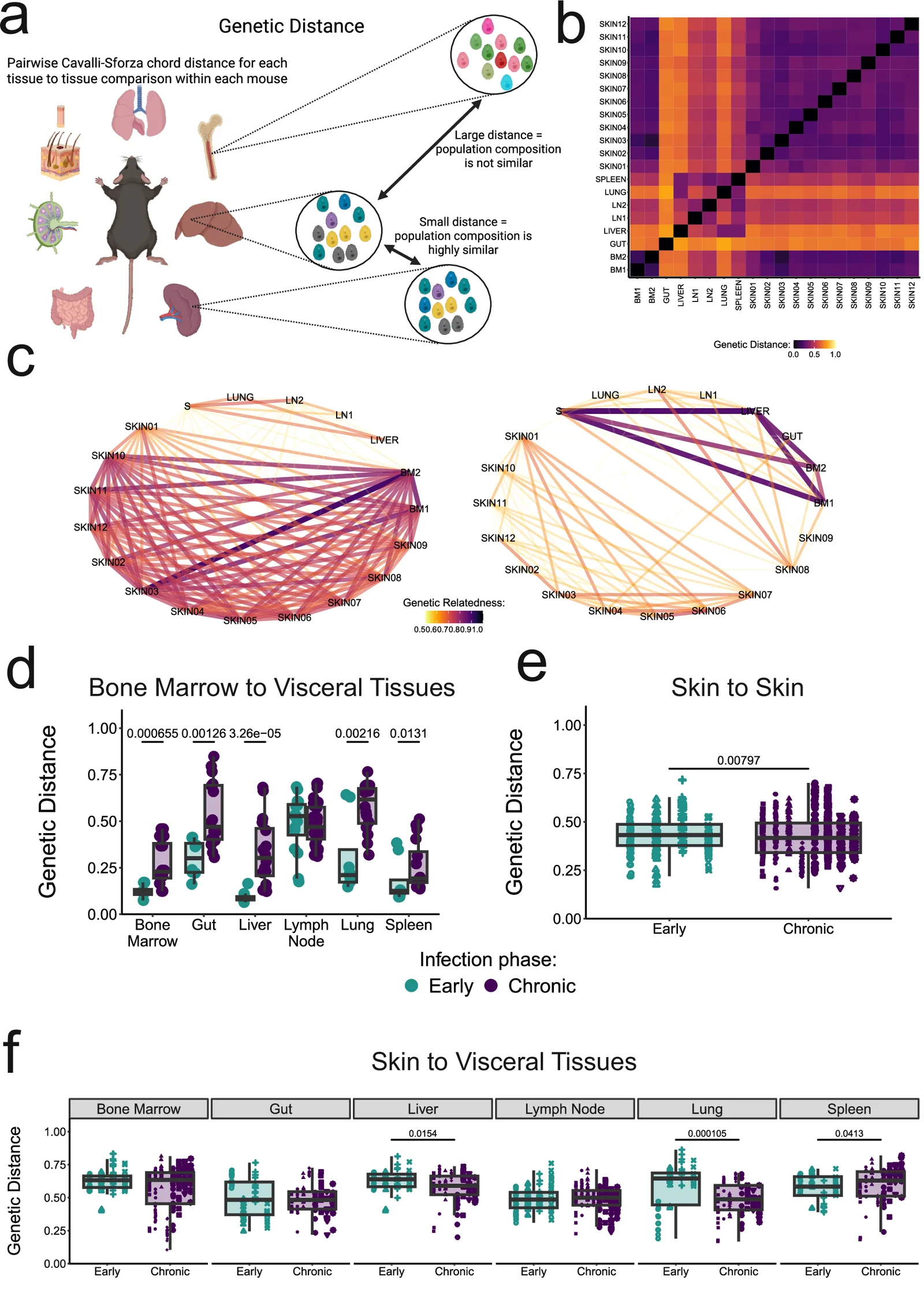
Genetic distance analysis reveals heterogenous skin landscape, with increasing connectedness over the course of infection. **a** Schematic of genetic distance (GD) concept for pairwise comparison of tissue parasite populations in individual mice. Created in BioRender. Loughrey, C. (2026) (https://BioRender.com/h75jkxg). **b** Example heatmap for a single mouse at 12 weeks post-infection. **c** Network representations of GD (edges) between parasite populations in each tissue (vertices). Networks for two exemplar mice (Mouse 21 and Mouse 23) at 12 weeks post-infection, with all GD above 0.5 removed. Line colour indicates genetic relatedness (1-GD) as shown in the legends for each network. Thicker, more opaque connecting lines indicate a higher relatedness (lower GD). **d** GD for bone marrow and all other systemic tissues in female mice with early (turquoise; *n* = 4) or chronic (purple; *n* = 8) infection. For each mouse, two lymph nodes and two bone marrow samples were analysed, meaning n number is doubled for visualisation and statistical analysis. **e** GD for pairwise comparisons of each skin biopsy with all other skin biopsies within each individual mouse (*n* = 12 biopsies per mouse). **f** GD for pairwise comparisons of each skin biopsy with visceral tissues within each individual mouse (*n* = 12 biopsies per mouse). Shape indicates individual mice. Colour legend applies to **d**–**f**. In **b**, **c** BM, bone marrow; S, spleen; and LN, lymph nodes. All boxplots show the median, 25th and 75th quartiles, with whiskers representing all data-points within 1.5x IQR of the hinges; points above or below the whiskers represent outlying data values. *p* values from a two-tailed Mann-Whitney-Wilcoxon test for within tissue time-point comparisons. GD statistical testing: Bone Marrow to Bone Marrow: *p* = 0.000655; Bone Marrow to Gut: *p* = 0.00126; Bone Marrow to Liver: *p* < 0.0001; Bone Marrow to Lung: *p* = 0.00216; Bone Marrow to Spleen: *p* = 0.0131; Skin to Skin: *p* = 0.00797; Skin to Liver: *p* = 0.0154; Skin to Lung: *p* = 0.000105; Skin to Spleen: *p* = 0.0413. Source data are provided as a Source Data file.

We next used the ecological concept of beta (between site) diversity, calculated via genetic distance [GD] using the Cavalli-Sforza chord distance^47^) to quantify the relatedness between parasite populations in different tissues (Fig. 3a). A heatmap representation confirmed extensive tissue inter-connectivity, though the specific pattern varied widely between individual mice. For example, in the representative mouse shown in Fig. 3b, the liver parasite population is more highly related to that of the lung and spleen than to other tissues. Parasites in skin biopsy 03 are most similar to those in bone marrow and to a lesser extent other skin biopsies.

To visualise the parasite ecosystem in individual mice, we used 1-GD (i.e. genetic relatedness) as a connection strength measure for a graphical network (Fig. 3c and Supplementary Fig. 5). We found that while visceral tissues were consistently linked during early infection (Supplementary Fig. 5), the linkage between skin and viscera became highly heterogeneous during chronic infection (Fig. 3c and Supplementary Fig. 5). We further noted that bone marrow became more genetically distant from all other visceral tissues, aside from lymph nodes, over time (Fig. 3d). Except for the gut becoming more distant from spleen and liver, no other visceral pairs showed significant changes in relatedness (Supplementary Data 4). We also found that the average relatedness of the parasites in each skin biopsy was greater (reduced GD) at chronic infection compared to early infection, but again high intra-mouse variation made individual a better predictor of inter-skin relatedness (Fig. 3e, Supplementary Data 4 and Supplementary Note).

A distinct clustering effect was observed when comparing skin biopsies to visceral organs; some mice had skin patches highly related to one key visceral tissue, whilst others showed low relatedness (Fig. 3f). Some of these visceral tissue-skin relationships also changed over time when we compared individual biopsy-viscera GDs. We also noted tissue-specific differences had an interaction effect with time p.i (Supplementary Data 4 and Supplementary Note). This suggests that the relationship between parasites in different visceral tissues and in skin varies over time in a manner that depends on the tissue in question. Furthermore, comparison of GD to the original input pool indicated that a single colonisation event from our original injection pool could not account for the patterns we had observed across tissues in our parasite population diversity and GD analysis (Supplementary Fig. 6), indicating that later tissue-to-tissue dissemination does indeed play a role in parasite tissue colonisation patterns.

### Identification of ‘super-seeder’ sites

**Fig. 4:**
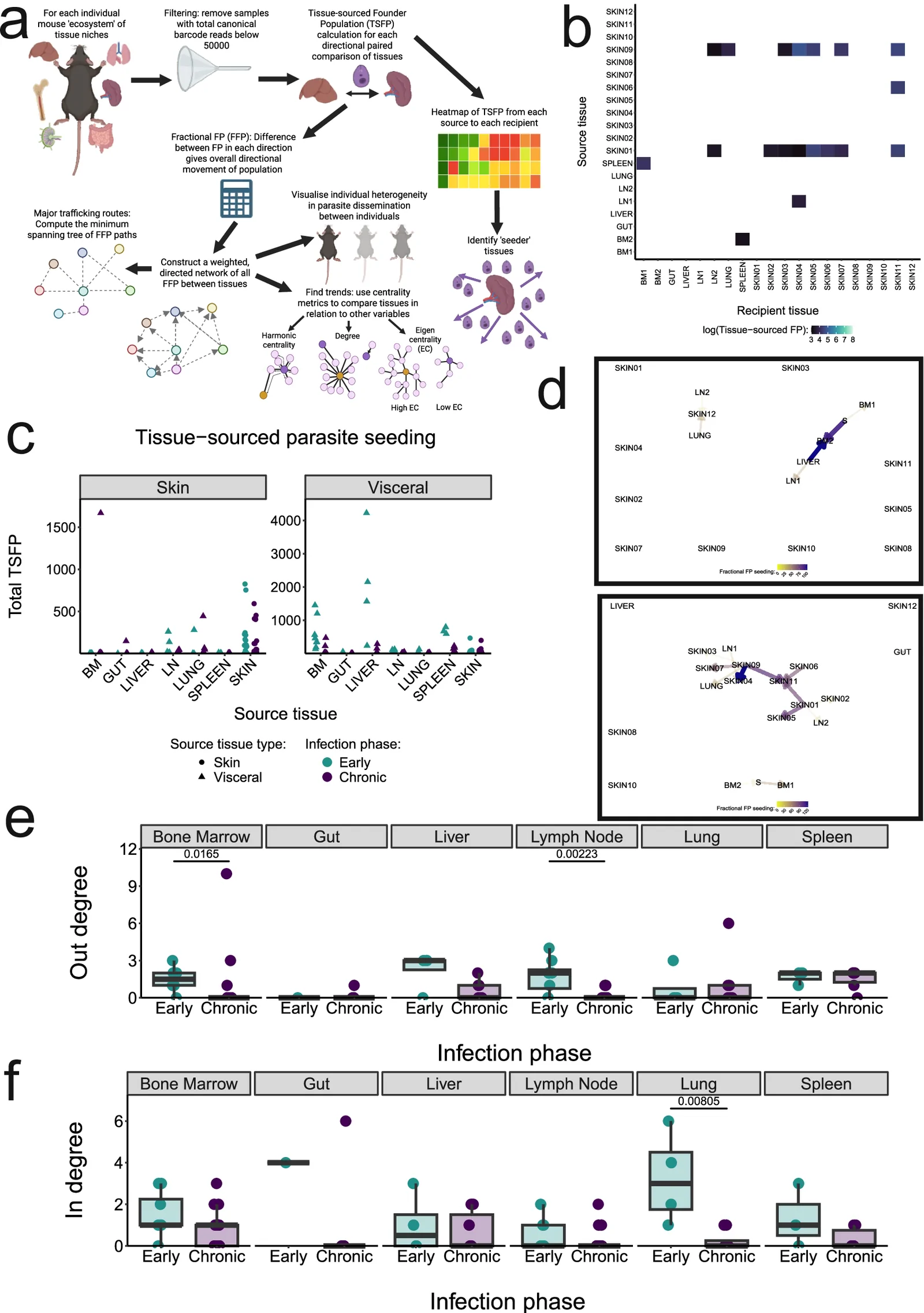
Fractional Founder Population (FFP) analysis reveals super-seeder parasite populations. **a** Schematic illustration of the analytical pipeline to visualise and assess parasite dissemination pathways. Created in BioRender. Loughrey, C. (2026) (https://BioRender.com/wgatt59). **b** Exemplar heatmap (Mouse 24) for Tissue Sourced Founder Population (TSFP) for an individual mouse at 12 weeks post-infection. Scale represents log(TSFP) after thresholding applied (cut-off TSFP = 20) for each directional tissue to tissue relationship. White indicates those TSFP values below threshold or tissues which were excluded in filtering. **c** Total TSFP sourced from each tissue sample to either skin (left) or visceral (right) recipient tissues. Data from early (turquoise; *n* = 4) or chronic (purple; *n* = 8) infection are shown. **d** Minimum spanning tree (MST) network representations of FFP for two exemplar mice (Mouse 22 and Mouse 24) at 12 weeks post-infection. Legends, line colour and thickness indicate FFP. Arrowheads represent direction of FFP. **e,** **f** Out-degree (**e**) and in-degree (**f**) values for each visceral tissue in the full FFP networks constructed for each mouse. Infection phase colour legend applies to **c**, **e** and **f**. In **b**–**d**, BM bone marrow, S spleen, and LN lymph nodes. All boxplots show the median, 25th and 75th quartiles, with whiskers representing all data-points within 1.5x IQR of the hinges; points above or below the whiskers represent outlying data values. All statistics shown are *p* values from a two-tailed Mann-Whitney-Wilcoxon test for within tissue comparisons between time-points. Source data are provided as a Source Data file.

To infer directionality of parasite dispersal events within a host, we developed a new analytical approach (Fig. 4a and Methods). Our metric Tissue-Sourced Founder Population (TSFP) calculates the FP for a tissue with reference to an input population from another tissue and can be used in a pairwise manner to infer routes of parasite trafficking within each mouse. This approach relies on the overall divergences between barcode frequencies to calculate a probable direction and relative size of pathogen movement between tissues. It should be noted that this inferred migration is not definitive, as we cannot directly observe parasite migration.

Heatmap visualisation of TSFP values (Fig. 4b and Supplementary Fig. 7) revealed that many mice exhibited one or two high out-degree super-seeder sites, acting as major parasite sources for multiple other sites. For example, in the mouse shown in Fig. 4b, skin biopsies 01 and 09 act as a source for parasites found in multiple other skin biopsies, as well as lung (biopsy 09) and lymph node (both biopsies). While the liver and bone marrow predominantly acted as sources for parasites in other visceral tissues, both visceral organs and some skin sites could act as super-seeders. Importantly, the skin was found to seed both other skin sites and, critically, visceral tissues (Fig. 4c). In relation to our simulation findings showing a lack of discrimination power above 5000 where growth rate is variable, we noted that all calculated TSFPs were below this level and thus not impacted by this confounder. We observed skin-skin and skin-viscera seeding consistently across both early and chronic mice, predominantly with low FPs for most biopsies, but clear evidence of larger scale seeding events in a small number of cases (Supplementary Data 5 and 6). Viscera-viscera and viscera-skin dissemination were also widely observed, again at variable magnitudes (Supplementary Data 7 and 8).

We then calculated the difference between the TSFP values in each direction for each pair of tissues; we termed this metric Fractional Founder Population (FFP). Using FFP to build directed networks, we visualised the dominant dissemination routes, or highways, of parasite dissemination (Supplementary Fig. 8) via a minimum spanning tree (MST) analysis (Fig. 4d and Supplementary Fig. 9). This revealed heterogeneity in dissemination patterns between individual mice. For example, as shown in Fig. 4d, the parasite highway may extend along a visceral axis, or be predominantly within skin. Using the complete FFP networks (Supplementary Fig. 8), we next calculated for each visceral tissue the out-degree (i.e. the number of outward connections, signifying a tissue’s ability to act as a parasite source) and in-degree (i.e. the number of inward connections, signifying receipt of parasites from elsewhere). Supporting early observations that suggested increasing isolation of the bone marrow (Fig. 3d), this analysis showed that out-degree decreased significantly during chronic infection (*p* = 0.0165; Fig. 4e; Supplementary Data 9 and 10). We also found that the lymph nodes and lung exhibited a reduction in out- and in-degree respectively (*p* = 0.00223 and *p* = 0.00805; Fig. 4e and Fig. 4f; Supplementary Data 9 and 10). Whilst not statistically significant, we noted a trend towards reduced out-degree in the liver, which is likely a reflection of its transition to control of parasite burden in chronic infection.

### Skin parasites may act as a re-seeder of visceral organs

**Fig. 5:**
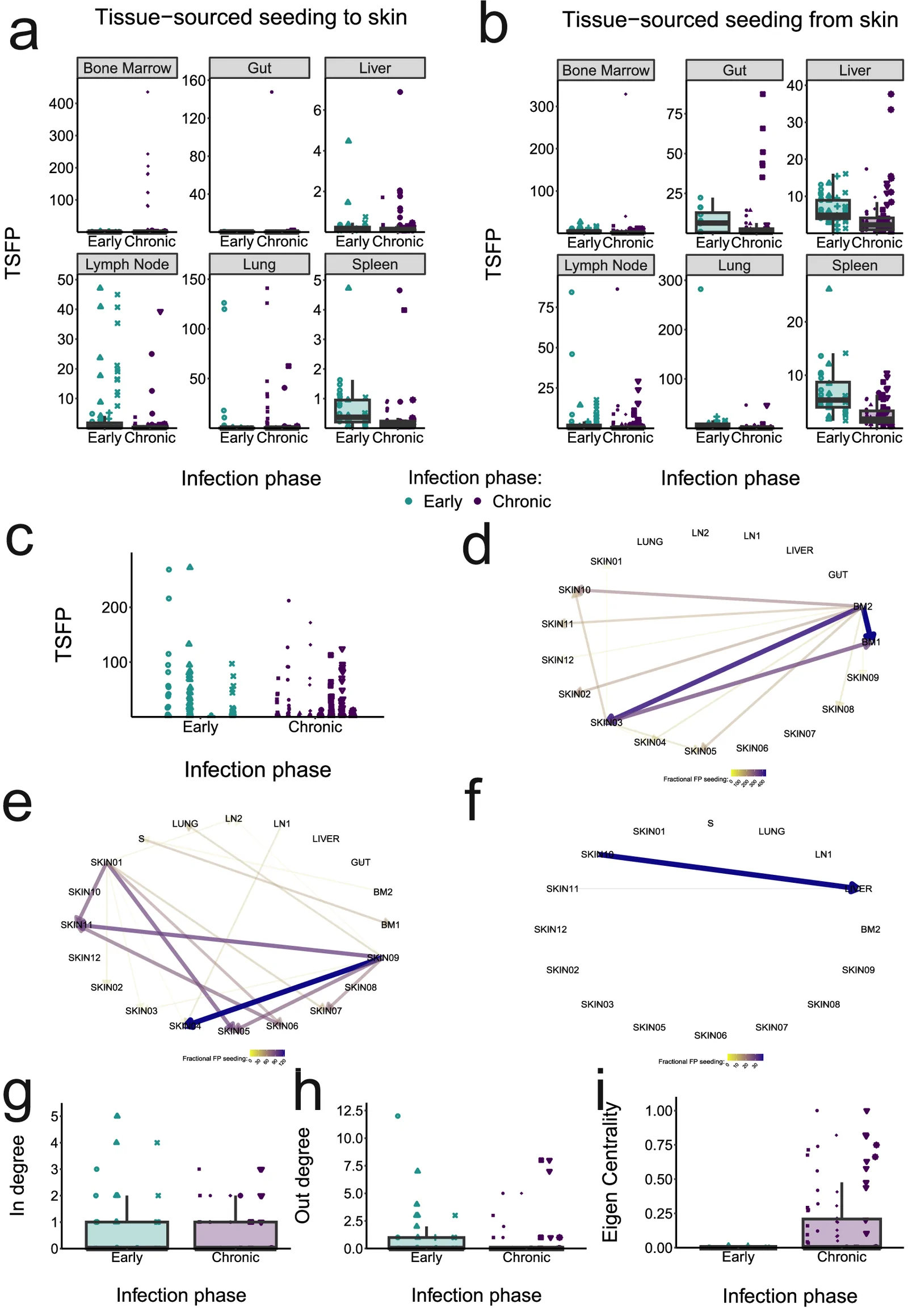
Evidence of skin parasite spread within the skin and to visceral organs. **a**, **b** Tissue Sourced Founder Population (TSFP) from visceral tissues to skin (**a**) and from skin to visceral tissues (**b**) for early (turquoise; *n* = 4) or chronic (purple; *n* = 8) mice. **c** Skin-to-skin seeding, quantified as TSFP, taken from each mouse described in a and b (*n* = 12 biopsies per mouse). **d**–**f** Full networks constructed using FFP as the metric for edge weight, for Mouse 21 (**d**), Mouse 24 (**e**) and Mouse 25 (**f**). Line colour indicates FFP as in legends. Thicker, more opaque lines indicate a higher FFP in the direction indicated by the arrowhead. **g**–**i** In-degree (**g**), out-degree (**h**) and eigen centrality (**i**) for skin sites in the FFP networks. Shape indicates biopsies from each individual mouse within each time-point group. Infection phase colour legend applies to **a**–**c**, **g**–**i**. In **d**–**f** BM bone marrow, S spleen, and LN lymph nodes. All boxplots show the median, 25th and 75th quartiles, with whiskers representing all data-points within 1.5x IQR of the hinges; points above or below the whiskers represent outlying data values. Source data are provided as a Source Data file.

Our prior GD analysis (Fig. 3f and Supplementary Data 4 and Supplementary Note) indicated that skin parasite populations may be interacting with visceral tissues in a dynamic manner. Further TSFP analysis confirmed dynamic, bi-directional communication between the skin and visceral organs (Fig. 5a,b). For viscera to skin seeding, this revealed a characteristic pattern where skin patches were seeded from one or two key visceral tissues, with the source tissue varying between individual mice (Fig. 5a, b). However, comparing total TSFP seeding to the sum of all TSFP to skin biopsies for each mouse indicated no significant change between early and chronic stages for any specific source tissue (Supplementary Data 11). Total skin-to-spleen and skin-to-lung seeding decreased during chronic infection (Fig. 5b and Supplementary Data 12). Skin-to-skin seeding across all biopsies was greater during chronic infection (*p* = 0.00257; Supplementary Data 13), but there was no significant difference between early and chronic infection when comparing total skin seeding (Fig. 5c and Supplementary Data 11). This indicates that localised parasite spreading is driven by a greater number of super-seeder biopsies, rather than an evenly distributed increase in seeding across all biopsies.

Network construction revealed that the skin-viscera interaction axis differs strikingly between individual mice (Fig. 5d–f and Supplementary Fig. 8). For example, in one mouse the bone marrow is acting as a parasite source for multiple skin biopsies (Fig. 5d). In another a single skin biopsy is the dominant source for other skin sites (Fig. 5e) and a third illustrates the capacity of skin to seed the liver (Fig. 5f). Analysis of in-degree and out-degree found no change between early and chronic infection for skin biopsies (Fig. 5g and Supplementary Data 10; Fig. 5h and Supplementary Data 9). Comparing eigen centrality, a measure of connectedness to other highly connected vertices in a network (see Fig. 4a), we saw a clear increase in the number of highly connected sites in chronic infection, although the average value across skin biopsies was not significantly different (Fig. 5i and Supplementary Data 15). This suggests that specific skin sites become more closely connected to the core dissemination network over time, despite exhibiting no change in average connection numbers.

### Immune perturbation via secondary infection alters skin seeding dynamics

**Fig. 6:**
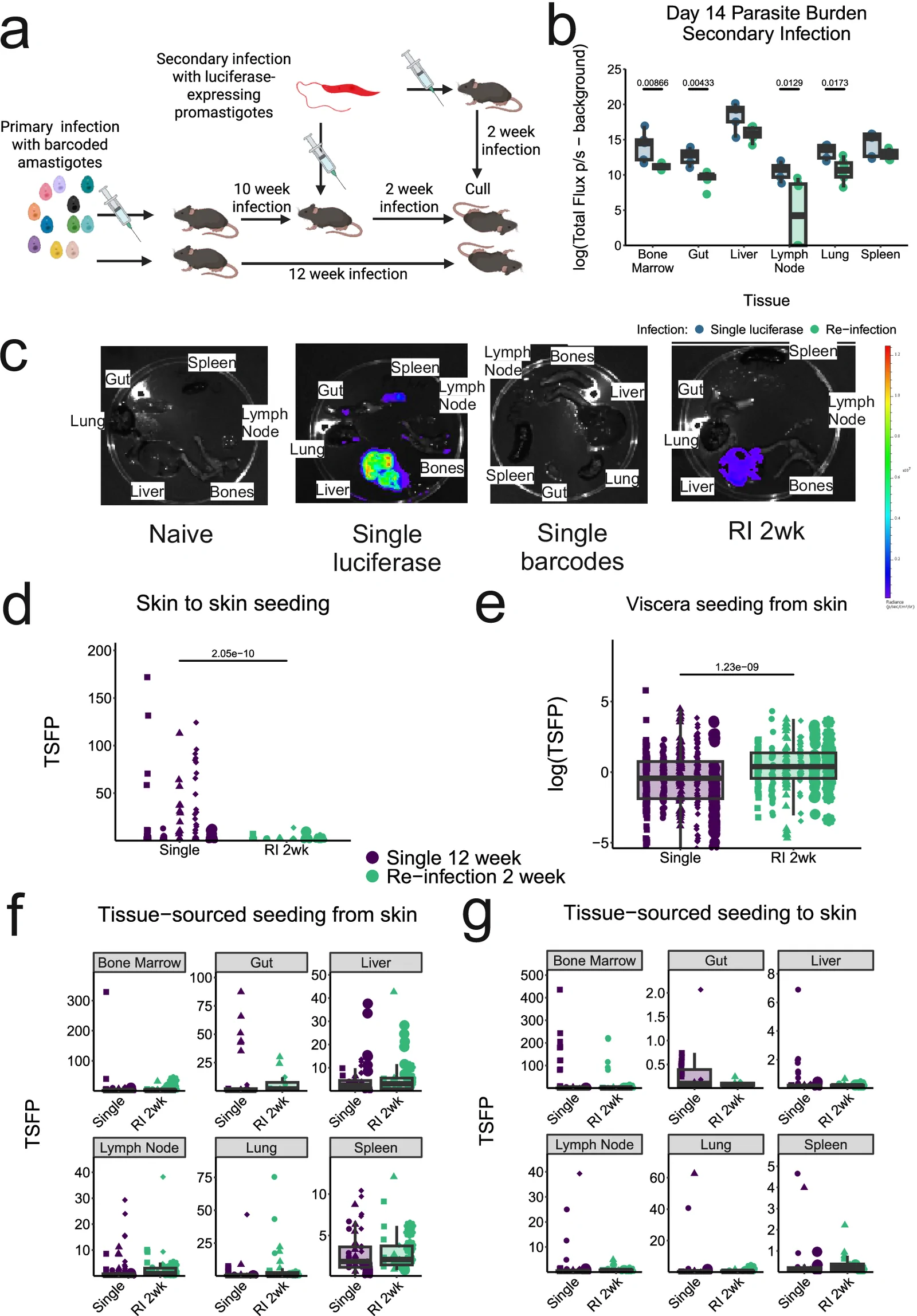
Secondary infection alters skin seeding dynamics to both local skin sites and visceral tissues. **a** Schematic of experimental design for re-infection of female C57BL/6 J mice. Single 12 week infection: *n* = 5 mice (also used as part of the previously described chronic infection group); 12 skin biopsies per mouse. Single 2 week infection: *n* = 5 mice. Re-infection (RI): *n* = 6 mice; 6 skin biopsies per mouse. Created in BioRender. Loughrey, C. (2026) (https://BioRender.com/7babrkx). **b** Luciferase signal (total flux p/s) minus naïve tissue background signal for visceral tissues for mice after 2 week infection with luciferase-expressing *L. donovani* (single infection; blue) or the same infection after prior infection with barcoded amastigote *L. donovani (*re-infection; green*)*. **c** Representative ex vivo IVIS images of visceral tissues post-cull. Colour scale indicates flux as indicated in the scale bar shown. **d** Tissue Sourced Founder Population (TSFP) for skin-to-skin seeding for each biopsy in the single (barcode only; purple) and re-infected (barcodes then luciferase; green) mice. **e** log(TSFP) for skin-to-visceral tissue seeding (amalgamated for all visceral tissues) for each skin biopsy in the single and re-infected mice. **f,** **g** TSFP for skin-to-visceral tissue seeding split by tissue recipient (**f**) and for visceral-to-skin tissue seeding split by tissue source (**g**) for each individual biopsy. Shape indicates individual mouse. The same colour legend applies to (**d**–**g**). All boxplots show the median, 25th and 75th quartiles, with whiskers representing all data-points within 1.5x IQR of the hinges; points above or below the whiskers represent outlying data values. *p* values from a two-sided Mann-Whitney-Wilcoxon test for total flux minus background (**b**) and for all TSFP skin biopsy pairs (**d**) and skin-viscera pairs (**e**) between the single (two week luciferase) infection group and the re-infection group (**b**) and for the single (12 week barcode) infection group and the re-infection group (**d** and **e**). IVIS total flux statistical testing (**b**): Bone Marrow *p* = 0.00866; Gut *p* = 0.00433; Lymph Nodes *p* = 0.0129; Lung *p* = 0.0173. Source data are provided as a Source Data file.

To explore the stability of these dissemination patterns, we investigated how they were affected by immune perturbation resulting from secondary infection. Mice infected for 10 weeks with barcoded amastigotes were re-infected with luciferase-expressing *L. donovani*. Uninfected mice were used as a control for infectivity of the luciferase-expressing parasites and to assess the degree of protective immunity (Fig. 6a and Supplementary Data 16). As expected, at two weeks p.i, ex vivo imaging indicated significant protection against secondary infection (Fig. 6b, c, Supplementary Fig. 10 and Supplementary Data 16).

Re-infection had notable effects on the primary barcoded parasite population. First, inter-skin seeding within a patch was significantly lower after re-challenge (Fig. 6d and Supplementary Data 17; MW test for TSFP of all biopsy pairs within each individual *p* < 0.0001; Supplementary Data 18; MW test for sub-sampled matched biopsy n number TSFP *p* < 0.001 in all cases), driven by a reduction in high-TSFP super-seeder events. Notably, we observed no evidence of seeding above our threshold of 20 within the skin for the re-infected mice (Supplementary Data 5). This could indicate a reduced skin parasite load or alternatively, a reduction only in parasite spread within the skin. Second, overall skin to visceral tissue trafficking was significantly increased after re-infection (MW test for TSFP of all biopsy-visceral tissue pairs within each individual and for sub-sampled matched biopsy *n* number TSFP *p* < 0.0001 in all cases; Fig. 6e; Supplementary Data 19 and Supplementary Data 20), suggesting the perturbed immune environment promoted parasite dissemination from the skin. Third, the impact of re-challenge on migration was tissue-specific (compound Poisson GLMM; Fig. 6f and Fig. 6g), whilst mouse-to-mouse heterogeneity was also a significant random effect (Supplementary Note). Collectively, these findings demonstrate a complex and extensive rerouting of dissemination pathways in response to immune perturbation. The skin appears to act as a dynamic parasite sanctuary, capable of re-seeding other tissues in specific immunological conditions, which may have important implications for disease relapse and transmission if translatable to the clinical context.

## Discussion

In this study, we have employed CRISPR genome editing to generate a neutral barcoded *L. donovani* library and combined this with a novel network-based analytical approach to examine the dissemination patterns of *L. donovani* in a murine model of VL. This network analysis, which extends previous STAMP^46^ methodology, allowed us to infer the likely directionality of parasite migration within the host, revealing a highly inter-related dissemination network connecting parasite populations across different tissues.

Our findings highlight three previously unrecognised features of the host-*Leishmania* interaction, which change the understanding of parasite persistence, relapse and transmission.

First, our network analysis demonstrates that the wide distribution of parasites across tissues, often viewed as a static consequence of systemic disease, is in fact merely a snapshot of a highly dynamic and inter-connected dissemination network. Although previous imaging studies using serial intra-vital imaging of *Leishmania*^65^ and *Trypanosoma cruzi*^66^ infections hinted at dynamic tissue tropism, our data formally establishes the inter-relationships between parasite population structures at distinct tissue sites. Our analytical approach allowed us to identify on an individual host basis the dominant dissemination highways and, notably, revealed probable bi-directional seeding of parasites both within and between visceral organs and skin. Surprisingly, within this highly inter-connected network, the bone marrow parasite population becomes increasingly isolated from populations in other visceral organs as the infection progresses. This finding aligns with reports of high parasitism of haematopoietic stem cells in experimental VL^8,9^ and the concept that the bone marrow serves as a protective sanctuary for other persistent pathogens, such as *Mycobacterium tuberculosis*^67^ and *Plasmodium* parasites^68^, suggesting a mechanism for long term sequestration. A greater understanding of the mechanics underlying parasite persistence and inter-organ seeding would, if translatable to humans, provide potential treatment targets for parasite elimination in affected patients, and could be of particular benefit to those most at risk of disease relapse.

Second, we observed frequent seeding of parasites to the skin, a finding consistent with reports in immunodeficient mice^17,64^, dogs^69^ and humans^70^. Extending our previous work identifying a highly heterogeneous patchy distribution of parasites in the skin^17,64^, we now show that parasite populations sampled within distinct biopsies across the skin are interconnected and frequently linked to one or more visceral organs. These observations reinforce a model whereby skin parasite patches originate by initial stochastic seeding from the viscera, followed by localised patch spread, ultimately creating larger inter-related patches. Importantly, our analysis also identifies potential super-seeder sites, which act as disproportionate sources for seeding of multiple other tissues. Our analysis of multiple skin biopsies indicates that super-seeding may be restricted to one or a few biopsies per mouse. Given the intracellular lifestyle of *Leishmania* within mammalian hosts, we hypothesise that super-seeding behaviour likely reflects the downstream consequences of events triggering mass migration of myeloid cells, including those containing parasites, into the draining lymphatics and subsequently the circulation.

Experimental testing of this hypothesis remains challenging, given the current inability to track the fate of individual skin patches over time, or to manipulate myeloid cell migration in a site-specific manner. However, inflammatory monocytes offer a specific target cell for investigation, given their established role in experimental VL infections^2,71^. Previous studies have identified quiescent parasites in CL skin lesions^28,29^ and our imaging data is suggestive of parasite phenotypic heterogeneity across skin patches, opening the possibility that super-seeder sites may contain a more actively dividing and diverse parasite population ready for export. Alternatively, the differences observed in signal and in migration potential across patches may relate to variation in death rates, due to heterogeneity in local immune responses, or may result solely from disparities in metabolic activities, uncoupled from replication rates. Interestingly, we note that most super-seeder sites did not display evidence of high levels of inward migration, implying that diversity within these locations results either from earlier inward migration which has since ceased, or high levels of initial colonisation as a result of permissive bottlenecks in the initial stages of infection. Identification of the cellular and molecular landscape that confers super-seeder status to some but not all sites is a major focus of current research, as is the role of super-seeder sites in facilitating transmission.

Our findings here open up avenues to investigate the parasite-level drivers of tissue tropism and migration, using dual-transcriptomic approaches and/or investigations of post-transcriptional regulation differences across tissues. Previous work found only minimal gene expression differences in *L. donovani* amastigotes in the liver and spleen^72^, but protein-level comparisons, and examination of other tissues, including skin, remain unexplored. Our barcode-insertion and detection approach can also be adapted to study the importance of candidate genes in the colonisation of divergent tissues, using a similar mutant-based bar-seq approach to that applied in previously published work in *L. mexicana*^40,41^. These types of studies would be of value both for understanding the fundamental biology of *Leishmania* and its interactions with the host, and identifying potential targets for intervention.

Although we extrapolate from a rodent intravenous infection model to humans with caution, it is reasonable to infer from our findings here that similar principles may apply to dissemination across species barriers. Further studies utilising sand fly challenge could address early stages of dissemination, whereas use of the hamster model could determine the impact on dissemination of end-stage disease. Pre-clinical and clinical development of therapeutics for VL mandate efficacy against parasites in the major visceral tissues and treatment options are generally not selected based on their distribution or efficacy within the skin^27^. However, our data supports a need for broader pharmacokinetics and pharmacodynamics studies which include sites of less immediate clinical significance, including asymptomatic skin, if there is an intention to minimise the likelihood of patient relapse and/or the generation of drug resistance in parasites^27^.

Our third key finding is the extreme malleability of *Leishmania* dissemination pathways in response to immune perturbation, with re-infection of immune mice promoting dissemination from the skin to visceral tissues. Although we have not formally addressed skin-specific protection in this model, the reduction in visceral parasite burden because of prior infection suggests protective immunity has been established. Whether skin-resident CD4^+^ T cells play a role in skin immunity in this model, as has been shown for *L. major*^73,74^, remains to be determined. Nevertheless, this counter-intuitive re-routing suggests that the local immune response to secondary infection triggers the export of parasites from the skin back to the viscera. Hence, the skin has the capacity to replenish parasite diversity in the viscera, indicating that dissemination patterns are not hardwired. Whether other non-cognate inflammatory responses act similarly to re-challenge is yet to be determined, but we speculate that the widespread heterogeneity in dissemination highways observed between individual mice could be a biological manifestation of an individual host’s immune history (i.e. the unique accumulation of inflammatory events, microbial dysbiosis, or co-infections^75^). Future studies should address whether any specific immunological alterations are noted to accompany specific re-routing patterns and investigate the mechanisms which drive altered host cell migration. Divergent dissemination patterns may explain patient-to-patient variation in treatment response and propensity for relapse and imply that successful targeting of persistent parasite populations to avoid these negative outcomes could require an individualised treatment approach.

Our study has some limitations. Firstly, the C57BL/6 J mouse model of VL does not progress to a fatal outcome. Hence, our results may not directly reflect dissemination patterns in patients with advanced disease. Further, the mouse strain is highly in-bred, and thus our conclusions may reflect elements of shared ancestry specific to this strain. Experiments were conducted using only female mice to avoid the potential confounding effects of inflammation caused by male aggressiveness. Hence, we cannot comment on whether dissemination patterns may exhibit sex-determined differences. Further, our experimental design analysed only a limited area of the skin for each mouse, and this may underestimate the full breadth of dissemination to, from and within the skin. Moreover, the invasive nature of current tissue sampling techniques means longitudinal tracking to determine whether super-seeder status is transient or a permanent feature of specific sites is currently not possible. Our analytical pipeline utilises an inference-based metric and cannot definitively identify migration events; identification of directional movement should therefore always be treated as probable and not proven. In an attempt to strengthen this identification, we have thus combined this probabilistic inference of direction with network MST analysis and other ecologically informed approaches to uncover key elements of dissemination in vivo.

In conclusion, our network-based analysis has provided an ecological framework for the investigation of parasite dissemination in experimental *L. donovani* infection. Further, this approach has applicability more broadly for the study of other infectious (and potentially non-infectious) diseases. By revealing the skin as a dynamic reservoir capable of re-seeding visceral tissues and showing how this network is re-routed by immune perturbation, we provide new insights that can be integrated into strategies for preventing relapse and controlling the spread of *Leishmania* and emerging drug resistance.

## Methods

### Ethical approval

All animal experiments received approval from the University of York Animal Welfare and Ethical Review Body (AWERB), were run in accordance with the Animals (Scientific Procedures) Act, 1986 and were conducted under Home Office Project Licence (#PP0326977; “Immunobiology of leishmaniasis”).

### Mice

All studies used female C57BL/6 J mice bred in-house at University of York, aged between 9 and 12 weeks old (barcode infections) or 15–17 weeks old (tdTomato infections). Mice were housed in Techniplast Emerald IVC cages with a light cycle running 7am-7pm daily, with temperatures maintained within 20–24 °C and 45–65% humidity. Note that infection timescales differ between the two experiments, with infections of 4 weeks and 10–12 weeks for barcode experiments and of 2 days, 4 weeks and 12 weeks for tdTomato infections. Parasites (see below) for mouse infections were freshly obtained from the spleens of long-term infected B6.*Rag2*^*-/-*^CD45.1 (RAG) mice as described previously^17^. Briefly, spleens were homogenised in RPMI, before centrifuging at 130 × g for 5 min. The supernatant was lysed with 25 mg saponin per 20 ml for 5 min at room temperature and was then centrifuged at 1900 × g for 10 min. The pellet was washed three times in RPMI, then resuspended to remove clumps using a 26-G needle before counting for resuspension. For infections using the barcoded library, 3 × 10^7^ *L. donovani* amastigotes were injected intravenously in 100 μl RPMI. For “re-infection”, mice were infected with 4–5 × 10^7^ stationary phase promastigote luciferase-expressing *L. donovani*. For confocal imaging experiments, mice were infected with 8 × 10^7^ amastigotes of the tdTomato-expressing *L. donovani* strain. Doses were selected based on previous experiments (both published^60,76^ and in pilot studies), to allow elucidation of biological mechanisms from our complex datasets.

### Ex vivo luciferase imaging

Tissues were imaged ex vivo immediately after removal from mice post-cull. Skin was shaved post-mortem and Veet hair removal cream was then applied for 10 min, before washing with water to remove residual hair. Skin was scraped prior to imaging to remove adipose tissue.

For ex vivo imaging, 30 mg/ml Firefly D-luciferin (Syd Labs: MB000102-R70170) in 1X PBS was applied to the surface of tissues, then incubated for 5 min in the dark. For skin, luciferin was applied to the hypodermal side. Imaging was performed with an IVIS Spectrum bioluminescence imaging system (PerkinElmer) with an open filter. Light detection was captured via a charge-coupled device (CCD) camera. Exposure was for up to 5 min for imaging and images were obtained and processed using Living Image version 4.7.4.21053; this constituted drawing regions of interest around each tissue to quantify flux for each sample. This data was then further analysed in R, by quantifying the background rate of flux for each tissue type using the mean flux obtained from naïve tissues; this background was subtracted from the flux for each infected tissue sample, to give a total flux above background level value for each tissue sample. Any negative values were set as 0. These values were then changed to a log scale, with any undefined values changed to 0.0000001, for the purposes of visualisation and statistical analysis.

### Confocal imaging in skin

For confocal imaging of mouse skin, the skin was shaved post-mortem and Veet was applied for 10 min, before washing with water. Mice were skinned, adipose tissue was removed and skin was imaged from the hypodermal side using an LS980 Upright Microscope (2.5X magnification; pin hole set to 500μm) using Zen version 3.9. Spectral fingerprinting based on a purified amastigote tdTomato *L. donovani* sample was used to generate a spectral profile for tdTomato, whereas an autofluorescence signal was derived from uninfected age matched control skin (Supplementary Fig. 11). Image processing was performed in Zen Blue Version 3.9.101.02000.

### In vitro infection of bone marrow derived macrophages

BMDMs were incubated with 5X multiplicity of infection (MOI) promastigote parasites at 37 °C for 1 or 3 h. Slides were fixed with methanol for 10 min and stained with 10% Giemsa stain for 3 min. The parasite burden per cell was then assessed by counting by eye for 100 host cells per condition. Poisson distributions were fitted to the parasite count per cell data for each barcoded parasite infection experiment at each time-point using the fitdist function in the fitdistrplus package in R^77^; scripts are available on Github^78^.

### Parasites

All *L. donovani* lines used were generated from the Ethiopian strain (MHOM/ET/1967/HU3), also referred to as LV9 or HU3. The *L. donovani* T7 Cas9 line used for the generation of the barcoded library was generated following the method described in ref. ^79^ for *L. infantum*. Briefly, the expression plasmid used (pTB010) enables expression of hSpCas9 and T7 RNA polymerase and contains homologous sequences that direct integration into the ribosomal locus of *Leishmania* spp. Promastigotes were transfected using 10 μg of linearised pTB010 using P3 Primary Cell 4D-Nucelofector (Lonza), with the single pulse programme FI-115 in a final volume of 110 μL. Cells were then transferred to a warmed medium and left to recover for 16 h before selection and cloning using complete solid SDM-79 with 150 μg/ml hygromycin. The luciferase-expressing parasite line *L. donovani* RE9H LV9 encoding a red-shifted firefly luciferase gene (Ppy-RE9H) in the ribosomal RNA locus^80,81^ and a tdTomato-expressing *L. donovani* strain^82–84^ were generated as described. Promastigote parasites were cultured at 25 °C in SDM-79 media (Gibco: 074-90916 N), supplemented with either 10% or 20% (for transfection recovery only) heat-inactivated FCS (Gibco: A5256701), with 5 μg/ml hemin (Sigma-Aldrich: 51280-5G), 10 μM 6-biopterin (Sigma-Aldrich: B2517-25MG), 100 U/ml penicillin and 100 μg/ml streptomycin.

### Development of the barcoded parasite library

Genome editing to insert the unique 20 bp barcodes was performed in the *L. donovani* strain constitutively expressing Cas9 and T7 RNA polymerase, generated following the protocol described in ref. ^79^ and briefly described above. The 20 bp barcode sequences were incorporated into the exogenous *HYG* locus used for insertion of the T7 and Cas9 expression cassette using reverse primers. The forward primer used was consistent for all insertions (OL12845). Two single guide RNAs (sgRNA) targeting the 5’ and 3’ insertion region within *HYG* were designed using the Eukaryotic Pathogen CRISPR guide RNA/DNA Design Tool (http://grna.ctegd.uga.edu). sgRNA templates and the repair cassettes (carrying a blasticidin resistance marker) were generated by PCR, using the CRISPR-Cas9 toolkit for kinetoplastids^85–87^. All primer sequences are provided in Supplementary Data 21 and 22; these were purchased from Merck and are available on request to the corresponding authors.

PCR products were purified using the QIAquick PCR Purification Kit (Qiagen: 28104). For transfection, sgRNA and the repair cassette was co-transfected into 2 × 10^6^ promastigotes using the P3 Primary Cell 4D-Nucleofector X Kit L (Lonza: V4XP-3024) with programme FI-115.

After electroporation, cells were immediately transferred to pre-warmed SDM-79 media, containing 20% FCS. Parasites were incubated at 25 °C with 150 μg/ml blasticidin initially, which was then reduced to 75 μg/ml for first passage and to 20 μg/ml for maintenance culture. Genomic DNA from recovered parasites was extracted for diagnostic PCR and successful barcode integration was visualised using a product size-based PCR (approximately 2 Kbp), using OL12882 and OL12884 primers (see Fig. 1a). Sanger sequencing of the initial 10 barcode insertions was also performed for validation.

### Barseq sequencing and analysis

Tissue samples were flash frozen on dry ice and stored at −80 °C. For bone marrow, the bones were flushed using un-supplemented RPMI with a 26 G needle; marrow was then centrifuged at 1900 × *g* for 10 min at room temperature. The DNA was extracted using Qiagen DNeasy Blood and Tissue Kits (Qiagen: 69506), following the tissue DNA extraction instructions for all samples except bone marrow and isolated or cultured parasite samples. For these two sample types, the cell culture instructions were followed. The 56 °C digestion step was performed for 10 min for the bone marrow and parasite samples, for 1–2 h for all tissue samples except skin, and overnight for skin.

For sequencing, the barcodes were amplified using a two-step PCR approach, before addition of Nextera XT Indexing primers. For the first PCR step, the amplification PCR used OL12833 and OL13833 and 1 unit Verifi Mix (PCR Biosystems: PB10.43-05), 0.4 nM 10 nm gold nanoparticles in citrate suspension (Sigma-Aldrich: 741957) and 0.025 mM MgCl_2_ (Sigma-Aldrich: M1028) in a 25 μl reaction, for 30 amplification cycles, with an annealing temperature of 60 °C and an extension time of 40 s. The first PCR product was then cleaned using 0.9X Agencourt AMPure XP beads (Beckman Coulter: A63882; see below). For the second PCR step, amplification of the purified product used OL14195 and OL14196 and 1 unit Verifi Mix (PCR Biosystems: PB10.43-05), 0.4 nM 10 nm gold nanoparticles in citrate suspension (Sigma-Aldrich: 741957) and 0.025 mM MgCl_2_ (Sigma-Aldrich: M1028) in a 25 μl reaction, for 6 amplification cycles, with an annealing temperature of 65 °C and an extension time of 40 s.

Samples were cleaned again using 0.9X Agencourt AMPure XP beads (Beckman Coulter: A63882). For the purification (both steps) samples were incubated with the beads at room temperature for 5 min. They were placed on a magnetic rack to clear, followed by removal of supernatant. Beads were then washed twice with 75% ethanol and dried for ~3 min before elution, using Molecular Biology Grade Water (ThermoFisher: AM9932) for 5 min at room temperature. For the library preparation, PCR products underwent 9 PCR amplification cycles with Q5 Polymerase 2X Master Mix (New England Biolabs: M0492L), following the manufacturer instructions. Sample purification was performed using 0.9X Agencourt AMPure XP beads (Beckman Coulter: A63882), eluted into a low TE buffer, and pooled samples were then sent to Azenta Life Sciences for 150 base paired end sequencing using an Illumina Sequencer.

### MATLAB simulation

To assess the accuracy of STAMP FP estimation for different sizes of barcode library (Fig. 1b,  c), we used MATLAB/ 2024a to simulate barcoded parasites passing through a range of bottlenecks. An input pool of barcoded parasites was generated as a matrix of 5 × 10^7^ individuals, with an even number allocated to each barcode, for barcode totals ranging from 10 to 500 in total. Bottlenecks were simulated as a probability of an individual parasite “passing” the obstacle, ranging from 0.0000001 to 0.5. The simulation was repeated for 100 “experiments” for each bottleneck and barcode total combination.

We initially ran a basic simulation assuming all barcoded parasites replicated at the same rate post-bottleneck, by counting the individuals with each barcode after the bottleneck was applied. We then refined the simulation to reflect a 5% random variation in overall growth rates, by multiplying the total count of individuals post-bottleneck for each barcode by a randomly generated number between 0.95 and 1.05.

For the simulation of clonal expansion, the same procedure as the 5% random variance simulation was followed, but immediately after the bottleneck, we randomly selected 1 in 100 (common), 1 in 10000 (medium) or 1 in 1000000 (rare) individuals to clonally expand, by multiplying their count by 10 to simulate a growth rate for these individual parasites of 10x the average. We then used the counted total of all passing parasites as our true FP and computed FP for our STAMP-estimated FP, using the method defined by Abel et al.^46^, based on original equations from Krimbas and Tsakas^48^. The equation used was as follows:

1 $$\hat{F}=\frac{1}{k}{\sum }_{i=1}^{k}{\left({f}_{i,s}-{f}_{i,0}\right)}^{2}/{f}_{i,0}\left(1-{f}_{i,0}\right)$$

2 $${{{\rm{FP}}}}=\frac{1}{\hat{F}-\frac{1}{{S}_{0}}-\frac{1}{{S}_{s}}}$$

where *f*_*i*_ is the frequency of barcode *i*, at input (*f*_*i, 0*_) and after the bottleneck (*f*_*i, S*_), *S*_*0*_ and *S*_*S*_ are the total parasite counts for each, and *k* is the total number of barcodes. The original equations contain a generation term, *g*, which for our analysis we set as 1. The accuracy of the estimation was then assessed via Kendall correlation, using the cor.test function in R. The MATLAB script for the simulation and R scripts for the analysis are available in Github^78^ (see Data and Code Availability sections for details).

### Barcode quantification

Barcode sequence counts were extracted using a Rust script (available on Github^88^; see Data and Code Availability sections for details; Rust version 0.2.1). This script used the primer sequence directly before the barcodes to pull out 20 bp barcode sequences from all forward reads. Barcodes with each sequence were counted and those with 500 or fewer reads were then merged with the barcode above the 500 read threshold from which they each had the lowest Levenshtein distance. If they were not within 5 Levenshtein edit distances of any barcodes above the 500 read threshold, they would not be merged for counting. An R script (available on Github^88^) which pulled out only the canonical barcode sequences from this list of counts was used to generate the analysis matrices of barcode counts for all samples analysed. Prior to subsequent analysis, the barcode with sequence TGGATCTTAGGACGCAACAT was removed due to indications in preliminary studies (Loughrey, 2024^89^) that it exhibited an usually high abundance in an unexpectedly large number of tissue samples across multiple experiments.

### Diversity analysis

Within tissue parasite diversity was quantified using the Shannon Index, calculated using the Vegan package in R^90^ (supplied in Source Data files). The Shannon Index was quantified as:

3 $$-{\sum }_{i=1}^{S}{p}_{i}\,{\log }_{b}\,{p}_{i}$$

where *p*_*i*_ is proportion of species *i* (in this case barcodes) and *S* is total number of species (barcode total) and *b* is the default logarithm base option in Vegan (natural logarithm *e*), as defined by Hill^62^. Analysis scripts are available on Github^78^ (see Data and Code Availability sections for details).

### Genetic distance analysis

GD (*D*_*ch*_) was calculated as Cavalli-Sforza chord distance^47^ as shown:

4 $${D}_{{ch}}=\frac{2\sqrt{2}}{\pi }\sqrt{1-\cos \theta }$$

5 $$\cos \theta={\sum }_{i=1}^{k}\sqrt{{f}_{P1,i}\,{f}_{P2,i}}$$

where *f*_*i*_ is the frequency of barcode *i* in tissue parasite populations *P1* and *P2* and *k* is total number of barcodes. When using GD to build full networks, we used GD to initially structure the networks, then used 1-GD (i.e. genetic relatedness) for edge weights for visualisation. All GDs of above 0.5 were removed for the purposes of network visualisation in Fig. 3 and Supplementary Fig. 5. However, this cut-off was not applied to any statistical analysis and was purely used for the purpose of visualising the connections which were strongest in our network figures. This calculation and network generation was done using custom R code available on Github^78^ (see Data and Code Availability sections for details).

### Founder Population metrics and network analysis

The evidence we had of serial bottlenecks and a highly interconnected network of parasite populations led us to speculate about the direction of parasite movements between tissues, and as such we decided to calculate FP from tissue sources to tissue recipients for all pairwise comparisons within each individual mouse (see Fig. 4a). We termed this directional metric Tissue-sourced FP (TSFP). Before calculating this value, we removed all samples with total barcode reads of below 50000, as samples below this cut off confounded the calculation due to the large difference between *S*_*0*_ and *S*_*S*_; removed samples are noted in Supplementary Data 23. Sample barcode counts were then filtered for each individual comparison pair in order to remove all barcodes with a count of 0 in the source tissue from both samples being analysed (Supplementary Data 24 provides the total number of barcodes included in the TSFP calculation for each sample after this removal step). TSFP was calculated based on the STAMP method^46,48^ as described below:

6 $$\hat{F}=\frac{1}{k}{\sum }_{i=1}^{k}{\left({f}_{i,s}-{f}_{i,0}\right)}^{2}/{f}_{i,0}(1-{f}_{i,0})$$

7 $${{{\rm{TSFP}}}}=\frac{1}{\hat{F}-\frac{1}{{S}_{0}}-\frac{1}{{S}_{s}}}$$

where *f*_*i*_ is the frequency of barcode *i*, in the input source tissue (*f*_*i, 0*_) and in the tissue recipient (*f*_*i, S*_), *S*_*0*_ and *S*_*S*_ are the total barcode reads for each sample, and *k* is the total number of barcodes included in the analysis.

To calculate the overall directional movement of parasites, we found the difference between TSFP in each direction for each tissue pair and used the positive value to build a network of directional parasite movements. We termed this value Fractional FP (FFP). For network construction and analysis, we used the igraph package in R^91,92^, with ggraph^93^ used for generating the final figures. FFP was used as the edge weights for building full networks. For computation of minimum spanning trees (MSTs), we used 1/FFP as edge weights. In both cases, we removed all edges with FFP below 20. For analysis of degree (in and out), we used the igraph degree function on the full network. For eigen centrality analysis, we used the eigen_centrality function in igraph on the undirected full graph. All scripts are available on Github^78^ (see Data and Code Availability sections for details).

### Validation

To validate our analysis of TSFP and FFP, we first assessed the likelihood of false migration detection in randomised datasets. To do this, we took our original barcode count matrix and randomly reallocated the counts for each barcode to another barcode in our list, across all samples in the dataset. We repeated this for three simulated datasets in total (provided in Source Data files). We reasoned that this approach preserved the number of barcodes and count distributions for tissue populations of parasites, but randomisation meant they were not related to each other. We calculated pairwise TSFP and FFP for each sample and calculated the percentage of values which were above a set of threshold FFPs, ranging from 1 to 500 (Supplementary Data 25), using the AboveThreshold function in the Seurat R package^94–98^. We noted that the percentages above thresholds were very low in all three simulations when compared to our actual dataset of within-mouse FFPs.

We then used our INPUT samples to create a threshold value for confidence of FFP. We reasoned here that our populations within tissues cannot truly be sources of the INPUT pool, whilst the INPUT pool must be the ultimate source for at least part of the population within our tissue samples, although this could constitute a very small proportion of the end population under circumstances of very restrictive bottlenecks, high inter-tissue migration and/or selective pressures affecting replication dynamics. We therefore calculated TSFP and FFP in both a true migration direction, defined as INPUT pool (population A) to sample (population B) and a back migration direction, defined as sample (population B) to INPUT pool (population A) for all combinations of our samples and INPUT pools. Supplementary Figs 12a and 12b show the histograms for TSFP and FFP respectively. For visualisation on a log scale, we changed all FFP values below 1 to 0.9 and all negative TSFP values and any NA values to 0. Using these histograms, we defined our FFP threshold for our network and MST creation as those above 20 (log3 approximately), based on the shift between true and back migration distributions we observed. We additionally used this analysis to compare the true vs back migration above threshold for each tissue type (Supplementary Data 26 and 27), as we hypothesised that this would vary by tissue due to underlying differences in both population structures and in relatedness to the original INPUT pool itself, due to initial colonisation dynamics. We confirmed this to be the case, meaning we cannot establish definitive false positive rates for our metric, as this will be affected by variables such as sample type and infection model used. Importantly we show here that whilst tissues such as spleen show relatively high false migration, as predicted, they show consistently higher levels of true migration. Of note in terms of our work’s conclusions, we also show that the skin shows no evidence of any false migrations above our threshold of 20. We also validated that threshold choice did not affect the main conclusions in our manuscript by running our network analysis with varying thresholds applied and comparing the centrality metrics derived in each case. A comparison of the statistical analysis of these thresholds is included in Supplementary Data 28, 29 and 30.

We further validated our approach by applying a jack-knife analysis, whereby we iteratively removed each barcode in turn and recalculated the FFP, then compared the magnitude and direction to the value when calculated using all barcodes. We found that the directionality was preserved in over 99% of cases, although removal of a few specific barcodes showed lower conservation of direction in FFP (Supplementary Data 31). The magnitude of FFP would be anticipated to change when barcodes are removed, due to the inherent difference in the possible pool of founders created when reducing the input population; however, overall we noted a strong correlation to FFP calculated with all barcodes (Tau = 0.987; *p* < 0.0001; Supplementary Fig. 13 and Supplementary Note). We further assessed the correlation when removing random subsets of barcodes amounting to variable percentages of the total barcode pool (95, 90, 75 and 50%); as expected, we noted that the correlation of magnitude of FFP decreased as the barcode pool decreased (Supplementary Data 32), as did the match in directionality (Supplementary Note). However, both remained broadly consistent even when relatively large proportions of barcodes were removed.

To quantify the uncertainty in our TSFP calculations as a result of sequencing read depth variance, we performed a down-sampling analysis in which we randomly removed a percentage of the raw fastq reads prior to barcode extraction, using the sample command in SeqKit (version 2.3.1). We used this to randomly remove 10% of reads (leaving 90% for subsequent analysis), with 5 repeat runs to test the variation in TSFP estimate obtained. We then ran the down-sampled reads through the analysis pipeline, and compared the TSFP values for tissue pairs to the values obtained when we used all fastq reads (no down-sampling). With 90% read depth, we found a 96% correlation between these TSFP values and those calculated with 100% of reads (Supplementary Fig. 14, Supplementary Note and Supplementary Data 33). We repeated this approach with 50% of reads removed and with 25% of reads removed and likewise noted very high correlations in both cases (87% and 93% respectively; Supplementary Fig. 14, Supplementary Note and Supplementary Data 33). As expected, with 50% reads we found that we had a larger number of NA samples (those where total reads fell below our sample removal threshold). It should also be noted that removal of reads inherently changes the computed TSFP magnitude compared to the full set of reads, as the initial input pool is reduced, altering relative values of TSFP calculated. However, our calculations show overall high consistency in calculation of TSFP with this caveat in mind, even with large reductions in sequencing depth.

To further provide confidence in our analysis approach, we tested it on three published datasets^33,50,55^ (Supplementary Fig. 15). From Hotinger et al^55^ (Supplementary Fig. 15a), we used a dataset of multiple samples from 73 individual mice, with a set of individual inoculums for different infection groups, some of which had up to three replicate samples; in this case, we matched inoculum to the correct mice for our analysis. From Lebrun-Corbin et al.^50^ (Supplementary Fig. 15b), we used a set of multiple samples from 12 mice, with 30 replicate INPUT inoculum samples. From Hullahalli and Waldor^33^ (Supplementary Fig. 15c), we used a set of multiple samples from 32 mice plus 5 INPUT pools as replicates of a single inoculum.

Whilst there are biological insights to these analyses, these are beyond the scope of this work. Supplementary Fig. 15 shows the thresholding histograms for these datasets, which we find illustrative of three key points. Firstly, that our analytical pipeline can be effectively implemented on a range of microbial population datasets, across diverse tissues, pathogens and infection model conditions. Secondly, that a skewed barcode distribution (such as that induced by the amastigote passage process our parasites underwent) does not seem to hinder, and in fact may be beneficial for, threshold determination, as evidenced by the clear distribution shift in our data (Supplementary Fig. 12). Library size (which differed widely between the datasets) also does not appear to affect thresholding clarity; we hypothesise that the dynamics of within-host replication patterns may play a role in the ability to clearly discern directionality, given that Hullahalli and Waldor specifically report on clonal expansion occurrences in their dataset hampering FP calculations^33^. Thirdly, thresholds appear to vary between experiments. This is likely related to the biological aspects of the systems in question, given they all utilise different pathogen strains with divergent initial colonisation patterns and specific sample types measured. However, a smaller library size does not seem to reduce threshold discernment in itself.

We were cognizant that background similarity of populations is inherent in systems derived from a common ancestor population, and that we cannot directly measure the initial similarity of tissue populations upon initial colonisation within a single animal immediately after injection to account for this. This is an inherent limitation of population dynamics for the study of dissemination in vivo, regardless of metric choice. The only scenarios in which it can be completely avoided are one in which there is no intermixing or migration to measure to begin with combined with a very high level of initial diversity or very restrictive initial bottlenecks to individual tissue colonisation (i.e. no overlaps in barcode presence between tissues at all), in which case there is no dissemination to measure, or alternatively by using a system in which barcode expression or editing is induced in situ after initial colonisation.

Therefore, we also used our dataset to calculate TSFP and FFP for a matched number of randomly selected comparisons of samples from disparate mice and compared these to our within-mouse values for each metric (Supplementary Fig. 16). When comparing density distributions for all values above our threshold of 20, we observed that, as expected, background similarity of populations generates an inference of migration. However, we note a sustained shift in the distribution of the within-mouse comparisons towards increased TSFP (Supplementary Fig. 16a) and FFP (Supplementary Fig. 16b), giving us confidence that we were detecting true dissemination dynamics. This relatedness confounder likely differs between model systems depending on initial bottleneck events, population structural differences inherent to different niches and with the relative complexity of inter-seeding patterns. Importantly, it will apply even in experiments with larger library sizes (although it may be missed in these cases, if using between animal comparisons for detection) and with the use of pre-established population dynamics metrics such as Cavalli-Sforza chord distance. Therefore, experimental design should incorporate time-based comparisons between groups, interpretations should be informed by biological knowledge of the system in question and of biologically plausible relationships, whilst analysis approaches should consider using a combination of metrics with network analysis, including MSTs, for more robust determination of dissemination pathways.

All datasets and R scripts for this validation are supplied on Github^78,88^ (see Data and Code Availability sections for details).

### Statistics and reproducibility

Only female mice were used to avoid non-specific tissue damage in the skin caused by fighting in cages of male mice. One mouse was excluded from further analysis after dissection due to an absence of hepatosplenomegaly (which would be evidence of established infection at 4 weeks p.i). One mouse was killed early due to welfare concerns and was excluded from subsequent analysis.

Samples with fewer than 50000 reads for canonical barcode sequences were removed from TSFP and FFP analysis (but were used for SI and GD analysis). Sample read counts are supplied in Supplementary Data 23. Statistical tests for TSFP (including GLMM fitting) were performed on data after removal of samples with fewer than 50000 read counts.

Animal experiments were performed using the same pool of barcoded amastigotes extracted from passage mice. Differences in the barcode frequencies between pools derived from separate mice that arises from bottlenecks associated with passage ruled out amalgamation of replicate experiments and direct comparison for Barseq-based statistical analysis. Sample sizes are given in Results and/or Figure legends. The single infection group used for Barseq statistical comparison to re-infection comprised 5 mice which were infected for 12 weeks and were also used in the chronic infection group; the remaining three mice in this group were not used for comparison to re-infection as they were infected for 10 weeks.

For Barseq analysis for each mouse, a single sample was sequenced for liver, spleen, gut and lung, whilst we used two separate inguinal lymph nodes and femur/tibia, each processed separately. For skin 6–12 biopsies were taken per mouse as indicated in Results and/or Figure legends, and were again each processed individually.

Data processing, statistical analysis and figure generation were performed using the tidyverse^99^, ggraph, ggpubr^100^, igraph, fitdisrplus, naniar^101^, Seurat and rstatix^102^ packages in R version 4.4.3. Mann-Whitney-Wilcoxon tests (two sided) were used for in vivo parasite population comparisons of diversity (Shannon Index), Genetic Distance and TSFP where indicated in figure legends. When analysing the skin biopsies in single vs re-infection, we performed sub-sampling using six randomly selected biopsies from each single-infection mouse (with *n* = 5 replicates performed), to validate that differences in n number were not responsible for our statistical findings.

We used a Compound Poisson GLMM to analyse the fixed effect of time p.i (early vs late) on parasite diversity (Shannon Index) across all skin biopsies for each mouse, with mouse ID as a random effect. All GLMMs were fitted and compared using the cplm and tweedie packages^103–106^ in R. We compared models with both effects and with only mouse ID or only time p.i using the gini function in cplm. This approach fits a Lorenz curve and calculates the Gini index for each model fit against this curve; selecting the model with the lowest maximal absolute Gini index when compared against all other models thus gives us the closest model fit to our dataset^107^.

We likewise used a Compound Poisson GLMM to compare models for Genetic Distance between visceral tissues and skin biopsies, and for model comparisons for TSFP to viscera from skin biopsies and vice versa. For these models, we used time p.i (Genetic Distance only) and reinfection vs single infection (TSFP only) plus visceral tissue identity as the fixed effects and mouse ID again as the random effect. Model comparison was performed in the same manner as described above, but models tested included the interaction of the two fixed effects plus the random effect, the same model but without interaction of fixed effects, and then a range of models with each effect removed. All GLMM model outputs and comparisons are included in the Supplementary Note.

### Reporting summary

Further information on research design is available in the Nature Portfolio Reporting Summary linked to this article.

## Supplementary information

Supplementary information

Description of Additional Supplementary File

Supplementary Data

Reporting Summary

Transparent Peer Review file

## Source data

Source Data

## Data Availability

The Illumina sequence data generated in this study have been deposited in the NCBI SRA database under accession code PRJNA1392034 (http://www.ncbi.nlm.nih.gov/bioproject/1392034). The processed barcode counts data are available in Source Data and on Github^78^ at: https://github.com/CiaraLoughrey-scientist/barseq-Leishmania-2025. All other data generated in this study are provided in the Supplementary Information/Source Data file, including raw gel and IVIS images. Summary statistical data are available as supplementary data files with this manuscript. Raw image files for confocal skin imaging are available on request via email to the corresponding or first authors, due to size limitations. Source data are provided with this paper. For reanalysis of three published datasets, the datasets were obtained from that supplied with the publications, in the case of Lebrun-Corbin et al.^50^ and Hullahalli and Waldor^33^, and via email correspondence with the authors of Hotinger et al^55^. We have supplied the processed FFP calculation data in Source Data. Schematics were created using Biorender. Source data are provided with this paper.

## Code availability

All analysis scripts are provided in the Github^78^ repository at: (https://github.com/CiaraLoughrey-scientist/barseq-Leishmania-2025).

## References

[1] Romano, A. et al. Divergent roles for Ly6C+CCR2+CX3CR1+ inflammatory monocytes during primary or secondary infection of the skin with the intra-phagosomal pathogen Leishmania major. *PLoS Pathog.* **13**, e1006479 (2017). 10.1371/journal.ppat.1006479 PMC5509374 28666021

[2] Terrazas, C. et al. Ly6Chi inflammatory monocytes promote susceptibility to Leishmania donovani infection. *Sci. Rep.* **7**, 14693 (2017). 10.1038/s41598-017-14935-3 PMC5665970 29089636

[3] Engwerda, C. R., Ato, M. & Kaye, P. M. Macrophages, pathology and parasite persistence in experimental visceral leishmaniasis. *Trends Parasitol.* **20**, 524–530 (2004). 10.1016/j.pt.2004.08.009 15471704

[4] Laufs, H. et al. Intracellular survival of Leishmania major in neutrophil granulocytes after uptake in the absence of heat-labile serum factors. *Infect. Immun.* **70**, 826–835 (2002). 10.1128/iai.70.2.826-835.2002 PMC127667 11796617

[5] Chang, K. P. Leishmanicidal mechanisms of human polymorphonuclear phagocytes. *Am. J. Trop. Med. Hyg.* **30**, 322–333 (1981). 10.4269/ajtmh.1981.30.322 7235125

[6] Carlsen, E. D. et al. Permissive and protective roles for neutrophils in leishmaniasis. *Clin. Exp. Immunol.* **182**, 109–118 (2015). 10.1111/cei.12674 PMC4608500 26126690

[7] Oualha, R. et al. Infection of human neutrophils with leishmania infantum or leishmania major strains triggers activation and differential cytokines release. *Front. Cell. Infect. Microbiol.* **9**, 153 (2019). 10.3389/fcimb.2019.00153 PMC6524560 31134162

[8] Dirkx, L. et al. Long-term hematopoietic stem cells trigger quiescence in Leishmania parasites. *PLoS Pathog.* **20**, e1012181 (2024). 10.1371/journal.ppat.1012181 PMC11073788 38656959

[9] Dirkx, L. et al. Long-term hematopoietic stem cells as a parasite niche during treatment failure in visceral leishmaniasis. *Commun. Biol.* **5**, 626 (2022). 10.1038/s42003-022-03591-7 PMC9233693 35752645

[10] Bogdan, C. et al. Fibroblasts as host cells in latent leishmaniosis. *J. Exp. Med.* **191**, 2121–2130 (2000). 10.1084/jem.191.12.2121 PMC2193203 10859337

[11] Minero, M. A., Chinchilla, M., Guerrero, O. M. & Castro, A. [Infection of skin fibroblasts in animals with different levels of sensitivity to Leishmania infantum and Leishmania mexicana (Kinetoplastida: Trypanosomatidae)]. *Rev. Biol. Trop.* **52**, 261–267 (2004). 17357424

[12] Kaye, P. & Scott, P. Leishmaniasis: complexity at the host-pathogen interface. *Nat. Rev. Microbiol.* **9**, 604–615 (2011). 10.1038/nrmicro2608 21747391

[13] Kaye, P. M. et al. The immunopathology of experimental visceral leishmaniasis. *Immunol. Rev.* **201**, 239–253 (2004). 10.1111/j.0105-2896.2004.00188.x 15361245

[14] Lewis, M. D. et al. Fatal progression of experimental visceral leishmaniasis is associated with intestinal parasitism and secondary infection by commensal bacteria, and is delayed by antibiotic prophylaxis. *PLoS Pathog.* **16**, e1008456 (2020). 10.1371/journal.ppat.1008456 PMC7179947 32282850

[15] Costa, D. J. et al. Experimental infection of dogs with leishmania and saliva as a model to study canine visceral leishmaniasis. *PLoS ONE* **8**, e60535 (2013). 10.1371/journal.pone.0060535 PMC3618420 23577121

[16] Ibrahim, M. K. et al. The malnutrition-related increase in early visceralization of leishmania donovani is associated with a reduced number of lymph node phagocytes and altered conduit system flow. *PLoS Negl. Trop. Dis.* **7**, e2329 (2013). 10.1371/journal.pntd.0002329 PMC3744437 23967356

[17] Doehl, J. S. P. et al. Skin parasite landscape determines host infectiousness in visceral leishmaniasis. *Nat. Commun.* **8**, 57 (2017). 10.1038/s41467-017-00103-8 PMC5498584 28680146

[18] de Oca, M. M. et al. UVB modifies skin immune-stroma cross-talk and promotes effector T cell recruitment during cryptic *Leishmania**donovani* infection. *bioRxiv* 10.1101/2023.02.03.526940 (2023).

[19] Silva, L. C. et al. Canine visceral leishmaniasis as a systemic fibrotic disease. *Int. J. Exp. Pathol.* **94**, 133–143 (2013). 10.1111/iep.12010 PMC3607142 23419132

[20] Zijlstra, E. E. PKDL and other dermal lesions in HIV co-infected patients with Leishmaniasis: review of clinical presentation in relation to immune responses. *PLoS Negl. Trop. Dis.* **8**, e3258 (2014). 10.1371/journal.pntd.0003258 PMC4238984 25412435

[21] Alvar, J. et al. The relationship between Leishmaniasis and AIDS: the Second 10 Years. *Clin. Microbiol. Rev.* **21**, 334–359 (2008). 10.1128/CMR.00061-07 PMC2292576 18400800

[22] Rosenthal, E. et al. HIV and *Leishmania* coinfection: a review of 91 cases with focus on atypical locations of *Leishmania*. *Clin. Infect. Dis.* **31**, 1093–1095 (2000). 10.1086/318135 11049794

[23] Arumugam, S., Scorza, B. M. & Petersen, C. Visceral leishmaniasis and the skin: dermal parasite transmission to sand flies. *Pathog. Basel Switz.* **11**, 610 (2022). 10.3390/pathogens11060610 PMC9228576 35745464

[24] Zijlstra, E. E. The immunology of post-kala-azar dermal leishmaniasis (PKDL). *Parasit. Vectors* **9**, 464 (2016). 10.1186/s13071-016-1721-0 PMC4995613 27553063

[25] Mukhopadhyay, D., Dalton, J. E., Kaye, P. M. & Chatterjee, M. Post kala-azar dermal leishmaniasis: an unresolved mystery. *Trends Parasitol.* **30**, 65–74 (2014). 10.1016/j.pt.2013.12.004 PMC3919212 24388776

[26] Zijlstra, E., Musa, A., Khalil, E., El Hassan, I. & El-Hassan, A. Post-kala-azar dermal leishmaniasis. *Lancet Infect. Dis.* **3**, 87–98 (2003). 10.1016/s1473-3099(03)00517-6 12560194

[27] Wijnant, G.-J. et al. Tackling drug resistance and other causes of treatment failure in leishmaniasis. *Front. Trop. Dis.* **3**, 837460 (2022).

[28] Kloehn, J. et al. Identification of metabolically quiescent Leishmania mexicana parasites in peripheral and cured dermal granulomas using stable isotope tracing imaging mass spectrometry. *mBio* **12**, e00129-21 (2021). 10.1128/mBio.00129-21 PMC8092208 33824211

[29] Kloehn, J., Saunders, E. C., O’Callaghan, S., Dagley, M. J. & McConville, M. J. Characterization of metabolically quiescent Leishmania parasites in murine lesions using heavy water labeling. *PLoS Pathog.* **11**, e1004683 (2015). 10.1371/journal.ppat.1004683 PMC4340956 25714830

[30] Restif, O. & Graham, A. L. Within-host dynamics of infection: from ecological insights to evolutionary predictions. *Philos. Trans. R. Soc. B Biol. Sci.* **370**, 20140304 (2015). 10.1098/rstb.2014.0304 PMC4528502 26150670

[31] Abel, S., Abel zur Wiesch, P., Davis, B. M. & Waldor, M. K. Analysis of bottlenecks in experimental models of infection. *PLoS Pathog.* **11**, e1004823 (2015). 10.1371/journal.ppat.1004823 PMC4465827 26066486

[32] Grenfell, B. T. et al. Unifying the epidemiological and evolutionary dynamics of pathogens. *Science* **303**, 327–332 (2004). 10.1126/science.1090727 14726583

[33] Hullahalli, K. & Waldor, M. K. Pathogen clonal expansion underlies multiorgan dissemination and organ-specific outcomes during murine systemic infection. *eLife* **10**, e70910 (2021). 10.7554/eLife.70910 PMC8545400 34636322

[34] Campbell, I. W., Hullahalli, K. & Waldor, M. K. Quantifying host-microbe interactions with bacterial lineage tracing. *Science* **391**, 34–40 (2026). 10.1126/science.adx5362 PMC12854280 41477882

[35] Jang, M. J. et al. Spatial transcriptomics for profiling the tropism of viral vectors in tissues. *Nat. Biotechnol.* **41**, 1272–1286 (2023). 10.1038/s41587-022-01648-w PMC10443732 36702899

[36] Ratz, M. et al. Clonal relations in the mouse brain revealed by single-cell and spatial transcriptomics. *Nat. Neurosci.* **25**, 285–294 (2022). 10.1038/s41593-022-01011-x PMC8904259 35210624

[37] Askary, A. et al. In situ readout of DNA barcodes and single base edits facilitated by in vitro transcription. *Nat. Biotechnol.* **38**, 66–75 (2020). 10.1038/s41587-019-0299-4 PMC6954335 31740838

[38] Quinn, J. J. et al. Single-cell lineages reveal the rates, routes, and drivers of metastasis in cancer xenografts. *Science* **371**, eabc1944 (2021). 10.1126/science.abc1944 PMC7983364 33479121

[39] Vasquez, K. S. et al. Quantifying rapid bacterial evolution and transmission within the mouse intestine. *Cell Host Microbe* **29**, 1454–1468.e4 (2021). 10.1016/j.chom.2021.08.003 PMC8445907 34473943

[40] Baker, N. et al. Systematic functional analysis of Leishmania protein kinases identifies regulators of differentiation or survival. *Nat. Commun.* **12**, 1244 (2021). 10.1038/s41467-021-21360-8 PMC7902614 33623024

[41] Damianou, A. et al. Essential roles for deubiquitination in Leishmania life cycle progression. *PLoS Pathog.* **16**, e1008455 (2020). 10.1371/journal.ppat.1008455 PMC7319358 32544189

[42] Burge, R. J., Damianou, A., Wilkinson, A. J., Rodenko, B. & Mottram, J. C. Leishmania differentiation requires ubiquitin conjugation mediated by a UBC2-UEV1 E2 complex. *PLoS Pathog.* **16**, e1008784 (2020). 10.1371/journal.ppat.1008784 PMC7647121 33108402

[43] Bushell, E. et al. Functional profiling of a plasmodium genome reveals an abundance of essential genes. *Cell* **170**, 260–272.e8 (2017). 10.1016/j.cell.2017.06.030 PMC5509546 28708996

[44] Gomes, A. R. et al. A genome-scale vector resource enables high-throughput reverse genetic screening in a malaria parasite. *Cell Host Microbe* **17**, 404–413 (2015). 10.1016/j.chom.2015.01.014 PMC4362957 25732065

[45] Beneke, T. et al. Genetic dissection of a Leishmania flagellar proteome demonstrates requirement for directional motility in sand fly infections. *PLoS Pathog.* **15**, e1007828 (2019). 10.1371/journal.ppat.1007828 PMC6615630 31242261

[46] Abel, S. et al. Sequence tag-based analysis of microbial population dynamics. *Nat. Methods* **12**, 223–226 (2015). 3 p following 226. 10.1038/nmeth.3253 PMC4344388 25599549

[47] Cavalli-Sforza, L. L. & Edwards, A. W. Phylogenetic analysis. models and estimation procedures. *Am. J. Hum. Genet.* **19**, 233–257 (1967). PMC1706274 6026583

[48] Krimbas, C. B. & Tsakas, S. The genetics of dacus oleae. V. changes of esterase polymorphism in a natural population following insecticide control-selection or drift? *Evol. Int. J. Org. Evol.* **25**, 454–460 (1971). 10.1111/j.1558-5646.1971.tb01904.x 28565021

[49] Bachta, K. E. R., Allen, J. P., Cheung, B. H., Chiu, C.-H. & Hauser, A. R. Systemic infection facilitates transmission of Pseudomonas aeruginosa in mice. *Nat. Commun.* **11**, 543 (2020). 10.1038/s41467-020-14363-4 PMC6987207 31992714

[50] Lebrun-Corbin, M. et al. Pseudomonas aeruginosa population dynamics in a vancomycin-induced murine model of gastrointestinal carriage. *mBio* **16**, e0313624 (2025). 10.1128/mbio.03136-24 PMC12077156 40207916

[51] Campbell, I. W., Hullahalli, K., Turner, J. R. & Waldor, M. K. Quantitative dose-response analysis untangles host bottlenecks to enteric infection. *Nat. Commun.* **14**, 456 (2023). 10.1038/s41467-023-36162-3 PMC9884216 36709326

[52] Louie, A., Zhang, T., Becattini, S., Waldor, M. K. & Portnoy, D. A. A multiorgan trafficking circuit provides purifying selection of listeria monocytogenes virulence genes. *mBio* **10**, e02948–19 (2019). 10.1128/mBio.02948-19 PMC6918090 31848289

[53] Zhang, T. et al. Deciphering the landscape of host barriers to Listeria monocytogenes infection. *Proc. Natl. Acad. Sci. Usa.* **114**, 6334–6339 (2017). 10.1073/pnas.1702077114 PMC5474794 28559314

[54] Chevée, V. et al. Temporal and spatial dynamics of Listeria monocytogenes central nervous system infection in mice. *Proc. Natl. Acad. Sci. USA* **121**, e2320311121 (2024). 10.1073/pnas.2320311121 PMC11046682 38635627

[55] Hotinger, J. A., Campbell, I. W., Hullahalli, K., Osaki, A. & Waldor, M. K. Quantification of Salmonella enterica serovar Typhimurium population dynamics in murine infection using a highly diverse barcoded library. *eLife* **13**, RP101388 (2025). 10.7554/eLife.101388 PMC11825126 39945742

[56] Holmes, C. L. et al. Patterns of Klebsiella pneumoniae bacteremic dissemination from the lung. *Nat. Commun.* **16**, 785 (2025). 10.1038/s41467-025-56095-3 PMC11742683 39824859

[57] Peterson, S. T. et al. TNF signaling maintains local restriction of bacterial founder populations in intestinal and systemic sites during oral Yersinia infection. *mBio* **16**, e0177925 (2025). 10.1128/mbio.01779-25 PMC12506003 40923791

[58] Wincott, C. J. et al. Cellular barcoding of protozoan pathogens reveals the within-host population dynamics of Toxoplasma gondii host colonization. *Cell Rep. Methods* **2**, 100274 (2022). 10.1016/j.crmeth.2022.100274 PMC9421581 36046624

[59] Gotelli, N. J. & Ulrich, W. Statistical challenges in null model analysis. *Oikos* **121**, 171–180 (2012).

[60] Sacks, D. L. & Melby, P. C. Animal Models for the Analysis of Immune Responses to Leishmaniasis. *Curr. Protoc. Immunol*. **108**, 19.2.1–19.2.24 (2015). 10.1002/0471142735.im1902s108 25640990

[61] Shannon, C. E. et al. A Mathematical Theory of Communication. *Bell Syst. Tech. J.* **27**, 379–423 (1948).

[62] Hill, M. O. et al. Diversity and evenness: a unifying notation and its consequences. *Ecology* **54**, 427–432 (1973).

[63] Spellerberg, I. F. & Fedor, P. J. A tribute to Claude Shannon (1916–2001) and a plea for more rigorous use of species richness, species diversity and the ‘Shannon–Wiener’ Index. *Glob. Ecol. Biogeogr.* **12**, 177–179 (2003).

[64] Doehl, J. S. P. et al. Spatial point pattern analysis identifies mechanisms shaping the skin parasite landscape in leishmania donovani infection. *Front. Immunol.* **12**, 795554 (2021). 10.3389/fimmu.2021.795554 PMC8716623 34975901

[65] Melo, G. D. et al. New insights into experimental visceral leishmaniasis: Real-time in vivo imaging of Leishmania donovani virulence. *PLoS Negl. Trop. Dis.* **11**, e0005924 (2017). 10.1371/journal.pntd.0005924 PMC5629011 28945751

[66] Ward, A. I. et al. In Vivo Analysis of Trypanosoma cruzi Persistence Foci at Single-Cell Resolution. *mBio* **11**, e01242–20 (2020). 10.1128/mBio.01242-20 PMC7407085 32753495

[67] Beamer, G., Major, S., Das, B. & Campos-Neto, A. Bone marrow mesenchymal stem cells provide an antibiotic-protective niche for persistent viable Mycobacterium tuberculosis that survive antibiotic treatment. *Am. J. Pathol.* **184**, 3170–3175 (2014). 10.1016/j.ajpath.2014.08.024 PMC4261085 25451154

[68] Liu, F.-F. & Li, K. Malaria and dyserythropoiesis: a mini review. *Front. Cell. Infect. Microbiol.* **15**, 1679337 (2025). 10.3389/fcimb.2025.1679337 PMC12446233 40980010

[69] Waugh, M. C. et al. Clinical anemia predicts dermal parasitism and reservoir infectiousness during progressive visceral leishmaniosis. *PLoS Negl. Trop. Dis.* **18**, e0012363 (2024). 10.1371/journal.pntd.0012363 PMC11578447 39514579

[70] Kirstein, O. D. et al. Minimally invasive microbiopsies: a novel sampling method for identifying asymptomatic, potentially infectious carriers of Leishmania donovani. *Int. J. Parasitol.* **47**, 609–616 (2017). 10.1016/j.ijpara.2017.02.005 PMC5596977 28455239

[71] Romano, A. et al. Interferon-γ-producing CD4+ T cells drive monocyte activation in the bone marrow during experimental leishmania donovani infection. *Front. Immunol.* **12**, 700501 (2021). 10.3389/fimmu.2021.700501 PMC8453021 34557190

[72] Forrester, S. et al. Tissue specific dual RNA-Seq defines host-parasite interplay in murine visceral leishmaniasis caused by Leishmania donovani and Leishmania infantum. *Microbiol. Spectr.* **10**, e0067922 (2022). 10.1128/spectrum.00679-22 PMC9045295 35384718

[73] Glennie, N. D., Volk, S. W. & Scott, P. Skin-resident CD4+ T cells protect against Leishmania major by recruiting and activating inflammatory monocytes. *PLoS Pathog.* **13**, e1006349 (2017). 10.1371/journal.ppat.1006349 PMC5409171 28419151

[74] Glennie, N. D. et al. Skin-resident memory CD4+ T cells enhance protection against *Leishmania major* infection. *J. Exp. Med.* **212**, 1405–1414 (2015). 10.1084/jem.20142101 PMC4548053 26216123

[75] Furman, D. et al. Chronic inflammation in the etiology of disease across the life span. *Nat. Med.* **25**, 1822–1832 (2019). 10.1038/s41591-019-0675-0 PMC7147972 31806905

[76] Ong, H. B., Clare, S., Roberts, A. J., Wilson, M. E. & Wright, G. J. Establishment, optimisation and quantitation of a bioluminescent murine infection model of visceral leishmaniasis for systematic vaccine screening. *Sci. Rep.* **10**, 4689 (2020). 10.1038/s41598-020-61662-3 PMC7070049 32170135

[77] Delignette-Muller, M. L. & Dutang, C. fitdistrplus: an *R* package for fitting distributions. *J. Stat. Softw*. **64**, 1–34 (2015).

[78] Loughrey, C. CiaraLoughrey-scientist/barseq-Leishmania-2025: June 2026 release. *Zenodo* 10.5281/ZENODO.20843226 (2026).

[79] Carnielli, J. B. T. et al. 3’Nucleotidase/nuclease is required for Leishmania infantum clinical isolate susceptibility to miltefosine. *EBioMedicine* **86**, 104378 (2022). 10.1016/j.ebiom.2022.104378 PMC9713291 36462405

[80] Soysa, R., Tran, K. D., Ullman, B. & Yates, P. A. Integrating ribosomal promoter vectors that offer a choice of constitutive expression profiles in Leishmania donovani. *Mol. Biochem. Parasitol.* **204**, 89–92 (2015). 10.1016/j.molbiopara.2016.01.008 PMC4794367 26844641

[81] Branchini, B. R. et al. Red-emitting luciferases for bioluminescence reporter and imaging applications. *Anal. Biochem.* **396**, 290–297 (2010). 10.1016/j.ab.2009.09.009 19748472

[82] Shaner, N. C. et al. Improved monomeric red, orange and yellow fluorescent proteins derived from Discosoma sp. red fluorescent protein. *Nat. Biotechnol.* **22**, 1567–1572 (2004). 10.1038/nbt1037 15558047

[83] Beattie, L. et al. Dynamic imaging of experimental Leishmania donovani-induced hepatic granulomas detects Kupffer cell-restricted antigen presentation to antigen-specific CD8 T cells. *PLoS Pathog.* **6**, e1000805 (2010). 10.1371/journal.ppat.1000805 PMC2837408 20300603

[84] Misslitz, A., Mottram, J. C., Overath, P. & Aebischer, T. Targeted integration into a rRNA locus results in uniform and high level expression of transgenes in Leishmania amastigotes. *Mol. Biochem. Parasitol.* **107**, 251–261 (2000). 10.1016/s0166-6851(00)00195-x 10779601

[85] Martel, D., Beneke, T., Gluenz, E., Späth, G. F. & Rachidi, N. Characterisation of Casein Kinase 1.1 in *Leishmania donovani* Using the CRISPR Cas9 Toolkit. *BioMed. Res. Int.* **2017**, 1–11 (2017). 10.1155/2017/4635605 PMC5733176 29333442

[86] Beneke, T. et al. A CRISPR Cas9 high-throughput genome editing toolkit for kinetoplastids. *R. Soc. Open Sci.* **4**, 170095 (2017). 10.1098/rsos.170095 PMC5451818 28573017

[87] Beneke, T. & Gluenz, E. Bar-seq strategies for the LeishGEdit toolbox. *Mol. Biochem. Parasitol.* **239**, 111295 (2020). 10.1016/j.molbiopara.2020.111295 32659298

[88] Droop, A. uoy-research/fqbarcode: Initial release. *Zenodo* 10.5281/ZENODO.20843221 (2026).

[89] Loughrey, C. Bottling it all up: using parasite population dynamics to understand dissemination and identify susceptibility pathways in visceral leishmaniasis. PhD Thesis, University of York https://etheses.whiterose.ac.uk/id/eprint/36199/ (2024).

[90] Oksanen, J., et al. vegan: Community Ecology Package (version 2.8-0) [Software]. https://vegandevs.github.io/vegan/ (2025).

[91] Csardi, G. & Nepusz, T. The igraph software package for complex network research. *InterJ.**Complex Syst.* **1695**, 1–9 (2006).

[92] Csárdi, G. et al. igraph for R: R interface of the igraph library for graph theory and network analysis. (version 2.1.4) [Software] *Zenodo* 10.5281/ZENODO.7682609 (2025).

[93] Pedersen, T. L. ggraph: An implementation of grammar of graphics for graphs and networks. (version 2.2.1) [Software] https://ggraph.data-imaginist.com (2025).

[94] Hao, Y. et al. Integrated analysis of multimodal single-cell data. *Cell* **184**, 3573–3587.e29 (2021). 10.1016/j.cell.2021.04.048 PMC8238499 34062119

[95] Hao, Y. et al. Dictionary learning for integrative, multimodal and scalable single-cell analysis. *Nat. Biotechnol.* **42**, 293–304 (2024). 10.1038/s41587-023-01767-y PMC10928517 37231261

[96] Stuart, T. et al. Comprehensive integration of single-cell data. *Cell* **177**, 1888–1902.e21 (2019). 10.1016/j.cell.2019.05.031 PMC6687398 31178118

[97] Butler, A., Hoffman, P., Smibert, P., Papalexi, E. & Satija, R. Integrating single-cell transcriptomic data across different conditions, technologies, and species. *Nat. Biotechnol.* **36**, 411–420 (2018). 10.1038/nbt.4096 PMC6700744 29608179

[98] Satija, R., Farrell, J. A., Gennert, D., Schier, A. F. & Regev, A. Spatial reconstruction of single-cell gene expression data. *Nat. Biotechnol.* **33**, 495–502 (2015). 10.1038/nbt.3192 PMC4430369 25867923

[99] Wickham, H. et al. Welcome to the Tidyverse. *J. Open Source Softw.* **4**, 1686 (2019).

[100] Alboukadel, K. ggpubr: ‘ggplot2’ Based Publication Ready Plots (version 0.6.1) [Software]. https://rpkgs.datanovia.com/ggpubr/ (2025).

[101] Tierney, N. & Cook, D. Expanding tidy data principles to facilitate missing data exploration, visualization and assessment of imputations. *J. Stat. Softw*. **105**, 1–31 (2023).

[102] Alboukadel, K. rstatix: Pipe-friendly framework for basic statistical tests (version 0.7.2) [Software]. https://rpkgs.datanovia.com/rstatix/ (2023).

[103] Zhang, Y. Likelihood-based and Bayesian methods for Tweedie compound Poisson linear mixed models. *Stat. Comput.* **23**, 743–757 (2013).

[104] Dunn, P. K. Tweedie: Evaluation of Tweedie Exponential Family Models. (version 2.3.5) [Software] https://www.rdocumentation.org/packages/tweedie/versions/2.3.5 (2022).

[105] Dunn, P. K. & Smyth, G. K. Evaluation of Tweedie exponential dispersion model densities by Fourier inversion. *Stat. Comput.* **18**, 73–86 (2008).

[106] Dunn, P. K. & Smyth, G. K. Series evaluation of Tweedie exponential dispersion model densities. *Stat. Comput.* **15**, 267–280 (2005).

[107] Frees, E. W., Meyers, G. & Cummings, A. D. Summarizing insurance scores using a Gini index. *J. Am. Stat. Assoc.* **106**, 1085–1098 (2011).

