# Supplementary material for: Bi-directional highways and super-seeder tissues underpin parasite dissemination in experimental Leishmania donovani infection
Source: Nat Commun. 2026 Aug 26;17:10194. doi: 10.1038/s41467-026-77072-4 (PMC13612394; doi:10.1038/s41467-026-77072-4)
Supplement: Supplementary file 1 [file 41467_2026_77072_MOESM1_ESM.pdf]

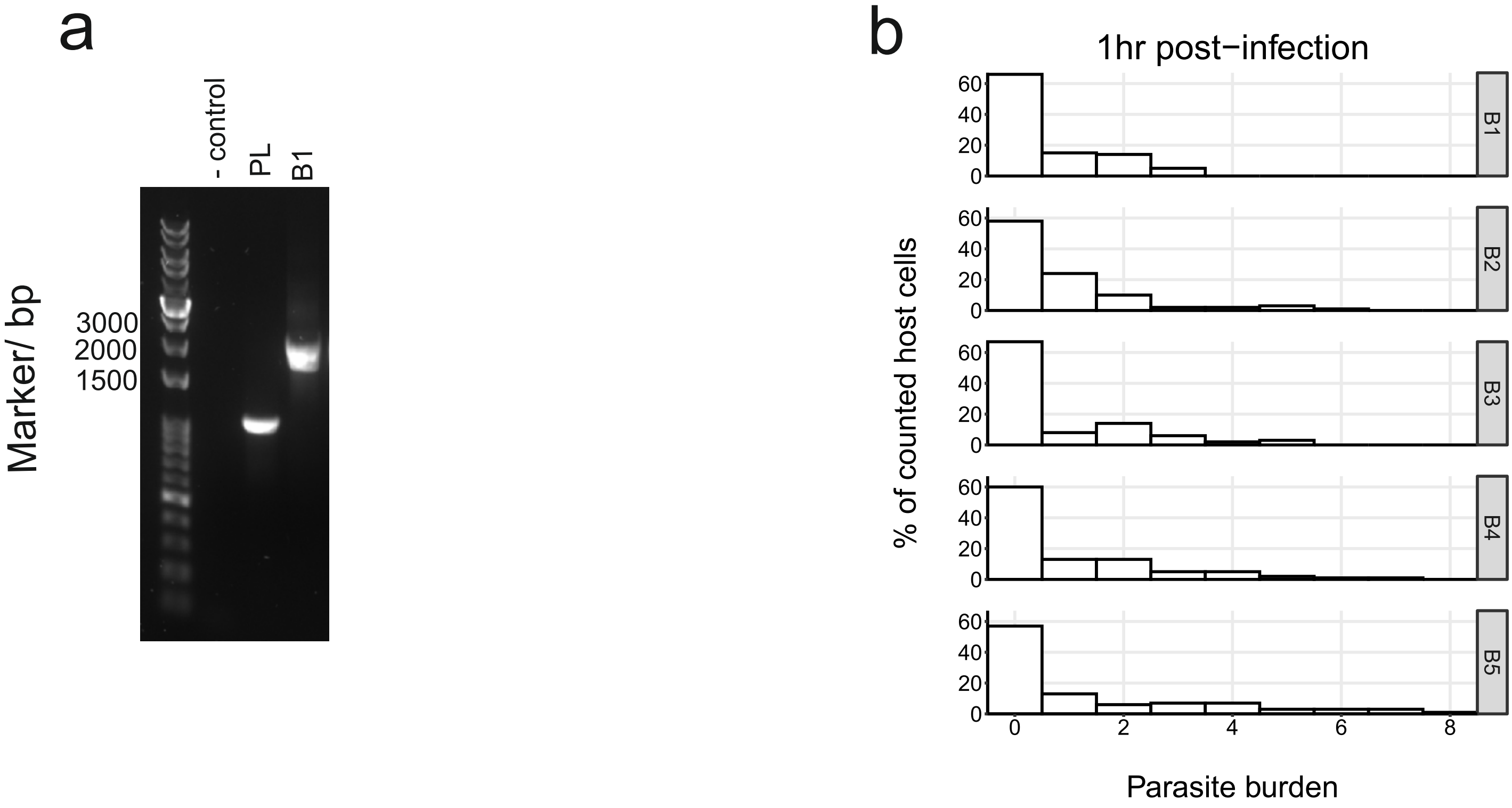


**Supplementary Figure 1: Barcoded parasite validation. a,** Validation PCR for barcode insertion in *L. donovani*. PL = parental line with HYG present. B1 = mutant parasites containing barcode 1 (B1) and BSD selection marker. b, Histograms of in vitro parasite infection burdens after a 1 hr incubation of promastigote parasites with murine bone marrow derived macrophages for five individual barcoded parasite lines, here termed B1-5. Source data are provided as a Source Data file.

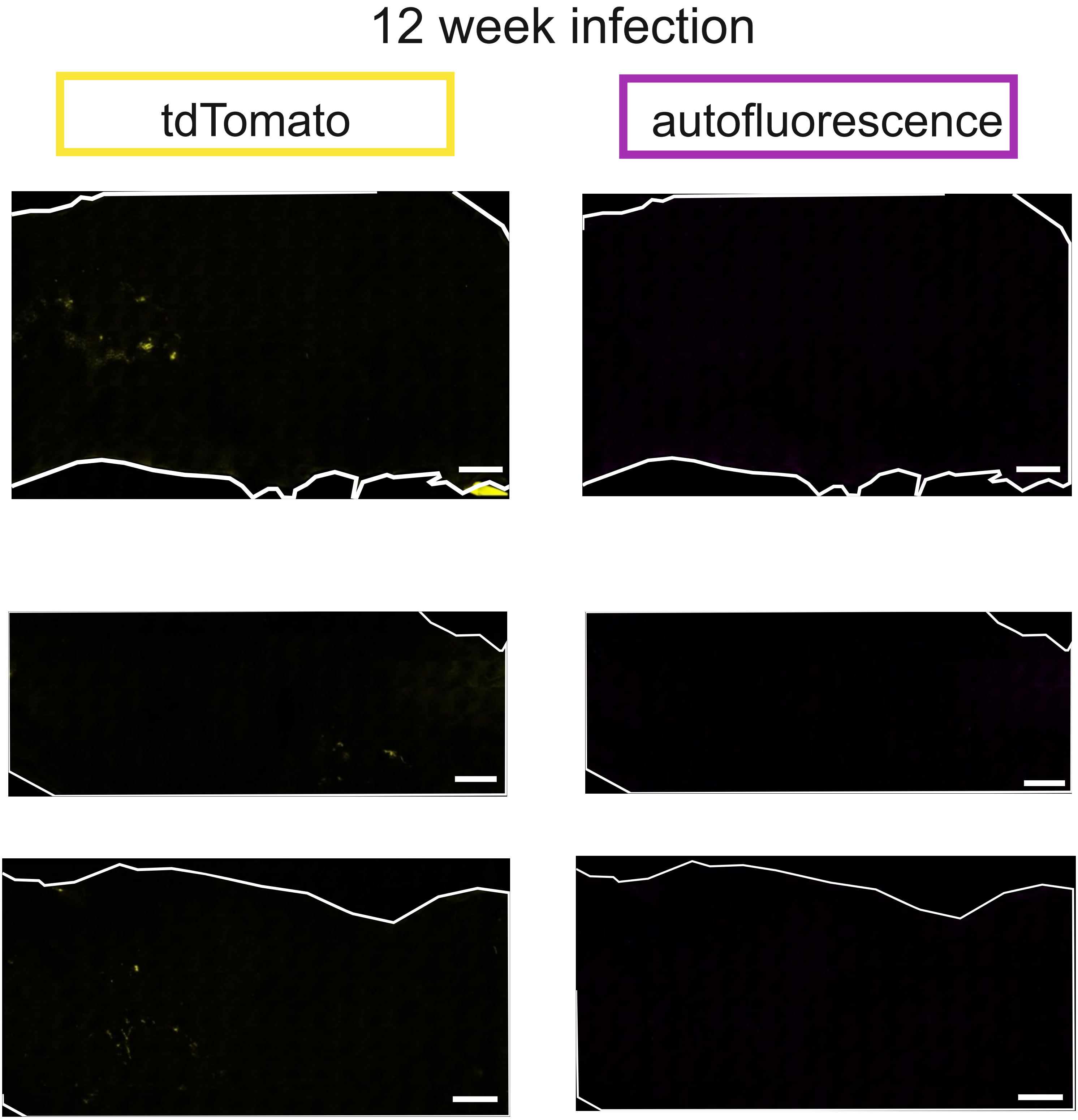


**Supplementary Figure 2: Detection of tdTomato-expressing *L. donovani* in the skin of C57BL/6J mice.** Whole skin images (after stitching of panels) of infected skin at 12 weeks post-infection (n=3 female mice). tdTomato signal shown in yellow. Autofluorescence signal shown in magenta. Scale bar represents 5000µm.

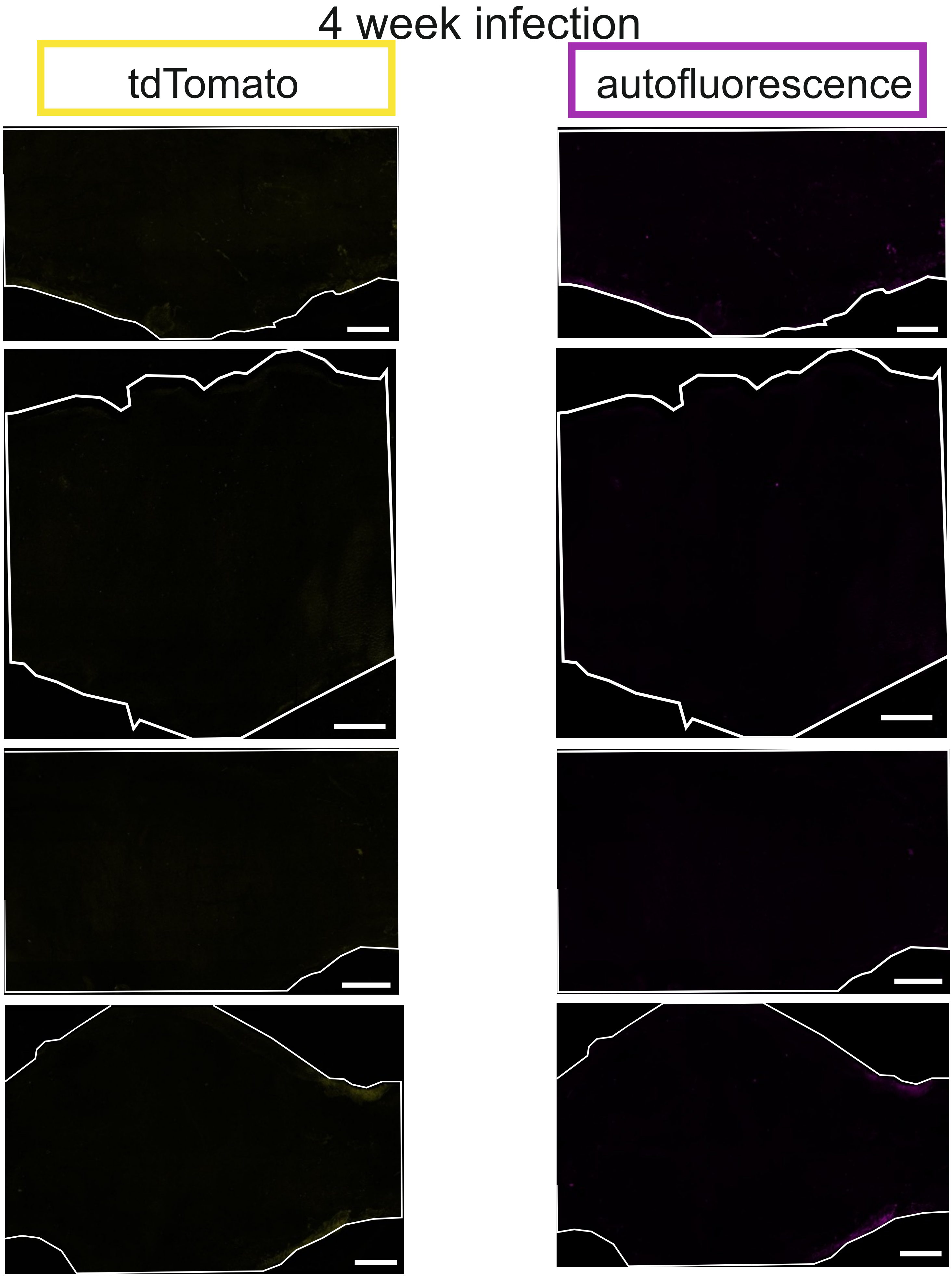


**Supplementary Figure 3: Detection of tdTomato-expressing *L. donovani* in the skin of C57BL/6J mice at 4 weeks post-infection.** Whole skin images (after stitching of panels) of infected skin at 4 weeks post-infection (n=4 female mice). tdTomato signal shown in yellow. Autofluorescence signal shown in magenta. Scale bar represents 5000µm.

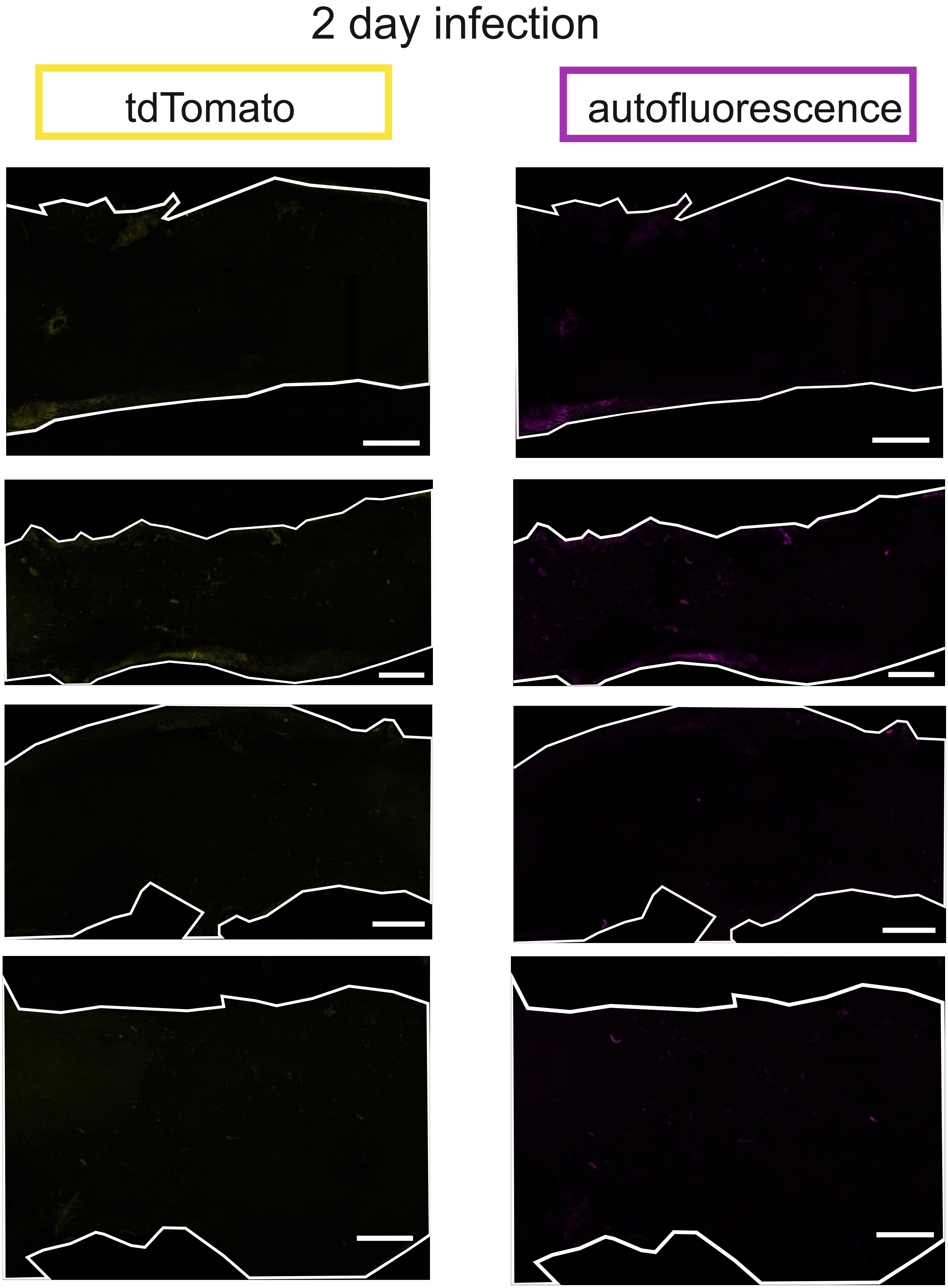


**Supplementary Figure 4: Detection of tdTomato-expressing *L. donovani* in the skin of C57BL/6J mice at 2 days post-infection.** Whole skin images (after stitching of panels) of infected skin at 2 days post-infection (n=4 female mice). tdTomato signal shown in yellow. Autofluorescence signal shown in magenta. Scale bar represents 5000µm.

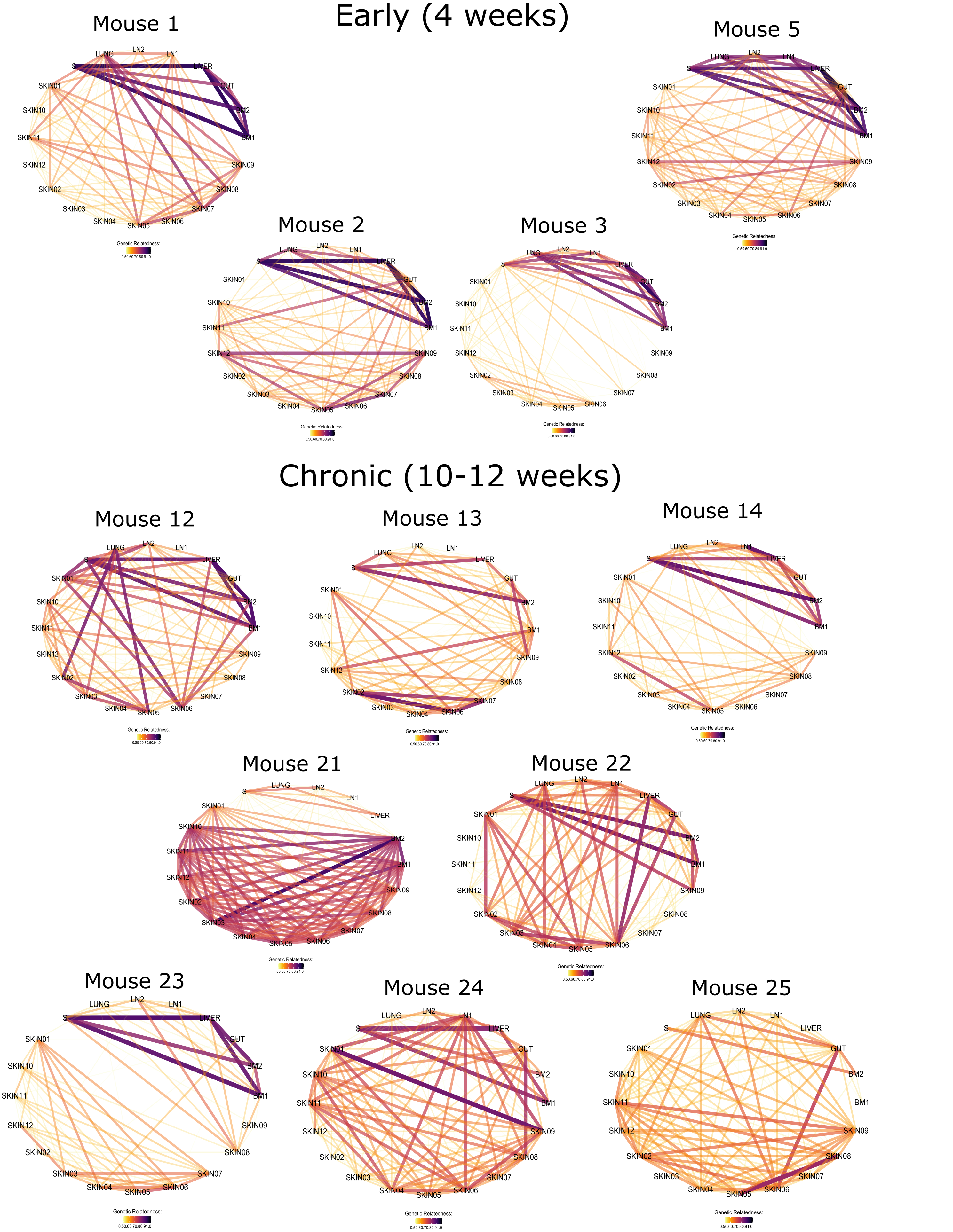


**Supplementary Figure 5: Tissue parasite population connectedness networks.** Network representations of Genetic Distance (GD; shown as edges) between parasite populations in each tissue (vertices). Networks for all mice at 4 weeks (early) and 10-12 weeks (chronic) post-infection, with all GD above 0.5 removed. Line colour indicates genetic relatedness (1-GD) as shown in the legends for each network. Thicker, more opaque connecting lines indicate a higher relatedness (lower GD). Source data are provided as a Source Data file. LN = Lymph Node; BM = Bone Marrow

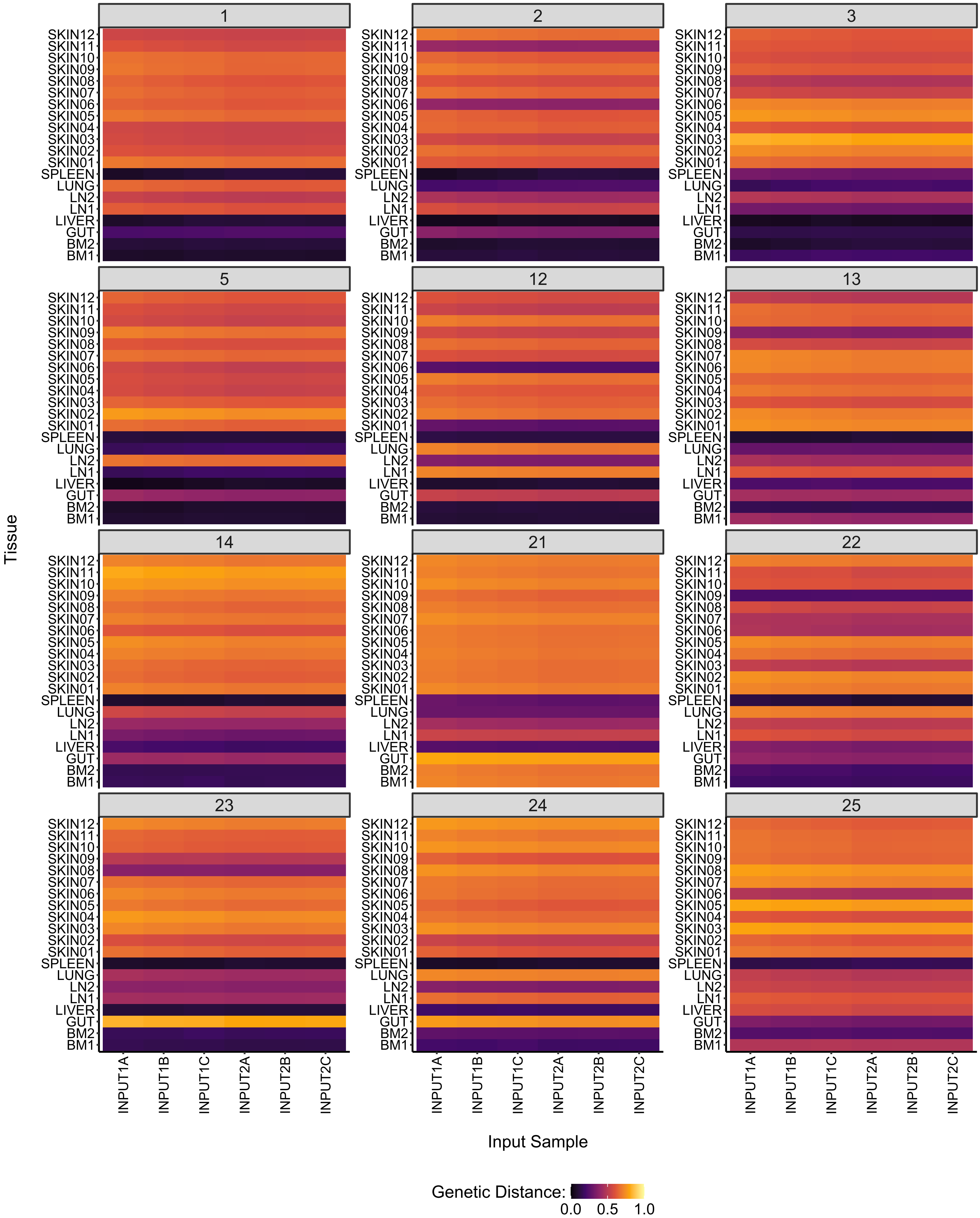


**Supplementary Figure 6: Tissue parasite population relatedness to input.** Genetic Distance (GD) of parasite populations to input pool (n=6 sequencing repeats from a single culture) in all tissues for all mice at 4 weeks (early) and 10-12 weeks (chronic) post-infection. Strip text identifies mouse ID. Colour scale applies to all heatmaps. Source data are provided as a Source Data file. LN = Lymph Node; BM = Bone Marrow

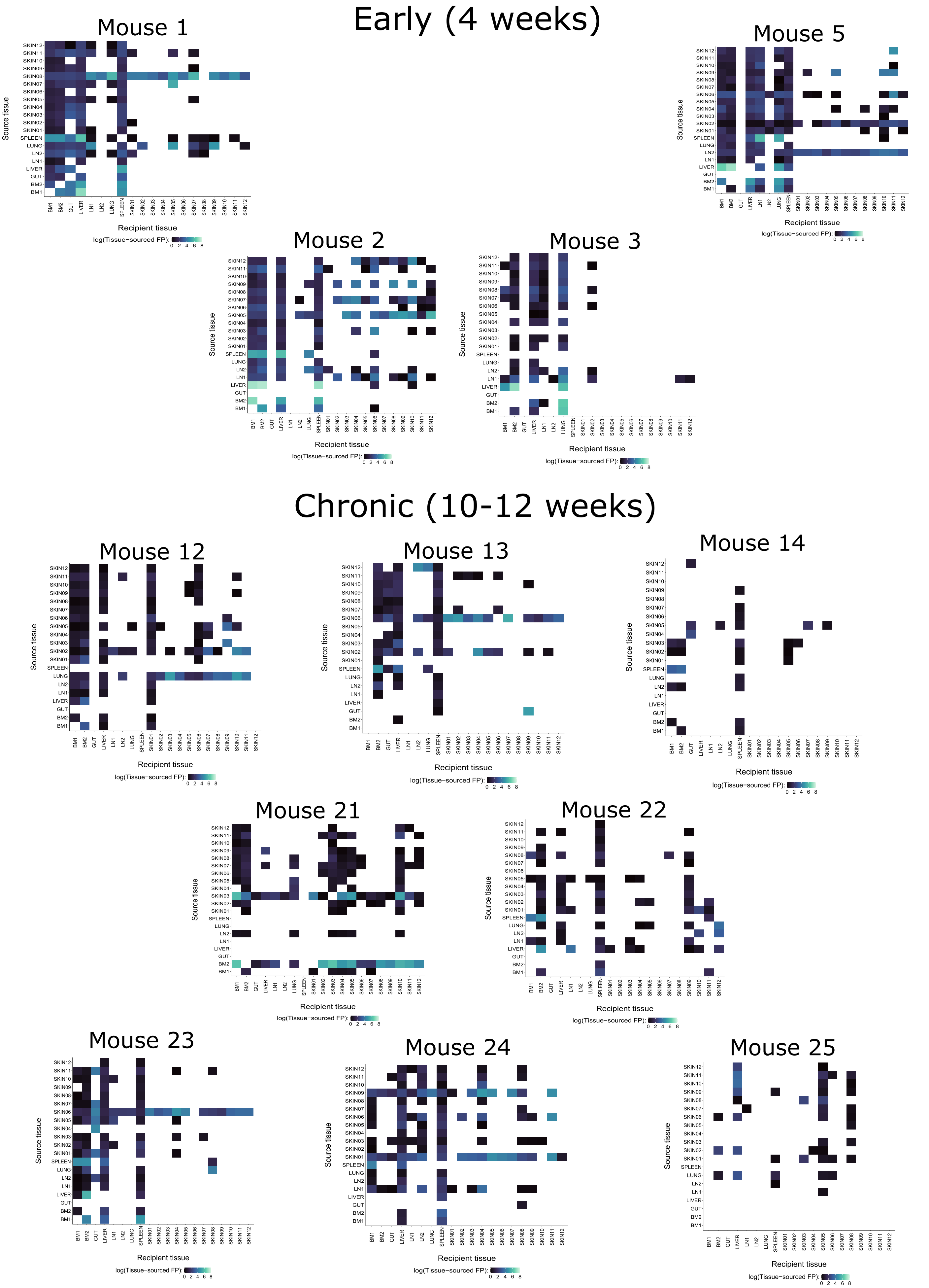


**Supplementary Figure 7: Tissue to tissue parasite seeding heatmaps.** Heatmaps for Tissue Sourced Founder Population (TSFP) for all mice at 4 weeks (early) and 10-12 weeks (chronic) post-infection. Scale represents log(TSFP) for each directional tissue to tissue relationship. White indicates those TSFP values < 1 or tissues which were excluded in filtering. No further thresholding was applied to TSFP for the heatmaps shown. Source data are provided as a Source Data file. LN = Lymph Node; BM = Bone Marrow

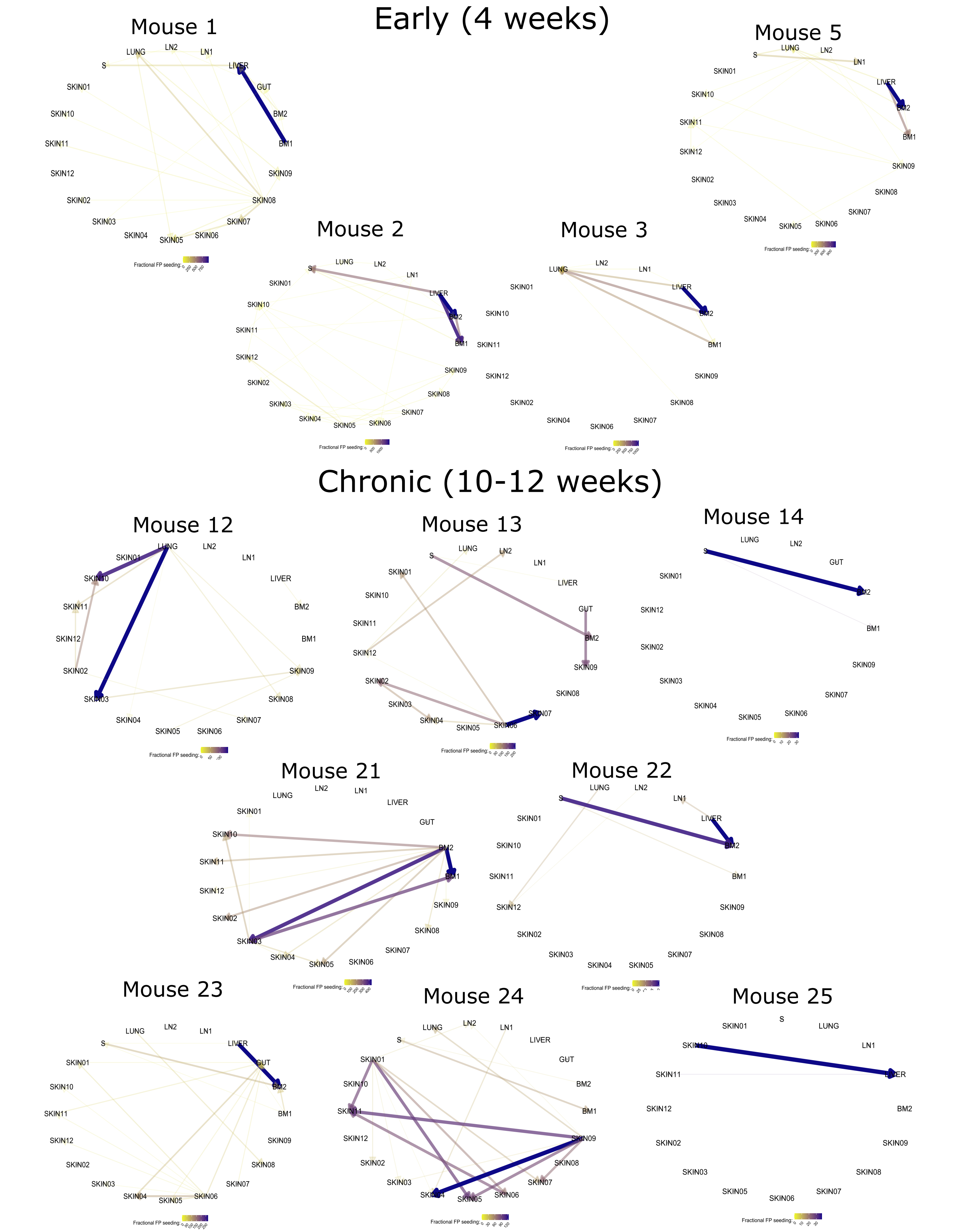


**Supplementary Figure 8: Tissue to tissue parasite seeding networks.** Full networks constructed using Fractional Founder Population (FFP) as the metric for edge weight for all mice at 4 weeks (early) and 10-12 weeks (chronic) post-infection. Line colour indicates FFP as in legends. Thicker, more opaque lines indicate a higher FFP in the direction indicated by the arrowhead. A threshold of FFP > 20 was applied for network creation. Source data are provided as a Source Data file. LN = Lymph Node; BM = Bone Marrow

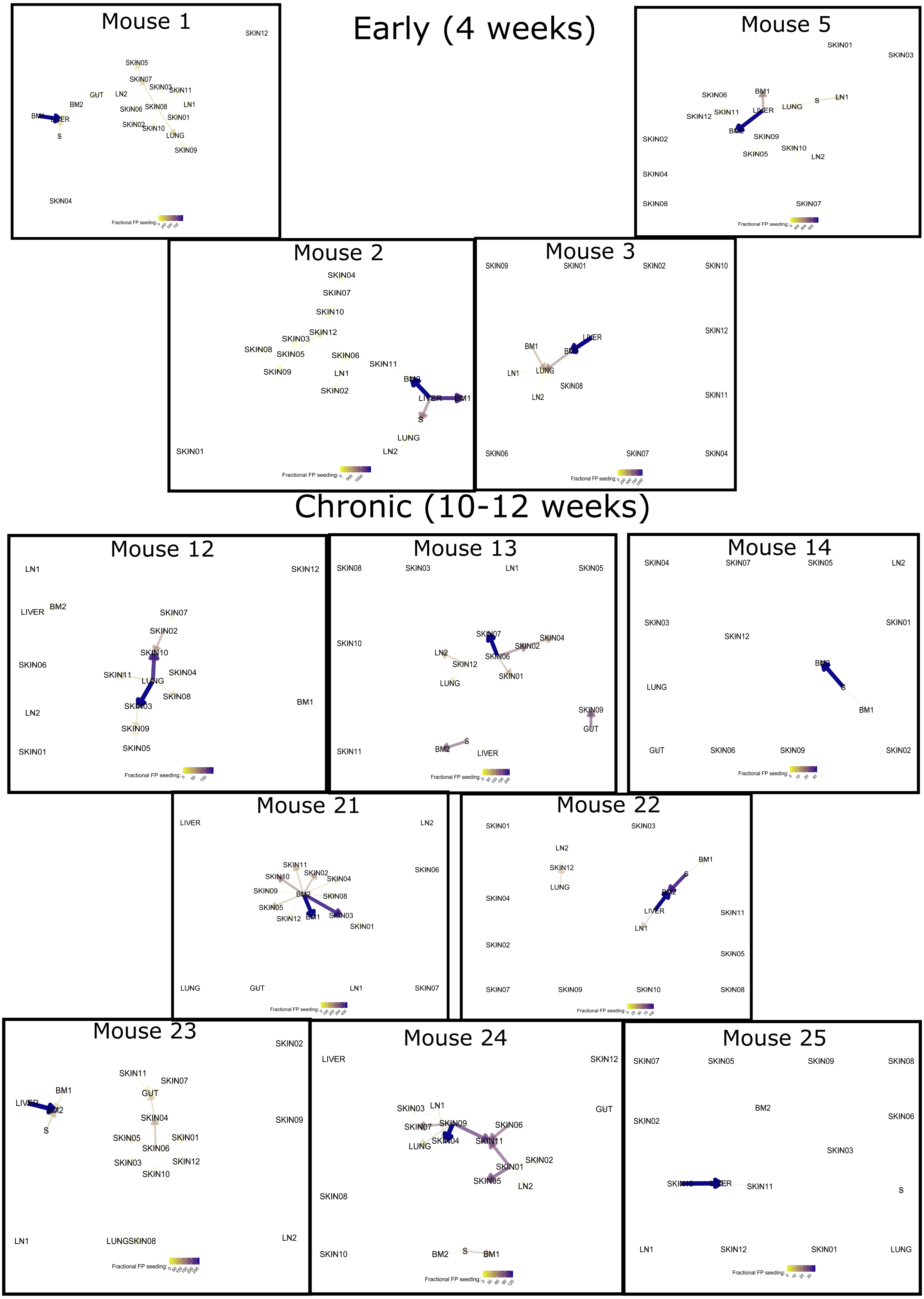


**Supplementary Figure 9: In vivo parasite dissemination highways.** Minimum spanning tree (MST) network representations of Fractional Founder Population (FFP) for all mice at 4 weeks (early) and 10-12 weeks (chronic) post-infection. Legends, line colour and thickness indicate FFP. Arrowheads represent direction of FFP. A threshold of FFP > 20 was applied for initial network creation, prior to MST computation. Source data are provided as a Source Data file. LN = Lymph Node; BM = Bone Marrow

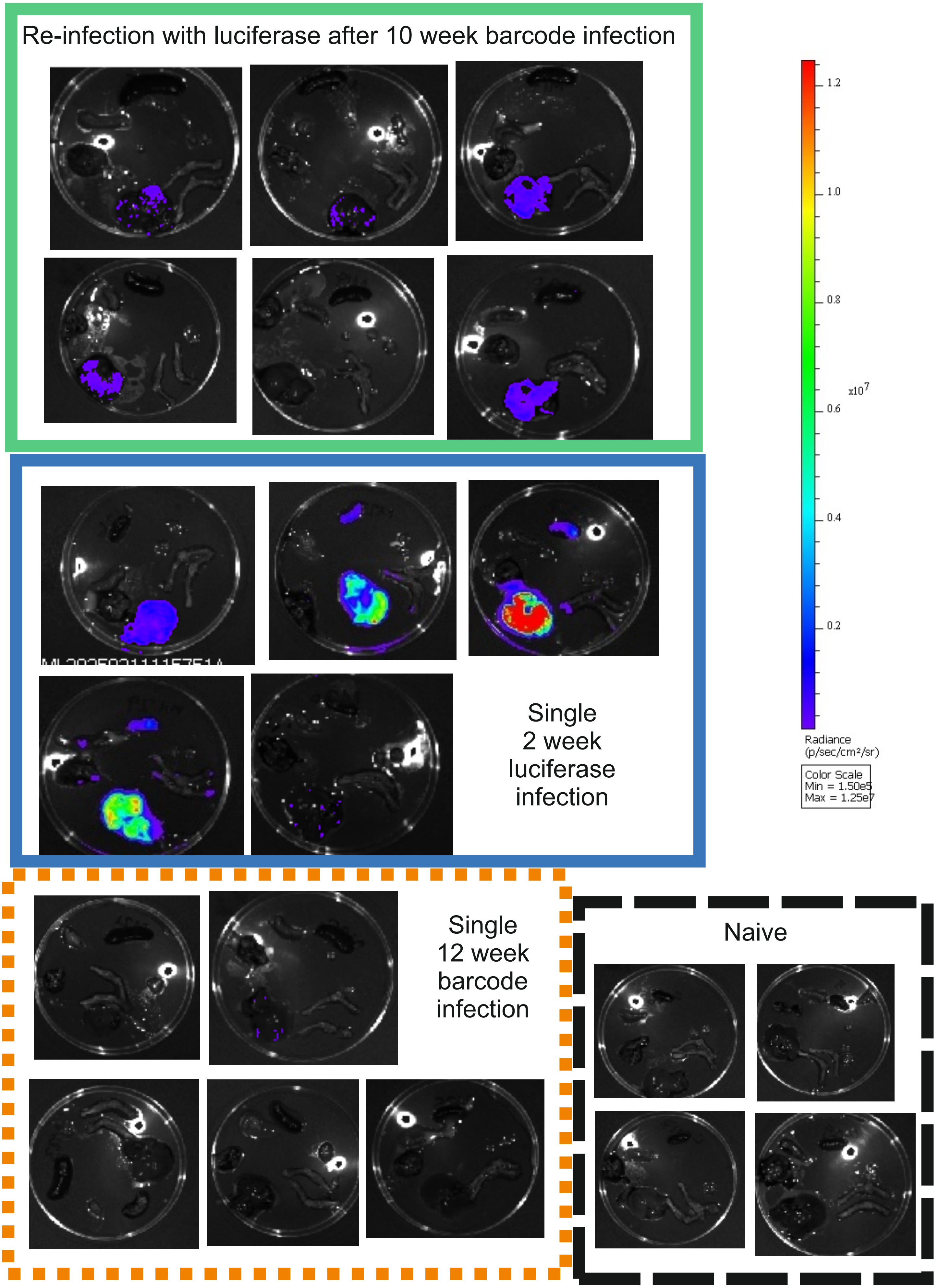


**Supplementary Figure 10: Tissue parasite burdens ex vivo.** Ex vivo IVIS imaging of tissues from each individual mouse, with infection group as indicated by the box label. Radiance scale (p/sec/cm$^2$/sr) applies to all images. Source data are provided as a Source Data file.

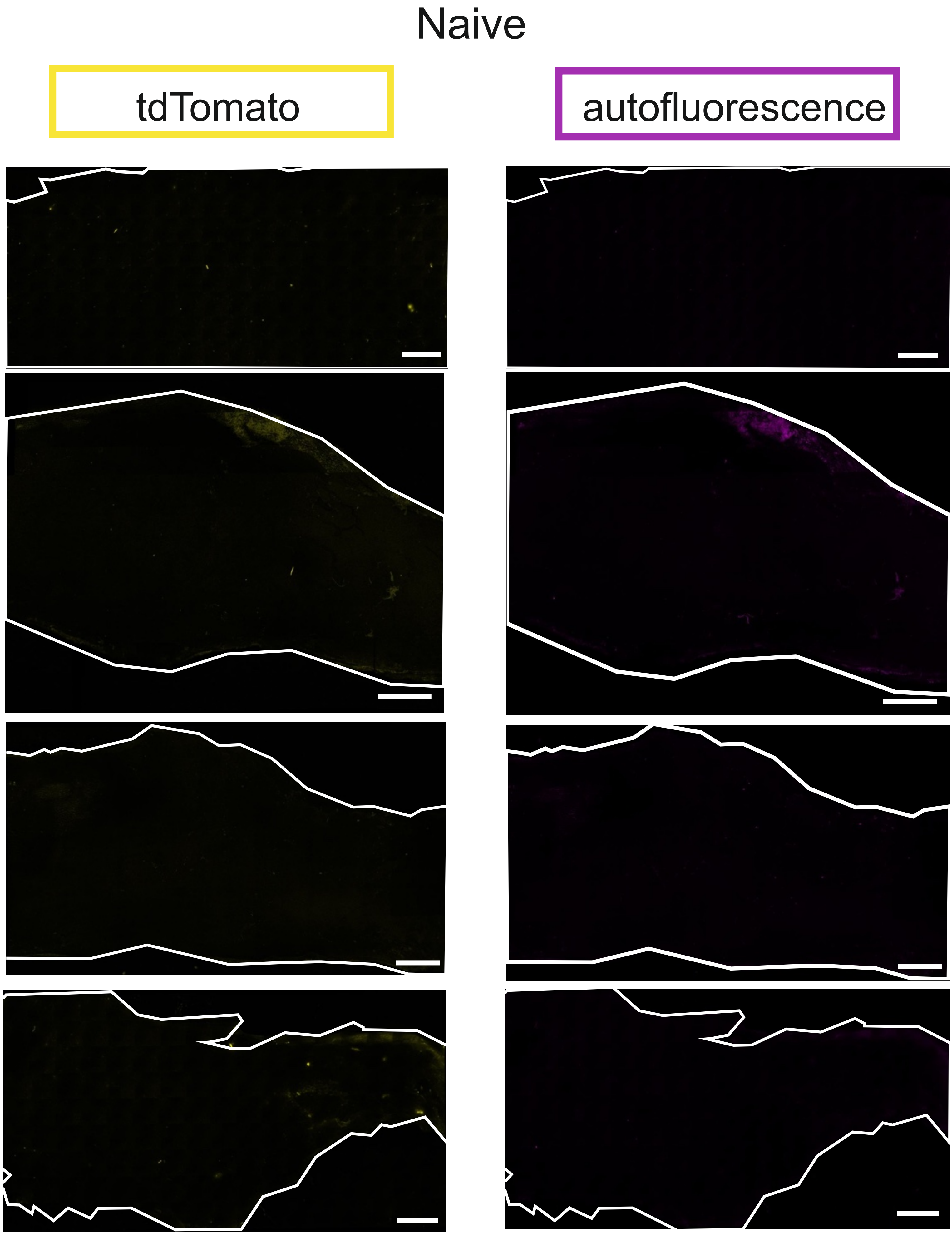


**Supplementary Figure 11: Detection of autofluorescence signal in the skin of naïve C57BL/6J mice.** Whole skin images (after stitching of panels) of naïve mouse skin. tdTomato signal spectrum background shown in yellow. Autofluorescence signal shown in magenta. Scale bar represents 5000µm.

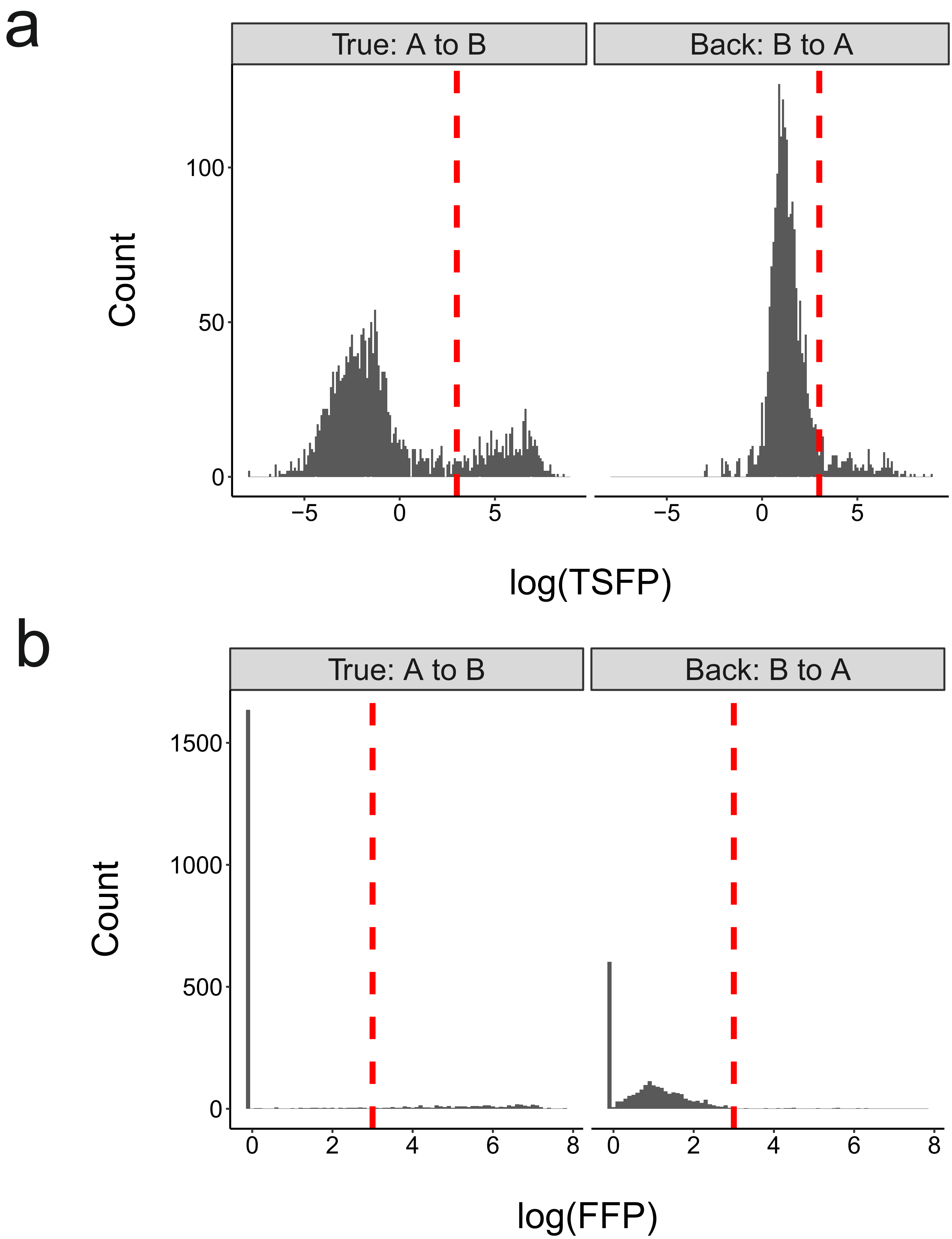


**Supplementary Figure 12: Thresholding histograms for true and back migration between INPUT and samples. a, b,** Histograms for Tissue Sourced Founder Population (TSFP) (a) and Fractional Founder Population (FFP) (b) for true migration (INPUT samples as population A, tissue samples as population B) and back migration (tissue samples as population A, INPUT samples as population B). Red dotted line indicates the threshold applied, based on distribution shifts. All FFP below 1 were changed to 0.9 for visualisation. All TSFP of negative value or NA were changed to 0 for visualisation. Source data are provided as a Source Data file.

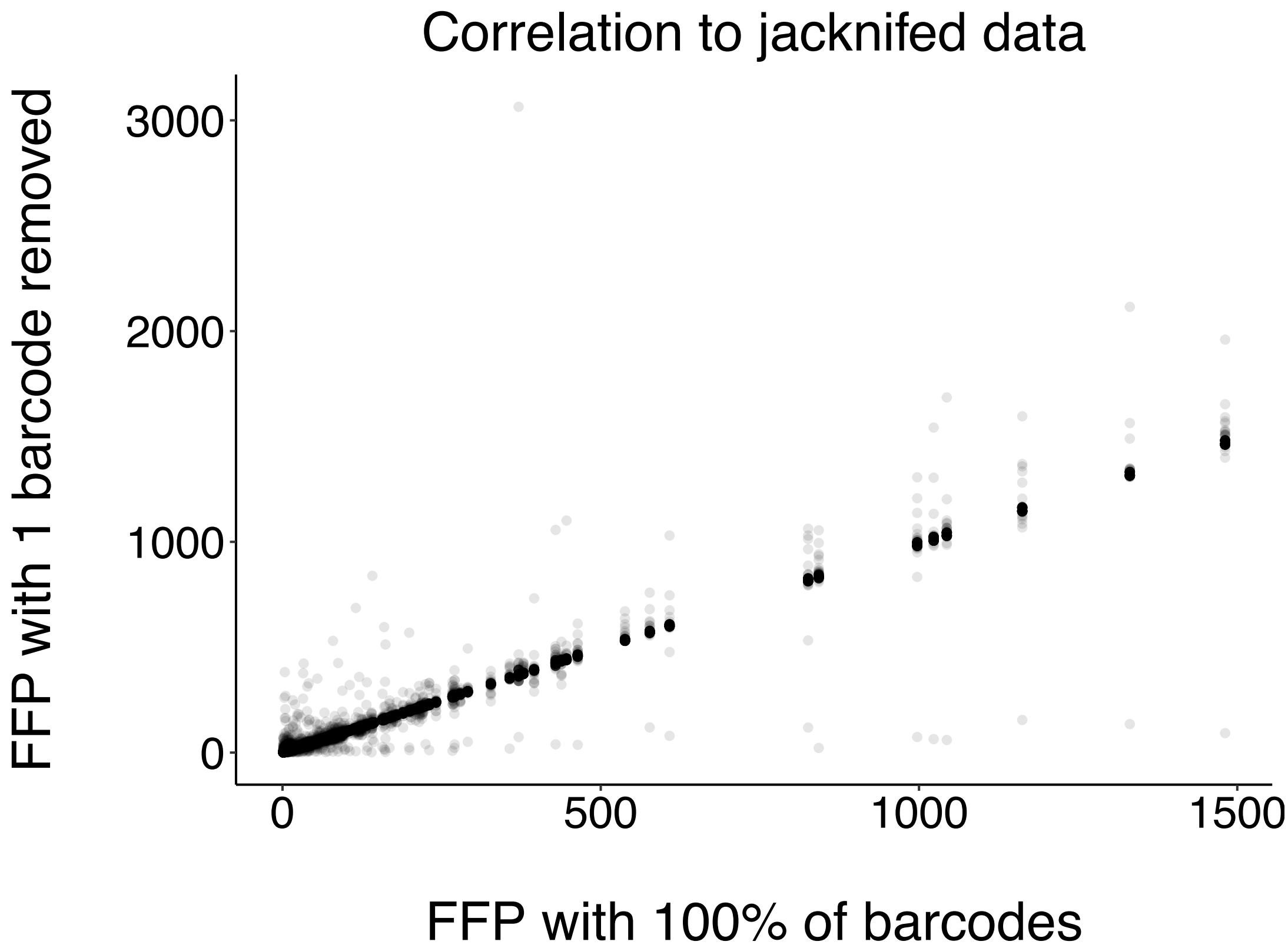


**Supplementary Figure 13: Correlation of Fractional Founder Population (FFP) after jack-knifing.** Dot plot showing the correlation of FFP calculated using all barcodes to FFP calculated using the same data with each barcode iteratively removed before calculation for all pairwise sample comparisons. Each point represents an individual tissue pair. All FFPs of less than 1 or NA were changed to 0 before analysis and visualisation. Source data are provided as a Source Data file

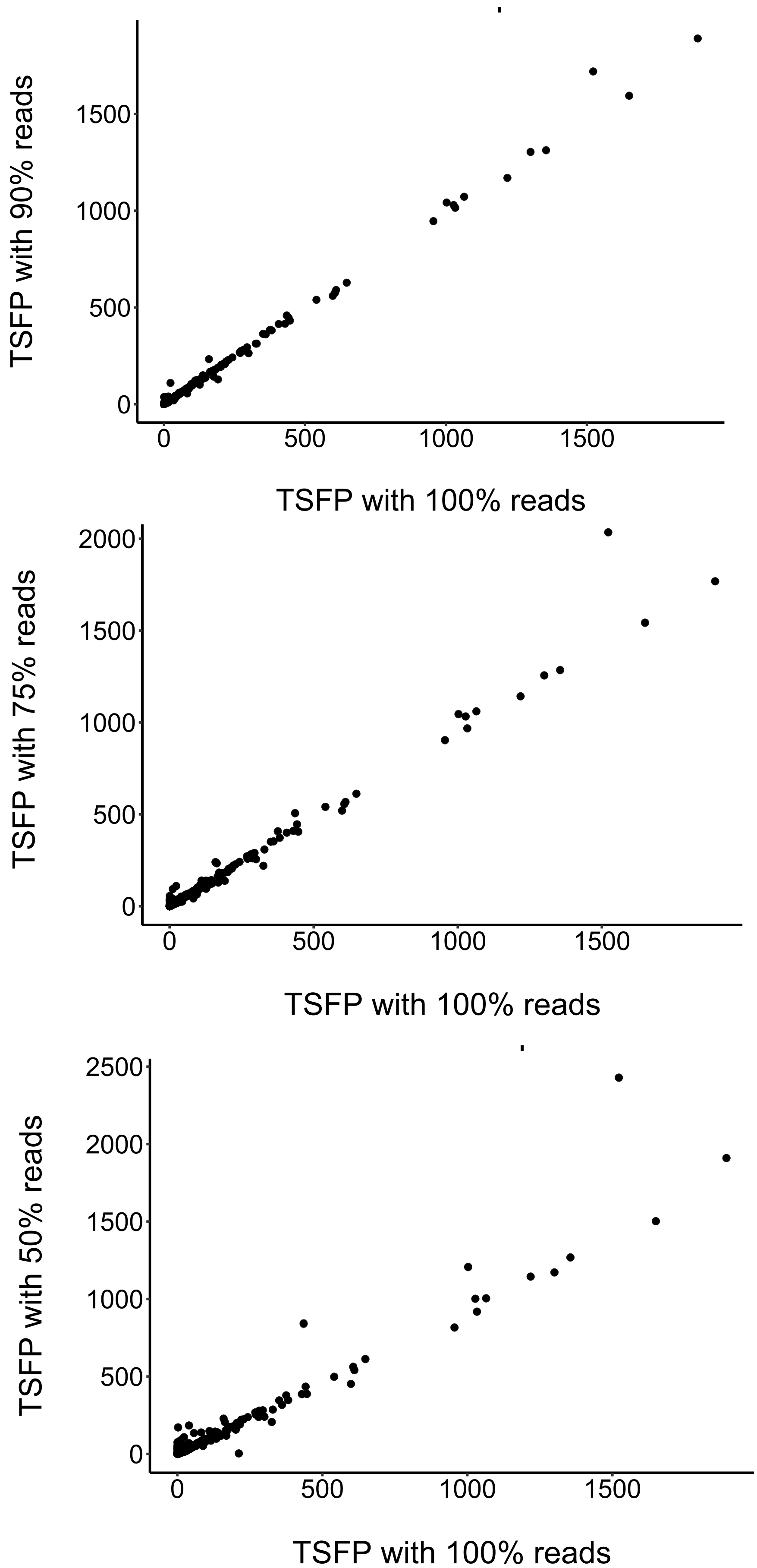


**Supplementary Figure 14: Correlation of Tissue Sourced Founder Population (TSFP) calculated with reduced sequence read depth.** TSFP calculations for tissue pairs calculated with 100% read depth and with a reduced read depth of a) 90%, b) 75% and c) 50%, with n = 5 replicates in each case. Removed reads were randomly selected prior to barcode extraction and subsequent population dynamics analysis. Each data point represents one replicate of a single tissue pair, for all pairs within an individual mouse. Values which were negative, 0 or NA were removed prior to plotting. Source data are provided as a Source Data file.

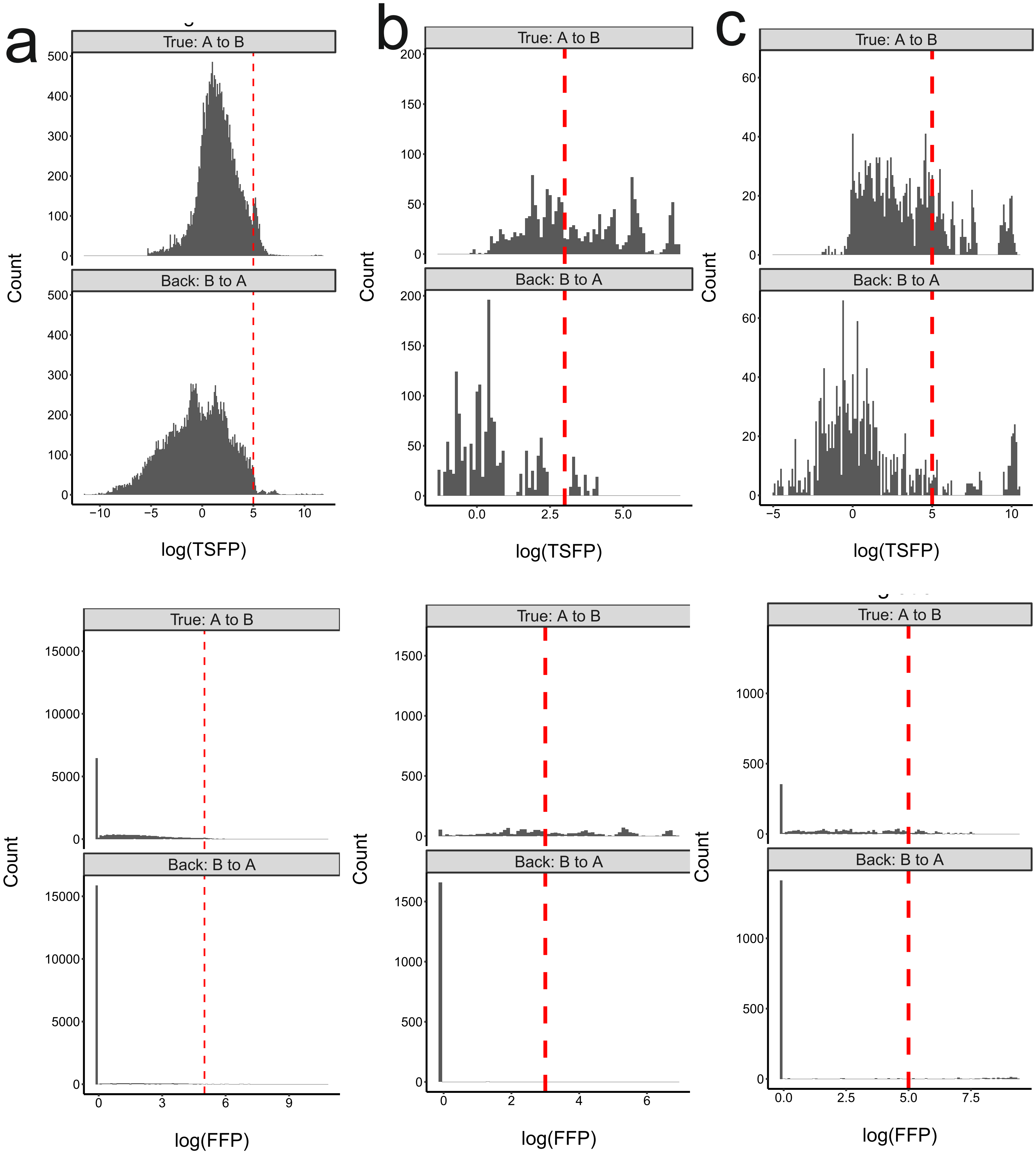


**Supplementary Figure 15: Application of Tissue Sourced Founder Population (TSFP) and Fractional Founder Population (FFP) to three published datasets. a, b, c,** Histograms for TSFP and FFP for true migration (INPUT samples as population A, tissue samples as population B) and back migration (tissue samples as population A, INPUT samples as population B). Data taken from Hotinger et al (a), Lebrun-Corbin et al (b) and Hullahalli and Waldor (c). Red dotted line indicates the threshold applied, based on distribution shifts. All FFP below 1 were changed to 0.9 for visualisation. All TSFP of negative value or NA were changed to 0 for visualisation. Source data are taken from publications as described in Data Availability.

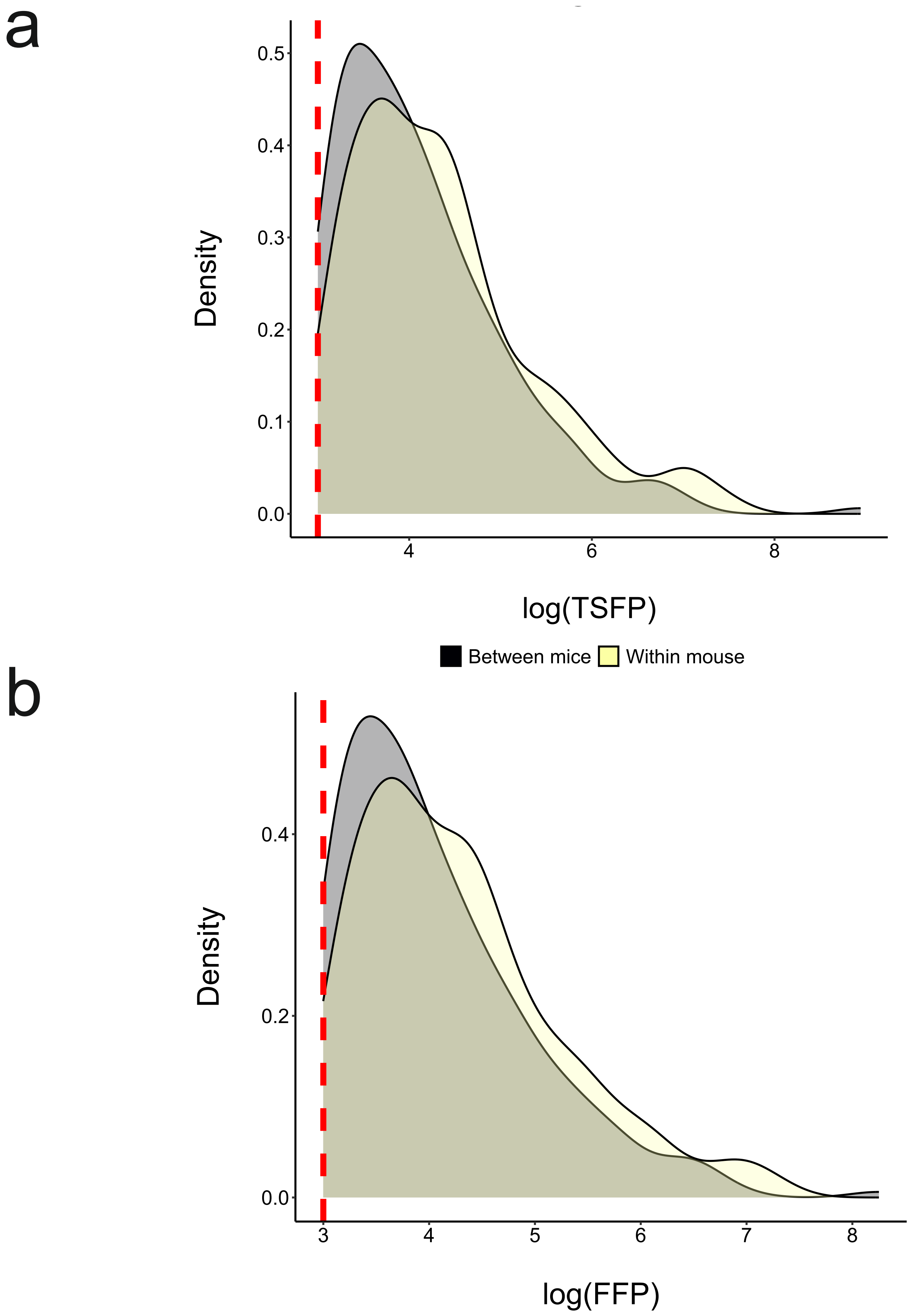


**Supplementary Figure 16: Comparison of within-mouse vs randomised between mouse tissue samples.** a, b, Density plots of Tissue Sourced Founder Population (TSFP) (a) and Fractional Founder Population (FFP) (b) distributions for all within-mouse (yellow) and a randomly selected matched number of between mouse (grey) tissue samples. Values above the cut-off threshold of 20 (log(3); indicated by dotted red line) are shown. Source data are provided as a Source Data file.

## Supplementary Notes

## Contents:

1. A summary of the GLMM model fits tested and the evaluation process for selecting the best model fit for skin biopsy parasite population Shannon Index data.

2. A summary of the GLMM model fits tested and the evaluation process for selecting the best model fit for skin biopsy to visceral tissue genetic distance data.

3. A summary of the GLMM model fits tested and the evaluation process for selecting the best model fit for skin biopsy to visceral tissue TSFP data in single vs re-infected mice.

4. A summary of the GLMM model fits tested and the evaluation process for selecting the best model fit for visceral tissue to skin biopsy TSFP data in single vs re-infected mice.

5. Correlation analysis results for comparing sub-sampled and jack-knifed TSFP calculations with the calculations based on the full set of barcode counts for all samples.

6. Correlation analysis results for comparing TSFP calculation estimates with reduced sequence read depths against calculations using all reads.

### 1. GLMM fits tested in relation to skin Shannon Index:

We fitted a Compound Poisson GLMM for the Shannon Index value for each individual skin biopsy. We compared models as detailed below using the Gini index as our comparison method to select the best model fit for the dataset (see Methods for details).

**Model fits:**

P1

Compound Poisson linear mixed model fit by the Laplace approximation
Formula: Shannon_index ~ group2 + (1 | mouse_ID)
Data: SI_data_sub

| AIC | BIC | logLik | deviance |
|---|---|---|---|
| 164.8 | 176.7 | -78.39 | 156.8 |

Random effects:

| | *Variance* | *Std.Dev.* |
|---|---|---|
| mouse_ID (Intercept) | 0.0017453 | 0.041777 |
| Residual | 0.0637642 | 0.252516 |

Number of obs: 144, groups: mouse_ID, 12

Fixed effects:

| | *Estimate* | *Std. Error* | *t value* |
|---|---|---|---|
| (Intercept) | 0.861263 | 0.031662 | 27.202 |
| group21single_late | 0.009369 | 0.038744 | 0.242 |

Estimated dispersion parameter: 0.0638
Estimated index parameter: 1.01

P2

Compound Poisson linear mixed model fit by the Laplace approximation
Formula: Shannon_index ~ (1 | mouse_ID)
Data: SI_data_sub

| AIC | BIC | logLik | deviance |
|---|---|---|---|
| 162.8 | 171.7 | -78.42 | 156.8 |

Random effects:

| | *Variance* | *Std.Dev.* |
|---|---|---|
| mouse_ID (Intercept) | 0.0017689 | 0.042058 |
| Residual | 0.0637641 | 0.252515 |

Number of obs: 144, groups: mouse_ID, 12

Fixed effects:

| | *Estimate* | *Std. Error* | *t value* |
|---|---|---|---|
| (Intercept) | 0.8675 | 0.0183 | 47.4 |

Estimated dispersion parameter: 0.0638
Estimated index parameter: 1.01

P3

Call:
cpglm(formula = Shannon_index ~ group2, data = SI_data_sub)

Deviance Residuals:

| Min | 1Q | Median | 3Q | Max |
|---|---|---|---|---|
| -1.11582 | -0.12478 | 0.03065 | 0.16439 | 0.58798 |

| | *Estimate* | *Std. Error* | *t value* | *Pr(>\|t\|)* |
|---|---|---|---|---|
| (Intercept) | 0.861869 | 0.024931 | 34.570 | <2e-16 *** |
| group21single_late | 0.009776 | 0.030486 | 0.321 | 0.749 |

---
Signif. codes:  0 ‘***’ 0.001 ‘**’ 0.01 ‘*’ 0.05 ‘.’ 0.1 ‘ ’ 1

Estimated dispersion parameter: 0.074342
Estimated index parameter: 1.01

Residual deviance: 10.636  on 142  degrees of freedom
AIC:  161.73

Number of Fisher Scoring iterations:  4

**Gini comparison results for skin Shannon Index GLMM fits:**

Call:
gini(loss = "Shannon_index", score = paste("P", 1:3, sep = ""),
  data = da)

Gini indices:

| | *P1* | *P2* | *P3* |
|---|---|---|---|
| ***P1*** | 0.000 | 1.125 | -1.996 |
| ***P2*** | -1.040 | 0.000 | -1.957 |
| ***P3*** | 3.492 | 3.469 | 0.000 |

Standard errors:

| | *P1* | *P2* | *P3* |
|---|---|---|---|
| ***P1*** | 0.0000 | 0.8753 | 0.8809 |

| | | | |
|---|---|---|---|
| ***P2*** | 0.9031 | 0.0000 | 0.8657 |
| ***P3*** | 0.8794 | 0.8631 | 0.0000 |

The selected score is P2.

## 2. GLMM fits tested in relation to skin-viscera Genetic Distances:

We fitted a Compound Poisson GLMM for the Genetic Distance value for each individual skin biopsy to visceral tissue relationship. We compared models as detailed below using the Gini index as our comparison method to select the best model fit for the dataset (see Methods for details).

**Model fits:**

P1

Compound Poisson linear mixed model fit by the Laplace approximation
Formula: GD ~ group2 * TISSUE2 + (1 | mouse_ID)
Data: GD_data_sub_skin_to_vis

| AIC | BIC | logLik | deviance |
|---|---|---|---|
| -1534 | -1464 | 781.1 | -1562 |

Random effects:

| | *Variance* | *Std.Dev.* |
|---|---|---|
| mouse_ID (Intercept) | 0.0034386 | 0.058639 |
| Residual | 0.0285451 | 0.168953 |

Number of obs: 1152, groups: mouse_ID, 12

Fixed effects:

| | *Estimate* | *Std. Error* | *t value* |
|---|---|---|---|
| (Intercept) | -0.467752 | 0.036493 | -12.818 |
| group21single_late | -0.109998 | 0.044995 | -2.445 |
| TISSUE2GUT | -0.232130 | 0.040739 | -5.698 |
| TISSUE2LIVER | 0.003861 | 0.037582 | 0.103 |
| TISSUE2LN | -0.247949 | 0.032790 | -7.562 |
| TISSUE2LUNG | -0.073773 | 0.038568 | -1.913 |
| TISSUE2S | -0.089856 | 0.038778 | -2.317 |
| group21single_late:TISSUE2GUT | 0.096089 | 0.050235 | 1.913 |
| group21single_late:TISSUE2LIVER | 0.024956 | 0.046753 | 0.534 |
| group21single_late:TISSUE2LN | 0.107899 | 0.040493 | 2.665 |
| group21single_late:TISSUE2LUNG | -0.067516 | 0.048523 | -1.391 |
| group21single_late:TISSUE2S | 0.159207 | 0.047509 | 3.351 |

Estimated dispersion parameter: 0.0285
Estimated index parameter: 1.0107

P2

Compound Poisson linear mixed model fit by the Laplace approximation
Formula: GD ~ (1 | mouse_ID)
Data: GD_data_sub_skin_to_vis

| **AIC** | **BIC** | **logLik** | **deviance** |
|---|---|---|---|
| -1370 | -1355 | 688 | -1376 |

Random effects:

| | ***Variance*** | ***Std.Dev.*** |
|---|---|---|
| mouse_ID (Intercept) | 0.0041375 | 0.064323 |
| Residual | 0.0316719 | 0.177966 |

Number of obs: 1152, groups: mouse_ID, 12

Fixed effects:

| | ***Estimate*** | ***Std. Error*** | ***t value*** |
|---|---|---|---|
| (Intercept) | -0.61204 | 0.01988 | -30.79 |

Estimated dispersion parameter: 0.0317
Estimated index parameter: 1.0128

P3

Call:
cpglm(formula = GD ~ group2 * TISSUE2, data = GD_data_sub_skin_to_vis)

Deviance Residuals:

| **Min** | **1Q** | **Median** | **3Q** | **Max** |
|---|---|---|---|---|
| -0.75530 | -0.09048 | 0.01684 | 0.11540 | 0.35930 |

| | ***Estimate*** | ***Std. Error*** | ***t value*** | ***Pr(>\|t\|)*** |
|---|---|---|---|---|
| (Intercept) | -0.467069 | 0.021459 | -21.766 | < 2e-16*** |
| group21single_late | -0.108393 | 0.026773 | -4.049 | 5.50e-05*** |
| TISSUE2GUT | -0.232123 | 0.040238 | -5.769 | 1.03e-08*** |
| TISSUE2LIVER | 0.003852 | 0.037120 | 0.104 | 0.917374 |

| | | | | |
|---|---|---|---|---|
| TISSUE2LN | -0.247957 | 0.032387 | -7.656 | 4.07e-14*** |
| TISSUE2LUNG | -0.073737 | 0.038093 | -1.936 | 0.053154 . |
| TISSUE2S | -0.089905 | 0.038302 | -2.347 | 0.019084 * |
| group21single_late:TISSUE2GUT | 0.095992 | 0.049617 | 1.935 | 0.053280 . |
| group21single_late:TISSUE2LIVER | 0.024905 | 0.046178 | 0.539 | 0.589776 |
| group21single_late:TISSUE2LN | 0.107856 | 0.039995 | 2.697 | 0.007105** |
| group21single_late:TISSUE2LUNG | -0.067649 | 0.047926 | -1.412 | 0.158364 |
| group21single_late:TISSUE2S | 0.159230 | 0.046925 | 3.393 | 0.000714*** |

---
Signif. codes:  0 ‘***’ 0.001 ‘**’ 0.01 ‘*’ 0.05 ‘.’ 0.1 ‘ ’ 1

Estimated dispersion parameter: 0.028604
Estimated index parameter: 1.011

Residual deviance: 34.689  on 1140  degrees of freedom
AIC:  -1487.2

Number of Fisher Scoring iterations:  4

P4

Compound Poisson linear mixed model fit by the Laplace approximation
Formula: GD ~ group2 + TISSUE2 + (1 | mouse_ID)
Data: GD_data_sub_skin_to_vis

| **AIC** | **BIC** | **logLik** | **deviance** |
|---|---|---|---|
| -1519 | -1474 | 768.6 | -1537 |

Random effects:

| | ***Variance*** | ***Std.Dev.*** |
|---|---|---|
| mouse_ID (Intercept) | 0.0034391 | 0.058644 |
| Residual | 0.0285638 | 0.169008 |

Number of obs: 1152, groups: mouse_ID, 12

Fixed effects:

| | ***Estimate*** | ***Std. Error*** | ***t value*** |
|---|---|---|---|
| (Intercept) | -0.50112 | 0.03338 | -15.011 |
| group21single_late | -0.05852 | 0.03861 | -1.516 |
| TISSUE2GUT | -0.16936 | 0.02384 | -7.105 |
| TISSUE2LIVER | 0.01996 | 0.02236 | 0.893 |
| TISSUE2LN | -0.17732 | 0.01924 | -9.217 |
| TISSUE2LUNG | -0.11661 | 0.02341 | -4.982 |
| TISSUE2S | 0.01527 | 0.02240 | 0.682 |

Estimated dispersion parameter: 0.0286
Estimated index parameter: 1.0108

P5

Compound Poisson linear mixed model fit by the Laplace approximation
Formula: GD ~ TISSUE2 + (1 | mouse_ID)
Data: GD_data_sub_skin_to_vis

| **AIC** | **BIC** | **logLik** | **deviance** |
|---|---|---|---|
| -1519 | -1479 | 767.5 | -1535 |

Random effects:

| | ***Variance*** | ***Std.Dev.*** |
|---|---|---|
| mouse_ID (Intercept) | 0.0041965 | 0.06478 |
| Residual | 0.0285638 | 0.16901 |

Number of obs: 1152, groups: mouse_ID, 12

Fixed effects:

| | ***Estimate*** | ***Std. Error*** | ***t value*** |
|---|---|---|---|
| (Intercept) | -0.54013 | 0.02278 | -23.715 |
| TISSUE2GUT | -0.16936 | 0.02384 | -7.105 |
| TISSUE2LIVER | 0.01996 | 0.02236 | 0.893 |
| TISSUE2LN | -0.17732 | 0.01924 | -9.217 |
| TISSUE2LUNG | -0.11661 | 0.02341 | -4.982 |
| TISSUE2S | 0.01527 | 0.02240 | 0.682 |

Estimated dispersion parameter: 0.0286
Estimated index parameter: 1.0108

P6

Compound Poisson linear mixed model fit by the Laplace approximation
Formula: GD ~ group2 + (1 | mouse_ID)
Data: GD_data_sub_skin_to_vis

| **AIC** | **BIC** | **logLik** | **deviance** |
|---|---|---|---|
| -1370 | -1350 | 689 | -1378 |

Random effects:

| | *Variance* | *Std.Dev.* |
|---|---|---|
| mouse_ID (Intercept) | 0.0033814 | 0.05815 |
| Residual | 0.0316719 | 0.17797 |

Number of obs: 1152, groups: mouse_ID, 12

Fixed effects:

| | *Estimate* | *Std. Error* | *t value* |
|---|---|---|---|
| (Intercept) | -0.57305 | 0.03147 | -18.207 |
| group21single_late | -0.05848 | 0.03860 | -1.515 |

Estimated dispersion parameter: 0.0317
Estimated index parameter: 1.0128

**Gini comparison results for skin-viscera Genetic Distance GLMM fits:**

Call:
gini(loss = "GD", score = paste("P", 1:6, sep = ""), data = da2)

Gini indices:

| | *P1* | *P2* | *P3* | *P4* | *P5* | *P6* |
|---|---|---|---|---|---|---|
| ***P1*** | 0.00000 | 0.09863 | -0.48629 | -0.11030 | 0.08817 | -0.07302 |
| ***P2*** | 5.05997 | 0.00000 | 4.24413 | 4.47663 | 4.75273 | -0.19458 |
| ***P3*** | 3.56506 | 1.97300 | 0.00000 | 3.13332 | 3.13319 | 1.86715 |
| ***P4*** | 1.98655 | 0.16031 | 0.53377 | 0.00000 | 0.27229 | -0.19119 |
| ***P5*** | 1.80053 | -0.15291 | 0.60288 | -0.09238 | 0.00000 | -0.09410 |
| ***P6*** | 5.22599 | 0.19863 | 4.29882 | 4.78156 | 4.74176 | 0.00000 |

Standard errors:

| | *P1* | *P2* | *P3* | *P4* | *P5* | *P6* |
|---|---|---|---|---|---|---|
| ***P1*** | 0.0000 | 0.3278 | 0.3782 | 0.3815 | 0.3564 | 0.3275 |
| ***P2*** | 0.3332 | 0.0000 | 0.3600 | 0.3126 | 0.3165 | 0.3620 |
| ***P3*** | 0.3851 | 0.3699 | 0.0000 | 0.3691 | 0.3670 | 0.3699 |
| ***P4*** | 0.3827 | 0.3135 | 0.3687 | 0.0000 | 0.3144 | 0.3198 |
| ***P5*** | 0.3586 | 0.3172 | 0.3663 | 0.3142 | 0.0000 | 0.3289 |
| ***P6*** | 0.3331 | 0.3563 | 0.3597 | 0.3187 | 0.3308 | 0.0000 |

The selected score is P1.

## 3. GLMM fits tested in relation to skin to visceral TSFP in reinfection:

We fitted a Compound Poisson GLMM for the TSFP value for each individual skin biopsy to each visceral tissue. We compared models as detailed below using the Gini index as our comparison method to select the best model fit for the dataset (see Methods for details).

**Model fits:**

P1

Compound Poisson linear mixed model fit by the Laplace approximation
Formula: TSFP ~ group2 * OUTPUT3 + (1 | mouse_ID)
Data: reinf3_df_sub_skin_in

| AIC | BIC | logLik | deviance |
|---|---|---|---|
| 4436 | 4505 | -2204 | 4408 |

Random effects:

| | *Variance* | *Std.Dev.* |
|---|---|---|
| mouse_ID (Intercept) | 0.37412 | 0.61166 |
| Residual | 5.00371 | 2.23690 |

Number of obs: 1001, groups: mouse_ID, 12

Fixed effects:

| | *Estimate* | *Std. Error* | *t value* |
|---|---|---|---|
| (Intercept) | 1.53980 | 0.34236 | 4.498 |
| group21single_late | -0.48714 | 0.42467 | -1.147 |
| OUTPUT3GUT | -0.04698 | 0.43414 | -0.108 |
| OUTPUT3LIVER | 0.26115 | 0.25417 | 1.027 |
| OUTPUT3LN | -0.50903 | 0.23204 | -2.194 |
| OUTPUT3LUNG | 0.67794 | 0.23829 | 2.845 |
| OUTPUT3S | 0.19828 | 0.27921 | 0.710 |
| group21single_late:OUTPUT3GUT | 0.50007 | 0.48842 | 1.024 |
| group21single_late:OUTPUT3LIVER | -0.05380 | 0.33024 | -0.163 |
| group21single_late:OUTPUT3LN | -0.38651 | 0.30595 | -1.263 |
| group21single_late:OUTPUT3LUNG | -1.70461 | 0.34875 | -4.888 |
| group21single_late:OUTPUT3S | -0.42328 | 0.37584 | -1.126 |

Estimated dispersion parameter: 5.0037
Estimated index parameter: 1.4939

P2

Compound Poisson linear mixed model fit by the Laplace approximation
Formula: TSFP ~ OUTPUT3 + (1 | mouse_ID)

Data: reinf3_df_sub_skin_in

| AIC | BIC | logLik | deviance |
|---|---|---|---|
| 4458 | 4498 | -2221 | 4442 |

Random effects:

| | *Variance* | *Std.Dev.* |
|---|---|---|
| mouse_ID (Intercept) | 0.52037 | 0.72136 |
| Residual | 5.11540 | 2.26172 |

Number of obs: 1001, groups: mouse_ID, 12

Fixed effects:

| | *Estimate* | *Std. Error* | *t value* |
|---|---|---|---|
| (Intercept) | 1.18697 | 0.23129 | 5.132 |
| OUTPUT3GUT | 0.43122 | 0.19882 | 2.169 |
| OUTPUT3LIVER | 0.23425 | 0.16471 | 1.422 |
| OUTPUT3LN | -0.72571 | 0.15312 | -4.739 |
| OUTPUT3LUNG | -0.07961 | 0.17068 | -0.466 |
| OUTPUT3S | -0.02579 | 0.18953 | -0.136 |

Estimated dispersion parameter: 5.1154
Estimated index parameter: 1.5007

P3

Compound Poisson linear mixed model fit by the Laplace approximation
Formula: TSFP ~ group2 + (1 | mouse_ID)
Data: reinf3_df_sub_skin_in

| AIC | BIC | logLik | deviance |
|---|---|---|---|
| 4491 | 4511 | -2242 | 4483 |

Random effects:

| | *Variance* | *Std.Dev.* |
|---|---|---|
| mouse_ID (Intercept) | 0.34226 | 0.58503 |
| Residual | 5.28004 | 2.29783 |

Number of obs: 1001, groups: mouse_ID, 12

Fixed effects:

| | *Estimate* | *Std. Error* | *t value* |
|---|---|---|---|
| (Intercept) | 1.6297 | 0.3047 | 5.349 |
| group21single_late | -0.7735 | 0.3757 | -2.059 |

Estimated dispersion parameter: 5.28
Estimated index parameter: 1.5076

P4

Call:
cpglm(formula = TSFP ~ group2 * OUTPUT3, data = reinf3_df_sub_skin_in)
Deviance Residuals:

| Min | 1Q | Median | 3Q | Max |
|---|---|---|---|---|
| -3.5647 | -2.4171 | -1.4072 | -0.2097 | 23.0351 |

| | *Estimate* | *Std. Error* | *t value* | *Pr(>\|t\|)* |
|---|---|---|---|---|
| (Intercept) | 1.5767 | 0.3678 | 4.287 | 1.99e-05*** |
| group21single_late | -0.2573 | 0.4669 | -0.551 | 0.5817 |
| OUTPUT3GUT | 0.4237 | 1.0070 | 0.421 | 0.6740 |
| OUTPUT3LIVER | 0.2602 | 0.6110 | 0.426 | 0.6703 |
| OUTPUT3LN | -0.4200 | 0.5491 | -0.765 | 0.4445 |
| OUTPUT3LUNG | 0.7404 | 0.5686 | 1.302 | 0.1932 |
| OUTPUT3S | 0.3344 | 0.6643 | 0.503 | 0.6148 |
| group21single_late:OUTPUT3GUT | 0.1783 | 1.1311 | 0.158 | 0.8748 |
| group21single_late:OUTPUT3LIVER | -0.1637 | 0.7908 | -0.207 | 0.8361 |
| group21single_late:OUTPUT3LN | -0.4038 | 0.7129 | -0.566 | 0.5713 |
| group21single_late:OUTPUT3LUNG | -1.6832 | 0.8077 | -2.084 | 0.0374 * |
| group21single_late:OUTPUT3S | -0.7420 | 0.8818 | -0.841 | 0.4003 |

---
Signif. codes:  0 ‘***’ 0.001 ‘**’ 0.01 ‘*’ 0.05 ‘.’ 0.1 ‘ ’ 1
Estimated dispersion parameter: 5.3427
Estimated index parameter: 1.5089
Residual deviance: 5739.8  on 989  degrees of freedom
AIC:  4496.6
Number of Fisher Scoring iterations:  5

P5

Call:
cpglm(formula = TSFP ~ group2, data = reinf3_df_sub_skin_in)

Deviance Residuals:

| Min | 1Q | Median | 3Q | Max |
|---|---|---|---|---|
| -3.0943 | -2.6769 | -1.4734 | -0.1859 | 23.9987 |

| | *Estimate* | *Std. Error* | *t value* | *Pr(>\|t\|)* |
|---|---|---|---|---|
| (Intercept) | 1.7273 | 0.2094 | 8.249 | 5.02e-16 *** |
| group21single_late | -0.6137 | 0.2710 | -2.264 | 0.0238 * |

---
Signif. codes:  0 ‘***’ 0.001 ‘**’ 0.01 ‘*’ 0.05 ‘.’ 0.1 ‘ ’ 1

Estimated dispersion parameter: 5.645
Estimated index parameter: 1.5278
Residual deviance: 6101 on 999 degrees of freedom
AIC: 4549.6
Number of Fisher Scoring iterations: 5

P6

Compound Poisson linear mixed model fit by the Laplace approximation
Formula: TSFP ~ (1 | mouse_ID)
Data: reinf3_df_sub_skin_in

| AIC | BIC | logLik | deviance |
|---|---|---|---|
| 4493 | 4507 | -2243 | 4487 |

Random effects:

| | *Variance* | *Std.Dev.* |
|---|---|---|
| mouse_ID (Intercept) | 0.4787 | 0.69188 |
| Residual | 5.2800 | 2.29783 |

Number of obs: 1001, groups: mouse_ID, 12

Fixed effects:

| | *Estimate* | *Std. Error* | *t value* |
|---|---|---|---|
| (Intercept) | 1.1122 | 0.2078 | 5.353 |

Estimated dispersion parameter: 5.28
Estimated index parameter: 1.5076

P7

Call:
cpglm(formula = TSFP ~ OUTPUT3, data = reinf3_df_sub_skin_in)
Deviance Residuals:

| Min | 1Q | Median | 3Q | Max |
|---|---|---|---|---|
| -3.2494 | -2.8744 | -1.3579 | -0.1165 | 21.8259 |

| | *Estimate* | *Std. Error* | *t value* | *Pr(>\|t\|)* |
|---|---|---|---|---|
| (Intercept) | 1.41729 | 0.23374 | 6.064 | 1.89e-09 *** |
| OUTPUT3GUT | 0.51775 | 0.46618 | 1.111 | 0.2670 |
| OUTPUT3LIVER | 0.17412 | 0.39983 | 0.435 | 0.6633 |
| OUTPUT3LN | -0.63777 | 0.35852 | -1.779 | 0.0756 . |
| OUTPUT3LUNG | 0.05837 | 0.39555 | 0.148 | 0.8827 |
| OUTPUT3S | -0.04966 | 0.44482 | -0.112 | 0.9111 |

---
Signif. codes:  0 ‘***’ 0.001 ‘**’ 0.01 ‘*’ 0.05 ‘.’ 0.1 ‘ ’ 1
Estimated dispersion parameter: 5.6138
Estimated index parameter: 1.5263
Residual deviance: 6061.8  on 995  degrees of freedom
AIC:  4550.1
Number of Fisher Scoring iterations:  5

P8

Compound Poisson linear mixed model fit by the Laplace approximation
Formula: TSFP ~ group2 + OUTPUT3 + (1 | mouse_ID)
Data: reinf3_df_sub_skin_in

| AIC | BIC | logLik | deviance |
|---|---|---|---|
| 4456 | 4501 | -2219 | 4438 |

Random effects:

| | *Variance* | *Std.Dev.* |
|---|---|---|
| mouse_ID (Intercept) | 0.35646 | 0.59704 |
| Residual | 5.11559 | 2.26177 |

Number of obs: 1001, groups: mouse_ID, 12

Fixed effects:

| | *Estimate* | *Std. Error* | *t value* |
|---|---|---|---|
| (Intercept) | 1.75174 | 0.32063 | 5.463 |
| group21single_late | -0.84354 | 0.38269 | -2.204 |
| OUTPUT3GUT | 0.44847 | 0.19874 | 2.256 |
| OUTPUT3LIVER | 0.23298 | 0.16468 | 1.415 |
| OUTPUT3LN | -0.72431 | 0.15302 | -4.733 |
| OUTPUT3LUNG | -0.08717 | 0.17081 | -0.510 |
| OUTPUT3S | -0.02957 | 0.18949 | -0.156 |

Estimated dispersion parameter: 5.1156
Estimated index parameter: 1.5007

**Gini comparison results for skin to viscera TSFP GLMM fits:**

Call:
gini(loss = "TSFP", score = paste("P", 1:8, sep = ""), data = da)

Gini indices:

| | *P1* | *P2* | *P3* | *P4* | *P5* | *P6* | *P7* | *P8* |
|---|---|---|---|---|---|---|---|---|
| ***P1*** | 0.00000 | 2.65081 | 4.05855 | -0.04747 | 2.06662 | 4.13542 | 2.85748 | 1.38388 |
| ***P2*** | 13.66257 | 0.00000 | 4.54839 | 10.33106 | 3.49489 | 6.04794 | 1.52389 | 0.18518 |
| ***P3*** | 24.61856 | 17.15413 | 0.00000 | 16.05875 | -0.74920 | 3.28107 | 9.19352 | 15.67746 |
| ***P4*** | 25.92865 | 22.45120 | 20.12714 | 0.00000 | -0.48279 | 20.97886 | -0.48279 | 22.57387 |

| | | | | | | | | |
|---|---|---|---|---|---|---|---|---|
| ***P5*** | 35.98982 | 32.59930 | 29.19456 | 24.60836 | 0.00000 | 29.19456 | 13.68647 | 32.82747 |
| ***P6*** | 24.12818 | 15.67643 | 1.51092 | 15.96165 | 0.15179 | 0.00000 | 9.20453 | 15.33018 |
| ***P7*** | 31.90282 | 30.51184 | 28.13307 | 22.66720 | 11.16071 | 27.84670 | 0.00000 | 30.83910 |
| ***P8*** | 15.26661 | 1.88281 | 6.14538 | 9.49649 | 2.29875 | 6.49351 | 1.47162 | 0.00000 |

Standard errors:

| | ***P1*** | ***P2*** | ***P3*** | ***P4*** | ***P5*** | ***P6*** | ***P7*** | ***P8*** |
|---|---|---|---|---|---|---|---|---|
| ***P1*** | 0.000 | 6.950 | 6.083 | 8.315 | 8.284 | 6.430 | 8.205 | 7.105 |
| ***P2*** | 6.626 | 0.000 | 4.889 | 6.231 | 6.304 | 5.382 | 5.947 | 6.668 |
| ***P3*** | 5.652 | 4.342 | 0.000 | 5.939 | 8.112 | 7.568 | 5.971 | 4.850 |
| ***P4*** | 6.740 | 5.353 | 5.496 | 0.000 | 6.952 | 5.450 | 7.851 | 5.338 |
| ***P5*** | 6.409 | 5.307 | 6.608 | 6.268 | 0.000 | 6.608 | 5.531 | 5.439 |
| ***P6*** | 5.849 | 4.796 | 8.675 | 5.957 | 8.152 | 0.000 | 6.100 | 5.120 |
| ***P7*** | 6.467 | 5.371 | 5.696 | 6.517 | 5.507 | 5.795 | 0.000 | 5.259 |
| ***P8*** | 6.750 | 6.504 | 5.450 | 6.208 | 6.405 | 5.674 | 5.772 | 0.000 |

The selected score is P1.

## 4. GLMM fits tested in relation to visceral to skin TSFP in reinfection:

We fitted a Compound Poisson GLMM for the TSFP value for each visceral tissue to each individual skin biopsy. We compared models as detailed below using the Gini index as our comparison method to select the best model fit for the dataset (see Methods for details).

**Model fits:**

P1

Compound Poisson linear mixed model fit by the Laplace approximation
Formula: TSFP ~ group2 * INPUT3 + (1 | mouse_ID)
Data: reinf3_df_sub_skin_out

| **AIC** | **BIC** | **logLik** | **deviance** |
|---|---|---|---|
| 1521 | 1589 | -746.3 | 1493 |

Random effects:

| | ***Variance*** | ***Std.Dev.*** |
|---|---|---|
| mouse_ID (Intercept) | 2.7981 | 1.6727 |
| Residual | 22.2385 | 4.7158 |

Number of obs: 1001, groups: mouse_ID, 12

Fixed effects:

| | ***Estimate*** | ***Std. Error*** | ***t value*** |
|---|---|---|---|
| (Intercept) | -4.994 | 1.475 | -3.386 |

| | | | |
|---|---|---|---|
| group21single_late | 5.285 | 1.621 | 3.259 |
| INPUT3GUT | -21.096 | 185.399 | -0.114 |
| INPUT3LIVER | 2.401 | 1.618 | 1.484 |
| INPUT3LN | 6.105 | 1.265 | 4.825 |
| INPUT3LUNG | 6.179 | 1.315 | 4.698 |
| INPUT3S | 3.310 | 1.544 | 2.143 |
| group21single_late:INPUT3GUT | 21.192 | 185.400 | 0.114 |
| group21single_late:INPUT3LIVER | -5.334 | 1.801 | -2.961 |
| group21single_late:INPUT3LN | -7.585 | 1.357 | -5.591 |
| group21single_late:INPUT3LUNG | -5.507 | 1.396 | -3.946 |
| group21single_late:INPUT3S | -5.724 | 1.818 | -3.149 |

Estimated dispersion parameter: 22.2385
Estimated index parameter: 1.6118

P2

Compound Poisson linear mixed model fit by the Laplace approximation
Formula: TSFP ~ INPUT3 + (1 | mouse_ID)
Data: reinf3_df_sub_skin_out

| AIC | BIC | logLik | deviance |
|---|---|---|---|
| 1544 | 1583 | -764 | 1528 |

Random effects:

| | *Variance* | *Std.Dev.* |
|---|---|---|
| mouse_ID (Intercept) | 3.112 | 1.7641 |
| Residual | 25.606 | 5.0602 |

Number of obs: 1001, groups: mouse_ID, 12
Fixed effects:

| | *Estimate* | *Std. Error* | *t value* |
|---|---|---|---|
| (Intercept) | -0.4094 | 0.5982 | -0.684 |
| INPUT3GUT | 0.5863 | 0.6108 | 0.960 |
| INPUT3LIVER | -2.1669 | 0.6920 | -3.131 |
| INPUT3LN | 0.3897 | 0.4067 | 0.958 |
| INPUT3LUNG | 1.5242 | 0.4338 | 3.514 |
| INPUT3S | -1.4638 | 0.7555 | -1.937 |

Estimated dispersion parameter: 25.6057
Estimated index parameter: 1.634

P3

Compound Poisson linear mixed model fit by the Laplace approximation
Formula: TSFP ~ group2 + (1 | mouse_ID)
Data: reinf3_df_sub_skin_out

| AIC | BIC | logLik | deviance |
|---|---|---|---|

| | | | |
|---|---|---|---|
| 1568 | 1587 | -779.9 | 1560 |

Random effects:

| | *Variance* | *Std.Dev.* |
|---|---|---|
| *mouse_ID (Intercept)* | 2.7787 | 1.6669 |
| *Residual* | 28.9063 | 5.3765 |

Number of obs: 1001, groups: mouse_ID, 12

Fixed effects:

| | ***Estimate*** | ***Std. Error*** | ***t value*** |
|---|---|---|---|
| (Intercept) | 0.3072 | 0.8818 | 0.348 |
| group21single_late | -0.4243 | 1.0845 | -0.391 |

Estimated dispersion parameter: 28.9063
Estimated index parameter: 1.6465

P4

Call:
cpglm(formula = TSFP ~ group2 * INPUT3, data = reinf3_df_sub_skin_out)
Deviance Residuals:

| **Min** | **1Q** | **Median** | **3Q** | **Max** |
|---|---|---|---|---|
| -3.547 | -3.259 | -2.174 | -1.669 | 15.706 |

| | ***Estimate*** | ***Std. Error*** | ***t value*** | ***Pr(>\|t\|)*** |
|---|---|---|---|---|
| (Intercept) | -4.492 | 1.217 | -3.691 | 0.000236*** |
| group21single_late | 6.740 | 1.249 | 5.398 | 8.43e-08 *** |
| INPUT3GUT | -22.810 | 150.460 | -0.152 | 0.879531 |
| INPUT3LIVER | 2.401 | 1.665 | 1.442 | 0.149606 |
| INPUT3LN | 5.997 | 1.291 | 4.645 | 3.86e-06 *** |
| INPUT3LUNG | 6.252 | 1.350 | 4.633 | 4.09e-06*** |
| INPUT3S | 3.513 | 1.628 | 2.158 | 0.031205* |
| group21single_late:INPUT3GUT | 21.477 | 150.462 | 0.143 | 0.886527 |
| group21single_late:INPUT3LIVER | -6.259 | 1.866 | -3.355 | 0.000824*** |
| group21single_late:INPUT3LN | -8.823 | 1.397 | -6.317 | 4.01e-10*** |
| group21single_late:INPUT3LUNG | -6.736 | 1.439 | -4.682 | 3.24e-06*** |
| group21single_late:INPUT3S | -7.868 | 1.899 | -4.143 | 3.72e-05*** |

---
Signif. codes: 0 '***' 0.001 '**' 0.01 '*' 0.05 '.' 0.1 ' ' 1
Estimated dispersion parameter: 28.565
Estimated index parameter: 1.6536
Residual deviance: 8253.9 on 989 degrees of freedom
AIC: 1548.5
Number of Fisher Scoring iterations: 25

P5

Call:
cpglm(formula = TSFP ~ group2, data = reinf3_df_sub_skin_out)

Deviance Residuals:

| Min | 1Q | Median | 3Q | Max |
|---|---|---|---|---|
| -3.153 | -3.153 | -3.153 | -2.903 | 21.003 |

| | *Estimate* | *Std. Error* | *t value* | *Pr(>\|t\|)* |
|---|---|---|---|---|
| (Intercept) | 0.7788 | 0.3731 | 2.087 | 0.0371 * |
| group21single_late | 0.5515 | 0.4459 | 1.237 | 0.2164 |

---
Signif. codes: 0 '***' 0.001 '**' 0.01 '*' 0.05 '.' 0.1 ' ' 1

Estimated dispersion parameter: 39.953
Estimated index parameter: 1.7002

Residual deviance: 11339 on 999 degrees of freedom
AIC: 1607

Number of Fisher Scoring iterations: 16

P6

Compound Poisson linear mixed model fit by the Laplace approximation
Formula: TSFP ~ (1 | mouse_ID)
Data: reinf3_df_sub_skin_out

| AIC | BIC | logLik | deviance |
|---|---|---|---|
| 1566 | 1581 | -779.9 | 1560 |

Random effects:

| | *Variance* | *Std.Dev.* |
|---|---|---|
| mouse_ID (Intercept) | 2.7739 | 1.6655 |
| Residual | 28.9174 | 5.3775 |

Number of obs: 1001, groups: mouse_ID, 12

Fixed effects:

| | *Estimate* | *Std. Error* | *t value* |
|---|---|---|---|
| (Intercept) | 0.03045 | 0.51279 | 0.059 |

Estimated dispersion parameter: 28.9174
Estimated index parameter: 1.6464

P7

Call:

cpglm(formula = TSFP ~ INPUT3, data = reinf3_df_sub_skin_out)

Deviance Residuals:

| Min | 1Q | Median | 3Q | Max |
|---|---|---|---|---|
| -3.336 | -3.336 | -2.768 | -1.927 | 17.959 |

| | *Estimate* | *Std. Error* | *t value* | *Pr(>\|t\|)* |
|---|---|---|---|---|
| (Intercept) | 1.81736 | 0.26899 | 6.756 | 2.41e-11 *** |
| INPUT3GUT | -1.08537 | 0.68086 | -1.594 | 0.1112 |
| INPUT3LIVER | -3.57850 | 0.74445 | -4.807 | 1.77e-06 *** |
| INPUT3LN | -1.15310 | 0.42124 | -2.737 | 0.0063 ** |
| INPUT3LUNG | -0.05429 | 0.46202 | -0.118 | 0.9065 |
| INPUT3S | -3.39162 | 0.79226 | -4.281 | 2.04e-05 *** |

---
Signif. codes:  0 '***' 0.001 '**' 0.01 '*' 0.05 '.' 0.1 ' ' 1

Estimated dispersion parameter: 34.166
Estimated index parameter: 1.6764

Residual deviance: 9820.7  on 995  degrees of freedom
AIC:  1580.8

Number of Fisher Scoring iterations:  19

P8

Compound Poisson linear mixed model fit by the Laplace approximation
Formula: TSFP ~ group2 + INPUT3 + (1 | mouse_ID)
Data: reinf3_df_sub_skin_out

| AIC | BIC | logLik | deviance |
|---|---|---|---|
| 1546 | 1590 | -763.9 | 1528 |

Random effects:

| | *Variance* | *Std.Dev.* |
|---|---|---|
| mouse_ID (Intercept) | 3.1086 | 1.7631 |
| Residual | 25.5921 | 5.0589 |

Number of obs: 1001, groups: mouse_ID, 12

Fixed effects:

| | *Estimate* | *Std. Error* | *t value* |
|---|---|---|---|
| (Intercept) | -0.07126 | 0.96014 | -0.074 |
| group21single_late | -0.50772 | 1.14028 | -0.445 |
| INPUT3GUT | 0.60145 | 0.61019 | 0.986 |
| INPUT3LIVER | -2.17248 | 0.69151 | -3.142 |
| INPUT3LN | 0.37101 | 0.40671 | 0.912 |

| | | | |
|---|---|---|---|
| INPUT3LUNG | 1.52061 | 0.43337 | 3.509 |
| INPUT3S | -1.47131 | 0.75548 | -1.948 |

Estimated dispersion parameter: 25.5921
Estimated index parameter: 1.634

**Gini comparison results for viscera to skin TSFP GLMM fits:**

Call:
gini(loss = "TSFP", score = paste("P", 1:8, sep = ""), data = da)

Gini indices:

| | ***P1*** | ***P2*** | ***P3*** | ***P4*** | ***P5*** | ***P6*** | ***P7*** | ***P8*** |
|---|---|---|---|---|---|---|---|---|
| ***P1*** | 0.000 | -14.245 | 30.761 | 13.151 | 9.080 | 27.881 | 10.138 | -14.140 |
| ***P2*** | 65.223 | 0.000 | 39.362 | 56.282 | 40.384 | 39.421 | 36.891 | 25.085 |
| ***P3*** | 43.740 | 24.716 | 0.000 | 54.959 | -15.536 | -12.732 | 34.469 | 24.389 |
| ***P4*** | 71.737 | 68.196 | 69.069 | 0.000 | -5.359 | 69.000 | -5.359 | 68.228 |
| ***P5*** | 79.688 | 73.738 | 65.415 | 53.607 | 0.000 | 65.415 | 39.626 | 73.700 |
| ***P6*** | 45.003 | 24.513 | -12.532 | 55.024 | -15.388 | 0.000 | 34.479 | 24.352 |
| ***P7*** | 77.502 | 73.580 | 70.046 | 32.237 | 6.471 | 70.305 | 0.000 | 73.257 |
| ***P8*** | 65.046 | -18.648 | 39.076 | 56.062 | 40.199 | 39.052 | 36.589 | 0.000 |

Standard errors:

| | ***P1*** | ***P2*** | ***P3*** | ***P4*** | ***P5*** | ***P6*** | ***P7*** | ***P8*** |
|---|---|---|---|---|---|---|---|---|
| ***P1*** | 0.000 | 6.207 | 7.571 | 7.901 | 8.689 | 8.427 | 8.494 | 6.204 |
| ***P2*** | 3.746 | 0.000 | 8.419 | 5.020 | 6.570 | 8.445 | 7.070 | 7.360 |
| ***P3*** | 5.405 | 7.227 | 0.000 | 4.011 | 13.833 | 8.899 | 5.884 | 7.534 |
| ***P4*** | 4.158 | 3.357 | 3.916 | 0.000 | 9.949 | 3.924 | 7.991 | 3.353 |
| ***P5*** | 3.327 | 3.464 | 5.093 | 5.253 | 0.000 | 5.093 | 5.271 | 3.469 |
| ***P6*** | 5.598 | 7.279 | 7.859 | 4.015 | 13.838 | 0.000 | 5.852 | 7.542 |
| ***P7*** | 3.405 | 3.782 | 4.317 | 6.708 | 7.267 | 4.276 | 0.000 | 3.887 |
| ***P8*** | 3.750 | 7.242 | 8.723 | 5.028 | 6.584 | 8.721 | 7.110 | 0.000 |

The selected score is P1.

## 5. Correlation analysis for jack-knifed and sub-sampled TSFP/FFP estimation against TSFP calculations using all barcode counts:

Two-sided Kendall correlation tests of all data points, comparing 100% barcodes used to sub-sampled versions and jack-knifed versions of the data, with percentage included as shown (removal of barcodes at random, with 20 repeats at each percentage for sub-sampling approach).

**TSFP:**

Kendall's rank correlation tau for 95% barcodes vs 100% barcodes:

data: FP_df$FP and FP_df$FP_all

z = 443.46, p-value < 2.2e-16

alternative hypothesis: true tau is not equal to 0

sample estimates:

tau

0.9128141

Kendall's rank correlation tau for 90% barcodes vs 100% barcodes:

data: FP_df2$FP and FP_df2$FP_all

z = 415.78, p-value < 2.2e-16

alternative hypothesis: true tau is not equal to 0

sample estimates:

tau

0.855836

Kendall's rank correlation tau for 75% barcodes vs 100% barcodes:

data: FP_df3$FP and FP_df3$FP_all

z = 364.36, p-value < 2.2e-16

alternative hypothesis: true tau is not equal to 0

sample estimates:

tau

0.7499841

Kendall's rank correlation tau for 50% barcodes vs 100% barcodes:

data: FP_df4$FP and FP_df4$FP_all

z = 264.56, p-value < 2.2e-16

alternative hypothesis: true tau is not equal to 0

sample estimates:

tau

0.5445575

**FFP:**

Kendall's rank correlation tau for 95% barcodes vs 100% barcodes:

data: FP_df$FFP and FP_df$FFP_all

z = 206.52, p-value < 2.2e-16

alternative hypothesis: true tau is not equal to 0

sample estimates:

tau

0.8685851

Kendall's rank correlation tau for 90% of barcodes vs 100% of barcodes:

data: FP_df2$FFP and FP_df2$FFP_all

z = 350.23, p-value < 2.2e-16

alternative hypothesis: true tau is not equal to 0

sample estimates:

tau

0.8885816

Kendall's rank correlation tau 75% of barcodes vs 100% of barcodes:

data: FP_df3$FFP and FP_df3$FFP_all

z = 140.72, p-value < 2.2e-16

alternative hypothesis: true tau is not equal to 0

sample estimates:

tau

0.5918341

Kendall's rank correlation tau 50% of barcodes vs 100% of barcodes:

data:  FP_df4$FFP and FP_df4$FFP_all

z = 74.243, p-value < 2.2e-16

alternative hypothesis: true tau is not equal to 0

sample estimates:

tau

0.3141112

**Jacknife correlation (1 barcode removed, iteratively across all barcodes):**

data:  new_FFP_full1$FFP and new_FFP_full1$all_FFP

z = 532.04, p-value < 2.2e-16

alternative hypothesis: true tau is not equal to 0

sample estimates:

tau

0.9791117

6. **Correlation analysis for TSFP estimates calculated using reduced sequence reads vs calculations using all sequence reads:**

Two-sided Kendall correlation tests of all data points, comparing TSFP calculation using 100% of sequence reads to randomly selected subsets of 90%, 75% and 50% of total reads (n = 5 replicates in each case).

**90% read depth:**

Kendall's rank correlation tau

data:  long_df$FP and long_df$FP_est

z = 233.74, p-value < 2.2e-16

alternative hypothesis: true tau is not equal to 0

sample estimates:

tau

0.962295

**50% read depth:**

Kendall's rank correlation tau

data: long_df$FP_100_percent and long_df$FP_est

z = 210.73, p-value < 2.2e-16

alternative hypothesis: true tau is not equal to 0

sample estimates:

tau

0.8715691

**75% read depth:**

Kendall's rank correlation tau

data: long_df$FP_100_percent and long_df$FP_est

z = 225.27, p-value < 2.2e-16

alternative hypothesis: true tau is not equal to 0

sample estimates:

tau

0.9274098
