# Supplementary material for: Bi-directional highways and super-seeder tissues underpin parasite dissemination in experimental Leishmania donovani infection
Source: Nat Commun. 2026 Aug 26;17:10194. doi: 10.1038/s41467-026-77072-4 (PMC13612394; doi:10.1038/s41467-026-77072-4)
Supplement: Supplementary file 2 [file 41467_2026_77072_MOESM4_ESM.pdf]

nature portfolio

Corresponding author(s): Paul Kaye; Jeremy Mottram

Last updated by author(s): Jun 25, 2026

Reporting Summary

Nature Portfolio wishes to improve the reproducibility of the work that we publish. This form provides structure for consistency and transparency in reporting. For further information on Nature Portfolio policies, see our Editorial Policies and the Editorial Policy Checklist.

Statistics

For all statistical analyses, confirm that the following items are present in the figure legend, table legend, main text, or Methods section.

n/a	Confirmed	
☐	☒	The exact sample size (*n*) for each experimental group/condition, given as a discrete number and unit of measurement
☐	☒	A statement on whether measurements were taken from distinct samples or whether the same sample was measured repeatedly
☐	☒	The statistical test(s) used AND whether they are one- or two-sided<br>*Only common tests should be described solely by name; describe more complex techniques in the Methods section.*
☐	☒	A description of all covariates tested
☐	☒	A description of any assumptions or corrections, such as tests of normality and adjustment for multiple comparisons
☐	☒	A full description of the statistical parameters including central tendency (e.g. means) or other basic estimates (e.g. regression coefficient) AND variation (e.g. standard deviation) or associated estimates of uncertainty (e.g. confidence intervals)
☐	☒	For null hypothesis testing, the test statistic (e.g. *F*, *t*, *r*) with confidence intervals, effect sizes, degrees of freedom and *P* value noted<br>*Give P values as exact values whenever suitable.*
☒	☐	For Bayesian analysis, information on the choice of priors and Markov chain Monte Carlo settings
☒	☐	For hierarchical and complex designs, identification of the appropriate level for tests and full reporting of outcomes
☒	☐	Estimates of effect sizes (e.g. Cohen's *d*, Pearson's *r*), indicating how they were calculated

Our web collection on statistics for biologists contains articles on many of the points above.

Software and code

Policy information about availability of computer code

Data collection	For IVIS data collection (luciferase expression to quantify parasite burdens), we used Living Image version 4.7.4.21053.<br>For confocal skin imaging of tdTomato expression, we used Zen Version 3.9.
Data analysis	Data analysis for for IVIS data was performed using Living Image version 4.7.4.21053. Data analysis for confocal skin imaging was performed using Zen Blue Version 3.9.101.02000.<br>MATLAB simulations were performed using MATLAB/2024a.<br>Analysis of raw sequence reads to quantify barcode abundances was performed using Rust version 0.2.1, as detailed on Github: https://github.com/uoy-research/fqbarcode (10.5281/zenodo.20843221)<br>When down-sampling the sequencing depth prior to barcode extraction, we used SeqKit version 2.3.1 before applying the fqbarcode pipeline detailed above.<br>Analysis of barcode count datasets was performed using R version 4.4.3, as detailed on Github: https://github.com/CiaraLoughrey-scientist/barseq-Leishmania-2025 (10.5281/zenodo.20843226)

For manuscripts utilizing custom algorithms or software that are central to the research but not yet described in published literature, software must be made available to editors and reviewers. We strongly encourage code deposition in a community repository (e.g. GitHub). See the Nature Portfolio guidelines for submitting code & software for further information.

Data

Policy information about availability of data

All manuscripts must include a data availability statement. This statement should provide the following information, where applicable:

- Accession codes, unique identifiers, or web links for publicly available datasets
- A description of any restrictions on data availability
- For clinical datasets or third party data, please ensure that the statement adheres to our policy

Summary statistical data and raw read counts for barcode abundances are available in Supplementary or Source Data files included with the manuscript and/or at the Github account: https://github.com/CiaraLoughrey-scientist/barseq-Leishmania-2025. All details of our analysis, data used and the code are included in this Github, except the three datasets relating to our "Validation" via the re-analysis of published datasets. Two of these are publicly available with the papers referred to in Methods, whilst one (for the paper Hotinger, J. A., Campbell, I. W., Hullahalli, K., Osaki, A. & Waldor, M. K. Quantification of Salmonella enterica serovar Typhimurium population dynamics in murine infection using a highly diverse barcoded library. eLife 13, RP101388 (2025)) was obtained directly from the study authors via email correspondence. We have not included these datasets in our Github as they are not publicly available, but requests can be made to the original paper authors. We have included the processed data used to create our figures in our Source Data.
Raw Illumina sequencing data is available in NCBI SRA database with Project ID PRJNA1392034 (http://www.ncbi.nlm.nih.gov/bioproject/1392034). Raw image files for tissue and skin imaging are available on request due to size but are also provided in our Source Data.

Research involving human participants, their data, or biological material

Policy information about studies with human participants or human data. See also policy information about sex, gender (identity/presentation), and sexual orientation and race, ethnicity and racism.

Reporting on sex and gender	No human participants or data or biological materials were involved in this study.
Reporting on race, ethnicity, or other socially relevant groupings	No human participants or data or biological materials were involved in this study.
Population characteristics	No human participants or data or biological materials were involved in this study.
Recruitment	No human participants or data or biological materials were involved in this study.
Ethics oversight	No human participants or data or biological materials were involved in this study.

Note that full information on the approval of the study protocol must also be provided in the manuscript.

Field-specific reporting

Please select the one below that is the best fit for your research. If you are not sure, read the appropriate sections before making your selection.

☒ Life sciences ☐ Behavioural & social sciences ☐ Ecological, evolutionary & environmental sciences

For a reference copy of the document with all sections, see nature.com/documents/nr-reporting-summary-flat.pdf

Life sciences study design

All studies must disclose on these points even when the disclosure is negative.

Sample size	No formal sample size calculation was performed for the metrics under analysis, as these metrics were designed post-data collection and as such it was not possible to estimate an anticipated effect size or standard deviation in advance. As our analysis involved assessing mice as individual ecosystems, our n number is sufficient to identify the heterogeneity present between individuals, and was adequate to assess significant differences of approximately 2-4x standard deviation with a power of 0.8 within the groups, for both re-infection vs single infection comparison (n=6 and n=5) and for early vs chronic infection comparison (n=4 and n=8), as per the NC3Rs experimental design assistant power calculator.
Data exclusions	One mouse infected with barcoded parasites for 4 weeks was excluded due to a lack of evidence of established infection at point of death. No further processing was performed on this mouse. One mouse infected with tdTomato-expressing parasites was excluded due to a welfare concern arising at week 7 of infection, leading to early euthanasia of this mouse prior to the anticipated 12 week time-point. This welfare concern was unrelated to infection or other experimental procedures performed to our knowledge. One barcode was excluded from all analysis for reasons discussed in the Methods section of the manuscript.
Replication	We ran all animal experiments with a minimum of three biological replicates (mice) per group. However, we note here that a key conclusion in our paper lay in the heterogeneity observed between individuals in terms of dissemination patterns. We observed interrelated parasite populations in different tissues and repeated skin-viscera, skin-skin and viscera-skin dissemination across individual mice, as indicated in Supplementary Data 1, 2 and 4. We also found that no re-infected mice showed evidence of within skin seeding above threshold (Supplementary Data 5), supporting the reproducibility of the finding that re-infection perturbs networks of dissemination in relation to skin. We did not repeat the full experimental set up for this using a separate infection inoculum as datasets with different initial inoculums used for

infection cannot be combined for statistical analysis, due to an inability to calibrate the FP calculations for our infection system which uses amastigote-stage parasites.
Experiments using tdTomato-expressing parasites for confocal imaging of skin also used at least three biological replicates per group (three or more mice per time-point).
Our in vitro infections were repeated with five parasite lines, and our in vitro growth barcode frequency change assessment was repeated three times.
Further, our simulations relied on large numbers of runs to check findings were reproducible and our validation work used at least three repeats for all analyses where this was possible.

Randomization

We randomly assigned mice to groups in the reinfection vs single infection study, by splitting the mice receiving treatments across cages (so that mice within one cage were not all receiving the same treatment) and then picking mice to be given each treatment at random within the cages.
For the tdTomato infection study, we selected the mice to be killed at each time-point at random, spread across the three cages infected with this parasite line.
For the early vs chronic infection experiment, mice were not randomized in terms of allocation; we used a single cage for the early infection group killed at 4 weeks post-infection.

Blinding

Blinding was not performed as experimental design, sample collection and analysis was performed by CL with assistance for sample collection from SD and HA.

Reporting for specific materials, systems and methods

We require information from authors about some types of materials, experimental systems and methods used in many studies. Here, indicate whether each material, system or method listed is relevant to your study. If you are not sure if a list item applies to your research, read the appropriate section before selecting a response.

Materials & experimental systems

n/a	Involved in the study	
☒	☐	Antibodies
☐	☒	Eukaryotic cell lines
☒	☐	Palaeontology and archaeology
☐	☒	Animals and other organisms
☒	☐	Clinical data
☒	☐	Dual use research of concern
☒	☐	Plants

Methods

n/a	Involved in the study	
☒	☐	ChIP-seq
☒	☐	Flow cytometry
☒	☐	MRI-based neuroimaging

Eukaryotic cell lines

Policy information about cell lines and Sex and Gender in Research

Cell line source(s)

Leishmania donovani LV9, luciferase-expressing L. donovani and tdTomato-expressing L. donovani.
Mouse bone marrow derived macrophages were obtained from bone marrow isolated from B6.CD45.1 mice.

Authentication

L. donovani LV9 was not validated; full genome sequence data for this line has previously been published.
L. donovani LV9 expressing T7 and cas9 was fully genome sequenced by us after generation. The barcoded parasite lines in the study were validated by PCR as detailed in Results and Figure 1.
Luciferase-expressing L. donovani was validated in the publication: ???
tdTomato-expressing L. donovani was validated in the publication: Beattie, L. et al. Dynamic imaging of experimental Leishmania donovani-induced hepatic granulomas detects Kupffer cell-restricted antigen presentation to antigen-specific CD8 T cells. PLoS Pathog. 6, e1000805 (2010).
Mouse macrophages were not validated after isolation from mice.

Mycoplasma contamination

Cell lines were not tested for Mycoplasma.

Commonly misidentified lines
(See ICLAC register)

None used in this study.

Animals and other research organisms

Policy information about studies involving animals; ARRIVE guidelines recommended for reporting animal research, and Sex and Gender in Research

Laboratory animals

C57BL/6J mice aged between 9-12 weeks (barcode infections) and 15-17 weeks (tdTomato infections) at the start of experimental studies. Mice were bred in-house at University of York.

Wild animals

No wild animals were used in this study.

Reporting on sex	Female mice were used in this study. Males were not included due to problems with aggression which result in chronic skin damage and inflammation, which would confound the results of our analysis.
Field-collected samples	No field-collected samples were used in this study.
Ethics oversight	All animal experiments were approved by the University of York Animal Welfare and Ethical Review Body (AWERB). Experiments were run in accordance with the Animals (Scientific Procedures) Act, 1986 and were performed under the Home Office Project Licence #PP0326977 ("Immunobiology of leishmaniasis").

Note that full information on the approval of the study protocol must also be provided in the manuscript.

Plants

Seed stocks	No plants were used in this study.
Novel plant genotypes	No plants were used in this study.
Authentication	No plants were used in this study.
