# Supplementary material for: Bi-directional highways and super-seeder tissues underpin parasite dissemination in experimental Leishmania donovani infection
Source: Nat Commun. 2026 Aug 26;17:10194. doi: 10.1038/s41467-026-77072-4 (PMC13612394; doi:10.1038/s41467-026-77072-4)
Supplement: Supplementary file 3 [file 41467_2026_77072_MOESM5_ESM.pdf]

nature portfolio

Peer Review File

Bi-directional highways and super-seeder tissues underpin parasite dissemination in experimental Leishmania donovani infection.

Corresponding Author: Professor Paul Kaye

This file contains all reviewer reports in order by version, followed by all author rebuttals in order by version.

Version 0:

Reviewer comments:

Reviewer #1

(Remarks to the Author)
This manuscript presents an ambitious and conceptually innovative study combining CRISPR-based barcoding of Leishmania donovani with ecological diversity metrics and network-based inference to investigate within-host dissemination in a murine model of visceral leishmaniasis. The integration of barcoding with ecological network analysis represents a substantial methodlogical advance and generates intriguing insights into tissue connectivity, bi-directional dissemination, and the identification of putative “super-seeder” sites.

The dataset is rich and the analytical framework is creative. However, some aspects of robustness, quantitative definition, and mechanistic interpretation require clarification and strengthening before the central conclusions can be considerd fully supported.

1. Robustness and uncertainty of directional inference

The key innovation of the manuscript is the derivation of TSFP and FFP metrics to infer directionality of parasite movement, followed by construction of directed networks and minimum spanning trees. While the validatoin analyses provided are appreciated, the manuscript would benefit from a more explicit quantification of uncertainty.

Specifically, I encourage the authors to:
a) Implement bootstrap resampling of barcode frequencies within tissue pairs to quantify confidence in inferred directionality (A to B vs B to A), particularly for sites designated as “super-seeders.”
b) Perform downsampling analyses demonstrating that TSFP/FFP magnitude and direction remain stable acros a range of sequencing depths.
c) Conduct sensitivity analyses showing that the principal conclusions (bi-directionality, identification of super-seeder sites, skin to viscera dissemination) are robust to alternative FFP thresholds and modest variation in read-depth or genetic distance cutoffs.

At present, network edges appear binary after thresholdng. Providing uncertainty estimates would materially strengthen confidence in the inferred dissemination structure.

2. Effective barcode diversity and library sufficiency

Although the use of 102 unique barcodes is pretty well justified via simulation, directional inference depends on the effective number and evenness of barcodes present in each tissue sample rather than the nominal library size alone.

It would strengthen the manuscript to report:
a) The effective barcode number per tissue (e.g., richness and/or Shannon-derived effective diverity).
(b) The number of barcodes typically contributing to TSFP calculations after removal of zero-frequency barcodes in pairwise comparisons.

This would allow readers to better assess the stability and precision of the inferred founder population estimates.

3. Definition and interpretation of “super-seeder” sites

The identification of “super-seeder” sites is an interesting and potentially important network-level observation. However, the operational definition of this phenotype is not fully clear. It would be helpful to clarify whether super-seeder status is defind by total outgoing TSFP, out-degree, eigen centrality, or a composite metric.

Additionally, the manuscript suggests that super-seeder sites may contain more actively dividing and diverse parasite populations. While biologically plausible, replication rate and barcode diversity are not necessarily alignd. High barcode diversity could arise from repeated immigration, reduced bottlenecking, or stochastic asymmetry rather than enhanced local proliferation.

I encourage the authors to more clearly distinguish between network-derived hub status and demonstrated biological mechanism, and to discuss alternative drivers of hub behaviour such as ongoing immigration, host-cell trafficking dynamics, local inflammatory tone, or temporal effects. Even withut additional experiments, a more nuanced framing would enhance the conceptual clarity.

4. Evidence for bi-directional dissemination

The conclusion of bi-directional dissemination between skin and visceral organs is central to the manuscript. Given that directionality is inferred from barcode structure rather than directly observed migration events, slightly more cautious phrasing would be appropriate.

The manuscript would benefit from quantification of how consistently skin to viscera signals are observed across animals, as well as additional sensitivity analyses to further rule out shared ancestry or background similarity as contributors to apparnt directionality.

5. Secondary infection experiments

The re-infection experiments suggest rerrouting of dissemination pathways and increased skin to viscera trafficking. This is intriguing but currently mechanistically a bit speculative.

It would be helpful to clarify whether measurable immunological changes accompany this rerouting and to confirm that differences in biopsy number or sampling depth do not contribute to the observed effects. Senstivity analyses similar to those suggested above would further strengthen this section.

6. Model limitations and translational framing

All experimental data derive from an intravenous murine infection model. While the discussion appropriately considers broader implications, conclusions regarding relapse and transmission should remain clearly framed as model-based inferences. A brief strengthening of this framing would enhance precision without diminishig the impact of the paper

Minor comments
- Consider replacing the term “dissemination highways” in the abstract with a more precise term such as “dominant dissemination routes,” even though the use of quotation marks mitigates concerns (but this a matter of taste)
- Provide justifiction for the genetic distance cutoff used in network construction.
-Consider adding a brief explanatory sentence linking ecological diversity metrics to biological interpretation for readers less familiar with ecological frameworks. This would really be helpful.

Overall, this is an innovative and potentially impactful study.

(Remarks on code availability)

Reviewer #2

(Remarks to the Author)
Review of “Bi-directional highways and super-seeder tissues underpin parasite dissemination in experimental visceral leishmaniasis”
This manuscript by Loughrey et al uses elegant technologies including ecological modeling and CRISPR barcoded

parasites to evaluate parasite dissemination after IV infection using a female BL6 model. Findings presented in this manuscript are interesting, but often presented in one-off ways, as there were not consistent patterns or pathways found during extensive analyses.
Despite very interesting, well-presented findings, the largest concern of this work is the generalizability to actual VL, as BL6 mice clear infection and do not develop any signs of disease. The group cited Sacks and Melby (1998) to say that this is a frequently used animal model, but Melby himself would be the first to indicate that to evaluate visceral dissemination and disease during VL, hamsters would be the best model system, perhaps followed by Balbc mice, and only as a last resort BL6 mice. As mentioned, BL6 male mice are more aggressive and can cause cutaneous damage that would change outcomes in these studies. But across all species it is males that are more susceptible to infection, doubly making the model choice hard to understand. This model choice required very high IV doses (between 3-8 107) of L. donovani parasites to ensure patent infection. This is of course very different than 100-1000 parasites found in most infectious sand flies during natural settings. IV infection of this level is the equivalent of a “fire hose” approach to infection. In this setting, where they then want to watch the “highways” and parasite dynamics between host viscera and the skin. It is very hard to know whether, based on this approach, any of the evidence presented would have real world relevance to L. donovani infection by a sand fly in the wild. This model and system could be more like blood donor infections, which are rare with universal testing mandated in most countries, or vertical transmission from a very infected mother, although that would then involve the extra complication of the neonatal immune system maturing during this initial infection, which may not be consistent with live birth. To address this, the manuscript should be heavily edited to provide context where high level IV infection would be the focus, which of course alters the impact of the findings to a much narrower context.
With this difference, the title should be “experimental high dose L. donovani infection” not experimental VL, as the BL6 model does not represent disease at all.
Also, in the setting of this model and high dose infection, all of the re-infection “model” is doubtful to model anything except high dose infection in a BL6 mouse and does not hold much interest to the disease or even natural infection with a less resistant genetic background. This should be removed to supplemental data with this context.

Minor considerations:
Figure 1 model should be explained better to indicate that the layers of pink and yellow next to the mice are skin (?) and then liver, gut and spleen should be labeled too to make sure others understand what this model depicts.
Throughout the paper there should be some indication or pinning to parasite burden in each sample so that diversity is in context of population size. High diversity could be only due to a large number of parasites, particularly as any data below 50,000 was removed from the model but is certainly a biologically important number of parasites for any actual patient.
For the skin samples it is indicated that there are three timepoints, d2, 4w and 12w. When “early” is discussed is this 2d or 4w? This should be made clear.
When the different skin samples are discussed, it would be great to show a map of where these were on the mice to see if certain areas seeded to particular organs.
Through the results section particular mice are used as examples but this is stochastic through the manuscript. It would be best to pick 2 different mice to use as consistent examples throughout to give a clear through line of a few stories of what is happening.
Starting in figure 4, TSFP is used often, and with some searching it can be identified that FP is founder population, but it would be nice to define this again at the start of this section of the results.
In the TSFP calculations, how was the larger n of skin samples accounted for to not overweigh these results towards skin seeding? What do the numbers in the TSFP y axes mean? Some provision for how these relate to one another (or not at all- fun with math modeling) would be useful.
In the description of the model, you can remove the description of g as its set to 1 and does not appear.

When validating the model, would have been great to use some non-mouse datasets to confirm if any of this can hold in a non mouse model.

There are several pages of explanatory discussion in the methods about the model. This can be removed to the discussion or all together.

Description of the samples Barseq analysis somewhat makes (found on line 721-22) it sound like samples were batched for sequencing purposes, please clarify that its one each of every organ listed vs. two of LN and femur/tibia. As written this is not clear.
Pi is used frequently in the later parts of the model description but is not defined- post-infection?

Figure 3. Why is skin to BM not provided, when the “representative” mouse shows a very strong connection between these? Perhaps either chose another mouse if this “highway” was only important in this mouse, or provide the stats for this.

(Remarks on code availability)
Sorry, not a coder myself, just work closely with them.

Reviewer #3

(Remarks to the Author)
The study from Professor Paul Kaye and his colleagues has employed CRISPR genome editing to generate a barcoded L. donovani library to be used in a new strategy to investigate the tissue dissemination patterns of L. donovani in a murine model of VL. Their findings reveal a bi-directional communication link between the skin and visceral organs and identified a

high degree of inter-connectivity among parasite populations. These findings elucidate the understanding of L. donovani pathogenesis, offering essential insights into the dynamics of infection with direct consequences for disease recurrence and transmission. The study represents an advancement that maps the current frontier of research in the field, presenting robust and relevant results to elucidate the understanding of L. donovani pathogenesis thus offering essential insights into the dynamics of infection with direct consequences for disease recurrence and transmission.

However, the authors should use this network-based analytical approach to infer about the tissue-specific effects on parasite fitness. L. donovani disseminates systemically and the approach demonstrated in the study proves useful for tracking the parasite infection routes and tissue dissemination. The results raise the possibility that parasites require particular adaptations to infect and survive in different tissues, indicating possible tissue-specific effects on parasite fitness during infection and re-exposure of the host. The authors should compare the fitness scores between anatomical sites in search of tissue-specific phenotypes. Fitness scores can be correlated between tissues so this would indicate how the parasites experience selective pressures in these environments during the infection period captured by their model. This issue is consistent with the strong bottlenecks experienced by the parasite population in these tissues, as demonstrated in their findings. In this line, analysis pipeline could be used to calculate differential fitness scores between tissues. This could be analysed from parasites harvested from different mouse tissues by addressing either a parasite transcriptome or RT-PCR analysis for altering gene expression candidate genes that could influence parasite-intrinsic processes such as metabolic adaptation, invasion, or immune evasion genes that could impact the infection fitness in mice thus contributing to host modulation of and immune evasion. This contribution would point to subsequent network-based analytical studies using comparative analysis with wildtype and mutant parasites for selected candidate genes inferred in this work model.

(Remarks on code availability)

Version 1:

Reviewer comments:

Reviewer #1

(Remarks to the Author)
The revised manuscript has been substantially strengthened. In particular, I appreciaite the considerable additional work performed to address the robustness of the directional inference framework, includng the jackknife analyses, barcode removal experiments, read-depth downsampling, and threshold sensitivity analyses. These additions significantly improve confidence in the analytical pipeline and demonstrate that the principl conclusions are not dependent on a single parameter choice or sequencing depth.

I also appreciate the clearer operational definition of “super-seeder” sites, the more cautious framing regarding inferred dissemination versus directly observed migrtion, and the improved discussion of alternative biological explanations underlying hub behavior. The authors have also appropriately strengthened the discussion of the limitations of the intravenous murine infection model and have revised the title and broader translational framing accordingly.

Importantly, the authors responded constructively to concerns regarding the reinfection experiments and transparently removed conclusions that were not robust following subsampling analysis. This substantially increases confidence inthe overall rigor of the study.

While the inherent limitations of the experimental model remain and direct mechanistic explanations for “super-seeder” behaviour are still speculative, I now feel that these limitations are appropriately acknowledged and no longer detract from the conceptual and methodological advance represented by the study.

Overall, I believe the manuscript has been significantly improved and is now suitable for publication in Nature Communications.

(Remarks on code availability)

Reviewer #3

(Remarks to the Author)
The work entitled "Bi-directional highways and super-seeder tissues underpin parasite dissemination in experimental Leishmania donovani infection" has employed CRISPR genome editing to generate a neutral barcoded L. donovani library and combined this with a novel network-based analytical approach to examine the dissemination patterns of L. donovani in a murine model of VL. Using this approach, the authors have shown a high degree of inter-connectivity between parasite populations demonstrating. The revised study has a clear cut demonstration of this evidence so that this manuscript now contributes to our understanding of L. donovani pathogenesis, offering a potential frontier for new insights that can be integrated into strategies for preventing relapse and controlling the spread of Leishmania and emerging drug resistance.

(Remarks on code availability)
The authors have adequately responded to the referees and the manuscript is now suitable for publication.

Open Access This Peer Review File is licensed under a Creative Commons Attribution 4.0 International License, which permits use, sharing, adaptation, distribution and reproduction in any medium or format, as long as you give appropriate credit to the original author(s) and the source, provide a link to the Creative Commons license, and indicate if changes were made.
In cases where reviewers are anonymous, credit should be given to 'Anonymous Referee' and the source.
The images or other third party material in this Peer Review File are included in the article's Creative Commons license, unless indicated otherwise in a credit line to the material. If material is not included in the article's Creative Commons license and your intended use is not permitted by statutory regulation or exceeds the permitted use, you will need to obtain permission directly from the copyright holder.
To view a copy of this license, visit https://creativecommons.org/licenses/by/4.0/

Response to reviewers

Reviewer comments italicized, author response in plain text, bold text in Author response indicates new data / text.

Reviewer #1

This manuscript presents an ambitious and conceptually innovative study combining CRISPR-based barcoding of Leishmania donovani with ecological diversity metrics and network-based inference to investigate within-host dissemination in a murine model of visceral leishmaniasis. The integration of barcoding with ecological network analysis represents a substantial methodlogical advance and generates intriguing insights into tissue connectivity, bi-directional dissemination, and the identification of putative "super-seeder" sites. The dataset is rich and the analytical framework is creative. However, some aspects of robustness, quantitative definition, and mechanistic interpretation require clarification and strengthening before the central conclusions can be considered fully supported.

We thank the reviewer for the kind comments and appreciation of methodological advance, the creativity of the analytical framework and the richness of the data. We have addressed the specific points below:

1. Robustness and uncertainty of directional inference: The key innovation of the manuscript is the derivation of TSFP and FFP metrics to infer directionality of parasite movement, followed by construction of directed networks and minimum spanning trees. While the validation analyses provided are appreciated, the manuscript would benefit from a more explicit quantification of uncertainty.

We thank the reviewer for these very helpful suggestions for further validation of the uncertainty in our approach.

Specifically, I encourage the authors to:
a) Implement bootstrap resampling of barcode frequencies within tissue pairs to quantify confidence in inferred directionality (A to B vs B to A), particularly for sites designated as "super-seeders."

Rather than bootstrapping, we have validated uncertainty using a jack-knife approach of iterative removal of each barcode. We elected to use this approach for a combination of reasons. Firstly, it is frequently used for the same purposes of uncertainty quantification as bootstrapping, but is much less computationally expensive, whilst published work indicates it may be a less biased and more appropriate method with which to quantify uncertainty from smaller sample sizes[1–3] (such as our ~100 barcode library). Additionally, we think it is more appropriate to quantify the uncertainty of the barcodes individually across the dataset by iterative removal, rather than by random re-sampling with replacement, which would add variation that will inherently alter the FP estimation by definition, as this metric is reliant

upon variance in individual barcode counts between input and output, making the output from bootstrapping difficult to evaluate.

We applied this approach and compared the direction and magnitude of FFP with that calculated from the full set of barcodes to assess how certain we can be of our estimates of FFP. We have included this analysis in the Validation section of Methods (**lines 704-717**), and in **Supplementary Table 23** (direction matching), **Supplementary Note Part 5** (magnitude correlation) and the new **Supplementary Figure 5** (magnitude). We have further applied this logic by removing randomly selected proportions of barcodes (amounting to 5%, 10%, 25% and 50% of the total barcodes) and running the analysis pipeline through, with 20 repeats of this for each percentage tested, to assess how many sample pairs have an FFP which conserves the overall direction of migration inferred. This analysis is included in Methods (**lines 704-717**), **Supplementary Table 24** (direction matching) and **Supplementary Note Part 5** (magnitude correlation).

b) Perform downsampling analyses demonstrating that TSFP/FFP magnitude and direction remain stable acros a range of sequencing depths.

We have performed a down-sampling approach to assess how read depth affects the accuracy of TSFP estimation (and thus the FFP estimates which rely on TSFP). It should be noted that removal of reads will inherently alter the magnitude of TSFP compared to TSFP calculated with more reads, as the input pool is thus a different size and therefore the relative founder sizes will differ. However, the consistency of TSFP with reduced read depths is important to understand, given the possible technical variation and the subjective nature of sample read depth choices in these experiments. To do this, we removed raw fastq reads at random prior to running the barcode extraction and subsequent analysis in our pipeline and repeated this approach 5 times each. We removed 10% of reads initially, then repeated the approach with removal of 25% and 50% of reads, before quantifying the correlation of the TSFP values obtained with those calculated using all the fastq reads. In all three cases we noted a very high correlation (96%, 93% and 87% respectively) with the "100% reads" TSFP. We have included this analysis in our Methods section (**lines 718-735**), with **Supplementary Table 25**, **Supplementary Note Part 6** and **Supplementary Figure 6** detailing our findings.

c) Conduct sensitivity analyses showing that the principal conclusions (bi-directionality, identification of super-seeder sites, skin to viscera dissemination) are robust to alternative FFP thresholds and modest variation in read-depth or genetic distance cutoffs.
At present, network edges appear binary after thresholding. Providing uncertainty estimates would materially strengthen confidence in the inferred dissemination structure.

The FFP threshold was applied only in the context of drawing conclusions from the network analysis. Therefore, all conclusions based on the TSFP statistical comparisons between groups are unaffected by threshold choice. We have rerun the FFP calculation pipeline and subsequent statistical analysis of centrality metrics (Eigen Centrality, In-degree and Out-degree) and have added these as **Supplementary Tables 22a, b and c**. This analysis finds

that our overall conclusions are still supported when applying both a reduced and increased threshold to our analysis. Our Genetic Distance cut off of 0.5 was applied solely to the visualisation of the networks and not to our analysis. We realise this was not made clear in our Methods section and have now altered this to clarify for readers (**lines 627-628**). Please see the above point for the analysis of variation in read depth. With reference to the comment about binary FFP, we included scales showing the value of FFP in our networks in our original **Extended Data Figure 6**. However, these were originally very small – we have now increased their size to ensure the magnitudes of FFPs are clear to readers.

2. Effective barcode diversity and library sufficiency: Although the use of 102 unique barcodes is pretty well justified via simulation, directional inference depends on the effective number and evenness of barcodes present in each tissue sample rather than the nominal library size alone. It would strengthen the manuscript to report:
a) The effective barcode number per tissue (e.g., richness and/or Shannon-derived effective diversity). (b) The number of barcodes typically contributing to TSFP calculations after removal of zero-frequency barcodes in pairwise comparisons.
This would allow readers to better assess the stability and precision of the inferred founder population estimates.

Thank you for this suggestion to allow better assessment of our metric estimates. We included Shannon Index values and the code for this analysis with the original manuscript (on Github and within Figure 2), but we have now added **Supplementary Table 16** to our Supplementary files, containing all Shannon Indices for each sample. We have also included as suggested the number of barcodes contributing to TSFP calculations as **Supplementary Table 18** (which is equivalent to the effective barcode "richness" suggested in part a). We have also added an in-text reference within our Methods section (**line 610**).

3. Definition and interpretation of "super-seeder" sites: The identification of "super-seeder" sites is an interesting and potentially important network-level observation. However, the operational definition of this phenotype is not fully clear. It would be helpful to clarify whether super-seeder status is defined by total outgoing TSFP, out-degree, eigen centrality, or a composite metric.

Thank you for pointing out that our definition of super-seeders was not clear. We have now edited the text in Results (**lines 207-208**) to state that we have defined our "super-seeder" sites as those which seed multiple other sites (i.e. those which have a high out-degree). The other metrics, including eigen centrality and total outgoing TSFP, were used to reinforce this concept of "super-seeders" by providing additional supporting evidence of high levels of seeding, but the designation of a "super-seeder" was based on the number of (rather than magnitude of) outward seeding events.

Additionally, the manuscript suggests that super-seeder sites may contain more actively dividing and diverse parasite populations. While biologically plausible, replication rate and barcode diversity are not necessarily aligned. High barcode diversity could arise from repeated immigration, reduced bottlenecking, or stochastic asymmetry rather than enhanced local proliferation.

In the context of the skin, our reasoning for suggesting that local proliferation may be linked to "super-seeding" was based on our confocal imaging, in which we see heterogeneous tdTomato signal across skin patches; we hypothesise that this may be related to differences in parasite replication rates, given previously published data on parasite quiescence in the skin in the context of CL (as also determined by tdTomato signal). We agree that it is possible that there are differences in parasite numbers across patches due to migration, or varying signal may be due to differences in parasite metabolic activity, rather than being a result of heterogeneous replication rates across the skin. Likewise, differences in parasite death, due to heterogeneity in the local immune response, is also a plausible mechanism for these observations. We have therefore changed our wording to reflect these possibilities in the Discussion (**line 349-356**).

I encourage the authors to more clearly distinguish between network-derived hub status and demonstrated biological mechanism, and to discuss alternative drivers of hub behaviour such as ongoing immigration, host-cell trafficking dynamics, local inflammatory tone, or temporal effects. Even without additional experiments, a more nuanced framing would enhance the conceptual clarity.

We have noted in our data that sites which act as "super-seeders" are not necessarily those which receive high immigration from other locations – indeed most do not show this pattern. Therefore, we are inclined to think that high levels of continued inward migration are not linked to super-seeder status. Earlier stage migration which has since ceased and/or restrictive early bottlenecks to initial colonisation could contribute to increased diversity and parasite density, and we have added text to this effect to our Discussion (**lines 356-359**).

4. Evidence for bi-directional dissemination: The conclusion of bi-directional dissemination between skin and visceral organs is central to the manuscript. Given that directionality is inferred from barcode structure rather than directly observed migration events, slightly more cautious phrasing would be appropriate.

We agree and have reworded our Discussion and Results sections to be more cautious regarding the inference of migration and have also added into our Discussion and Results a clear statement including this caveat (**lines 203-204 and 418-423**).

The manuscript would benefit from quantification of how consistently skin to viscera signals are observed across animals, as well as additional sensitivity analyses to further rule out shared ancestry or background similarity as contributors to apparent directionality.

We have reworded our Discussion and Results sections to be more cautious regarding the inference of migration and have also added into our Discussion and Results a clear statement including this caveat (see above point). We have added **Supplementary Tables 4a-d** which summarise the numbers of dissemination events of different types observed across mice. We have also altered our discussion to add the caveat that our study uses only one strain of in-bred mice with the same genetic background (**lines 408-411**).

5. Secondary infection experiments: The re-infection experiments suggest rerouting of dissemination pathways and increased skin to viscera trafficking. This is intriguing but currently mechanistically a bit speculative. It would be helpful to clarify whether measurable immunological changes accompany this rerouting and to confirm that differences in biopsy number or sampling depth do not contribute to the observed effects. Senstivity analyses similar to those suggested above would further strengthen this section.

Thank you for this suggestion to check that biopsy number variance was not affecting the conclusions we drew regarding the re-routing of dissemination after re-infection. We have run a sub-sampling analysis using matched biopsy numbers (with random selection of biopsies from the 12 taken from single-infection mice, and 5 repeat runs), which leads us to confirm that the conclusions of increased skin-viscera and reduced skin-skin trafficking are not a result of differences in sampling depth. We have included new **Supplementary Tables 13b and 14b** which show this sub-sampling. However, after reading this comment, we realised it was likely that the additional comparisons we ran of "total TSFP" differences between single and re-infected skin were likely spurious, given that the overall area sampled was higher for single-infection mice. We ran the same sub-sampling approach and confirmed this to be the case, finding no significance when a smaller pool of biopsies was used. Therefore, we have removed this from the manuscript.

We did not measure any immunological parameters for this study, so unfortunately we do not have data on whether specific immunological changes accompanied our re-routing observations. However, we agree that this would be a valuable insight and would assist in identifying mechanisms underlying these effects, and we have added wording to this effect to the Discussion (**lines 400-403**).

6. Model limitations and translational framing: All experimental data derive from an intravenous murine infection model. While the discussion appropriately considers broader implications, conclusions regarding relapse and transmission should remain clearly framed

as model-based inferences. A brief strengthening of this framing would enhance precision without diminishing the impact of the paper

Thank you for suggesting this and for noting that the impact of the study is through its conceptual advance and not reliant on the experimental model precisely recapitulating human disease. We have edited the Discussion to make clear that all inference of applicability to humans is based on this model system, and that further studies with more clinically-reflective models would be valuable (**lines 375-379 and 408-410**).

Minor comments
- Consider replacing the term "dissemination highways" in the abstract with a more precise term such as "dominant dissemination routes," even though the use of quotation marks mitigates concerns (but this a matter of taste)

As requested, we have modified the text to read "….dominant dissemination routes or "highways"…." (**line 34**) and also used this terminology in the Discussion and Results sections.

Provide justification for the genetic distance cutoff used in network construction.

As mentioned above, we applied this cut off only for the visualisation of the networks, as it makes it much easier to see the strong connections if we remove the weaker ones from the visualisations. It was not used for any of the statistical analysis which included all GD data points. We realise this was not made clear in our Methods section and have now edited this (**line 627-628**).

Consider adding a brief explanatory sentence linking ecological diversity metrics to biological interpretation for readers less familiar with ecological frameworks. This would really be helpful.

We thank the reviewer for this excellent suggestion and have added appropriate text and references to the Introduction (**lines 58-63**).

Overall, this is an innovative and potentially impactful study.

We thank the reviewer for this comment

Reviewer #2

This manuscript by Loughrey et al uses elegant technologies including ecological modeling and CRISPR barcoded parasites to evaluate parasite dissemination after IV infection using a

female BL6 model. Findings presented in this manuscript are interesting, but often presented in one-off ways, as there were not consistent patterns or pathways found during extensive analyses.

We thank the reviewer for the positive comments and discuss the inter-mouse variability below.

Despite very interesting, well-presented findings, the largest concern of this work is the generalizability to actual VL, as BL6 mice clear infection and do not develop any signs of disease. The group cited Sacks and Melby (1998) to say that this is a frequently used animal model, but Melby himself would be the first to indicate that to evaluate visceral dissemination and disease during VL, hamsters would be the best model system, perhaps followed by Balbc mice, and only as a last resort BL6 mice.

The reviewer is correct to say that B6 mice clear infection, but incorrect in saying that they do not develop any signs of disease. Multiple studies have shown comparable immunopathological changes in the viscera of mice, hamsters and dogs and humans. Where mice and hamsters differ is in the amplification of specific immunoregulatory pathways and in the fact that hamsters progress to fatal end stage disease (a feature in part attributed to intestinal barrier dysfunction and endotoxemia). Melby has used the murine model on numerous occasions for studies of dissemination (PMID: 36630476; 40360769) and a recent paper in NCOMMS by Sacks similarly uses B6 mice as the model of choice (PMID: 40169598). Both authors describe their work as modelling "visceral leishmaniasis".

As mentioned, BL6 male mice are more aggressive and can cause cutaneous damage that would change outcomes in these studies. But across all species it is males that are more susceptible to infection, doubly making the model choice hard to understand.

The reviewer is correct concerning the sex bias in human VL, but here we are exploring novel concepts in parasite dissemination that were previously unstudied. It is thus appropriate where possible to reduce confounding factors, such as spurious inflammation.

This model choice required very high IV doses (between 3-8 107) of L. donovani parasites to ensure patent infection. This is of course very different than 100-1000 parasites found in most infectious sand flies during natural settings. IV infection of this level is the equivalent of a "fire hose" approach to infection. In this setting, where they then want to watch the "highways" and parasite dynamics between host viscera and the skin. It is very hard to know whether, based on this approach, any of the evidence presented would have real world relevance to L. donovani infection by a sand fly in the wild. This model and system could be more like blood donor infections, which are rare with universal testing mandated in most countries, or vertical transmission from a very infected mother, although that would then involve the extra complication of the neonatal immune system maturing during this initial

infection, which may not be consistent with live birth. To address this, the manuscript should be heavily edited to provide context where high level IV infection would be the focus, which of course alters the impact of the findings to a much narrower context.

The reviewer makes some valid comparisons but fails to appreciate the value of model systems to uncover novel aspects of infection biology that cannot be obtained from the study of human disease. We have not attempted to model all aspects of *L. donovani* infection, and our choice of model and our interpretations of the data do not make any claims regarding dissemination from the initial sand fly bite, nor how dissemination patterns may alter in the face of progressive fatal disease. As stated in the text (lines 122, 452 and 453), this model is chosen by us and others to provide rapid and consistent systemic parasite colonisation and our data therefore addresses dissemination patterns from this defined starting point. The methodology and concepts we describe can be adapted in future studies to address other phases of the infection process, as raised by the reviewer.

With this difference, the title should be "experimental high dose L. donovani infection" not experimental VL, as the BL6 model does not represent disease at all.

As requested, we have modified the title to "…experimental *L. donovani* infection". The dose used for infection, and the reasons for this choice, are clearly articulated in the Methods (lines 448-453).

Also, in the setting of this model and high dose infection, all of the re-infection "model" is doubtful to model anything except high dose infection in a BL6 mouse and does not hold much interest to the disease or even natural infection with a less resistant genetic background. This should be removed to supplemental data with this context.

We disagree and again point to the value of the model to uncover new biology. What these experiments show is that patterns of dissemination are not fixed in stone and can be modified by additional inflammatory / immunological challenge (in the current case, re-infection). Rather than not holding "much interest" we might argue that this is one of the central messages of the paper, demonstrating the malleability of dissemination patterns in response to inflammatory / immunological insult.

Minor considerations:
Figure 1 model should be explained better to indicate that the layers of pink and yellow next to the mice are skin (?) and then liver, gut and spleen should be labeled too to make sure others understand what this model depicts.

Thank you for pointing out the lack of definition for this figure (which we assume refers to Figure 2a). We have now labelled the tissues as suggested.

Throughout the paper there should be some indication or pinning to parasite burden in each sample so that diversity is in context of population size. High diversity could be only due to a large number of parasites, particularly as any data below 50,000 was removed from the model but is certainly a biologically important number of parasites for any actual patient.

We appreciate this suggestion. Diversity and parasite number are not necessarily linked at the stages of infection we are measuring. Whilst a high number of parasites at initial stages would likely correlate to parasite diversity at that point in time, at later stages of infection this is not necessarily the case; parasites may expand from very small initial populations, or conversely may not replicate or may die at differing rates, and there is further complexity derived from migration events occurring after initial colonisation that may be of varying levels of diversity and frequency. These complex dynamics mean a very large population may not actually be very diverse, whilst a small population may contain more diversity overall.[4–6]

We would also like to clarify that the 50,000 reads is not 50,000 parasites. This is the read number for the barcodes sequenced, which have undergone rounds of PCR beforehand. Whilst we suspect read depth may be a limiting factor where parasite numbers are very low, this is not necessarily the case and samples could contain below 50,000 reads due to technical aspects of the protocol.

For the skin samples it is indicated that there are three timepoints, d2, 4w and 12w. When "early" is discussed is this 2d or 4w? This should be made clear.

We thank the reviewer for pointing this out. We have altered the text of both Results (**lines 142-144**) and Methods (**lines 444-445**) to clarify the time-points used. For reference, the time-points for the sequencing experiment were 4 weeks (early) and 10-12 weeks (chronic) post-infection, whilst for the confocal imaging experiment, they were 2 days, 4 weeks and 12 weeks post-infection. We apologise for any confusion caused.

When the different skin samples are discussed, it would be great to show a map of where these were on the mice to see if certain areas seeded to particular organs.

We randomly took biopsies from the ventral abdominal skin. We have clarified this in the Results section (**line 150**). Our previous work found no evidence that parasites accumulate in skin patches in any discernible anatomical pattern[7,8] but the question of whether dissemination patterns from skin to viscera might be affected by specific skin location could be addressed in future studies.

Through the results section particular mice are used as examples but this is stochastic through the manuscript. It would be best to pick 2 different mice to use as consistent examples throughout to give a clear through line of a few stories of what is happening.

We appreciate this suggestion and agree it would be a good approach if we were aiming to highlight the specific trajectories occurring within individuals. However, our paper narrative was focussed on highlighting the heterogeneous dynamics occurring across mice, with the specific mice we chose to include in the main figures as examples selected specifically for their ability to illustrate different dynamics we observed. We have therefore left the included exemplar mice as they are in the main figures. We have included the labelled networks and heatmaps for all mice as Extended Data Figures with the manuscript, so if readers wish to follow the story of a specific mouse, they are able to do so.

Starting in figure 4, TSFP is used often, and with some searching it can be identified that FP is founder population, but it would be nice to define this again at the start of this section of the results.

Many thanks for this helpful suggestion. We have now added a re-definition of FP to the first mention of TSFP (**lines 198-199**).

In the TSFP calculations, how was the larger n of skin samples accounted for to not overweigh these results towards skin seeding? What do the numbers in the TSFP y axes mean? Some provision for how these relate to one another (or not at all- fun with math modeling) would be useful.

Please see the response to reviewer 1 above for the assessment of skin seeding using differing biopsy numbers. TSFP is the metric shown on the Y-axis of relevant plots. It is a relative measure of the Founder Population which could feasibly be sourced from the input tissue of interest, using the same calculation procedure as the more standard quantification of Founder Population from inoculum in Abel et al., 2015. We cannot calibrate our metric in an absolute sense, due to the technical issues involved in *Leishmania* amastigote isolation. Our metric is necessarily a relative one in this case. If used in other systems, then calibration against known samples would be possible to incorporate.

In the description of the model, you can remove the description of g as its set to 1 and does not appear.

We could do this, but we have specifically elected not to, as this parameter can in principle be altered if the generation time is known or can be estimated for the system in question. Whilst we do not know this information and thus set this as 1 for our study, we want to enable other researchers to develop on our approach for their own studies, and altering g may be of use in their work. Additionally, we wanted it to be very clear where we have derived our approach from, and g is used in the original Abel et al., 2015 paper when describing their equation. Therefore, if the editor and reviewer agree, we prefer to leave this as g in the Methods.

When validating the model, would have been great to use some non-mouse datasets to confirm if any of this can hold in a non mouse model.

This is an interesting suggestion. However, the number of suitable datasets for such validation is limited, as few published studies use multiple tissue samples from the same animal for STAMP analysis. Most studies of pathogen population dynamics also only use mice. However, we will explore avenues to test the approach in other systems in the future.

There are several pages of explanatory discussion in the methods about the model. This can be removed to the discussion or all together.

We have retained this text in the Methods. We feel this validation section is important for readers to evaluate our approach effectively. Given the structure of articles published in Nature Communications, moving this text to the Discussion would unnecessarily unbalance the Discussion and detract from the major conclusions of the manuscript.

Description of the samples Barseq analysis somewhat makes (found on line 721-22) it sound like samples were batched for sequencing purposes, please clarify that its one each of every organ listed vs. two of LN and femur/tibia. As written this is not clear.

Thank you for pointing this out. We have now edited the Methods (**lines 814-817**) to clarify that samples were all processed individually (one of each organ, plus two individual LN, two femur/tibia and the relevant number of skin biopsies).

Pi is used frequently in the later parts of the model description but is not defined- post-infection?

Thank you for pointing out this omission. We have now defined "p.i" as post-infection in the first section of the Results (**line 116**).

Figure 3. Why is skin to BM not provided, when the "representative" mouse shows a very strong connection between these? Perhaps either chose another mouse if this "highway" was only important in this mouse, or provide the stats for this.

The data for the skin-BM connection is provided in the first panel of Figure 3f. We selected the two mice shown on the basis that they reflect differing dynamics with reference to the skin-skin and viscera-viscera connections, as we felt these mice illustrated two extremes nicely. However, all GD networks for every mouse are included in Extended Data Figure 3 so that each can be compared fully.

Reviewer #3

The study from Professor Paul Kaye and his colleagues has employed CRISPR genome editing to generate a barcoded L. donovani library to be used in a new strategy to investigate the tissue dissemination patterns of L. donovani in a murine model of VL. Their findings reveal a bi-directional communication link between the skin and visceral organs and identified a high degree of inter-connectivity among parasite populations. These findings elucidate the understanding of L. donovani pathogenesis, offering essential insights into the dynamics of infection with direct consequences for disease recurrence and transmission. The study represents an advancement that maps the current frontier of research in the field, presenting robust and relevant results to elucidate the understanding of L. donovani pathogenesis thus offering essential insights into the dynamics of infection with direct consequences for disease recurrence and transmission.

We thank the reviewer for the kind words and appreciation of the cutting-edge nature of the study.

However, the authors should use this network-based analytical approach to infer about the tissue-specific effects on parasite fitness. L. donovani disseminates systemically and the approach demonstrated in the study proves useful for tracking the parasite infection routes and tissue dissemination. The results raise the possibility that parasites require particular adaptations to infect and survive in different tissues, indicating possible tissue-specific effects on parasite fitness during infection and re-exposure of the host. The authors should compare the fitness scores between anatomical sites in search of tissue-specific phenotypes. Fitness scores can be correlated between tissues so this would indicate how the parasites experience selective pressures in these environments during the infection period captured by their model. This issue is consistent with the strong bottlenecks experienced by the parasite population in these tissues, as demonstrated in their findings. In this line, analysis pipeline could be used to calculate differential fitness scores between tissues

We thank the reviewer for this detailed and insightful feedback on our work. The suggestion of applying fitness scoring to understand differences in parasite adaptation to varied tissue environments is interesting. A key element of our experimental design is utilisation of neutral barcoding to eliminate any selective effects. Hence, we cannot apply a fitness score approach to our dataset here. This approach could, however, be utilised in follow-on studies, applying barcoding to mutant parasites, to understand the importance of different candidate genes for colonisation of different tissues or through SNP analysis based on whole genome sequencing of parasites from the different tissues. We have added mention of these approaches to Discussion (**lines 364-373**), but the extensive experimental work required is beyond the scope of the current manuscript which is already substantive and is focused on describing the modelling of dissemination and reporting the key findings regarding dissemination patterns

This could be analysed from parasites harvested from different mouse tissues by addressing either a parasite transcriptome or RT-PCR analysis for altering gene expression candidate genes that could influence parasite-intrinsic processes such as metabolic adaptation, invasion, or immune evasion genes that could impact the infection fitness in mice thus contributing to host modulation of and immune evasion. This contribution would point to subsequent network-based analytical studies using comparative analysis with wildtype and mutant parasites for selected candidate genes inferred in this work model.

We agree with the suggestion that a comparative parasite transcriptome analysis in different tissues may prove interesting, despite our previous work that identified only minimal differences in gene expression between amastigotes isolated from spleen and liver[9]. Given the prominence of post-transcriptional regulation of gene expression in *Leishmania*, it would, however, also be important to conduct proteomic analysis to gain a full picture of the processes and drivers involved. We have added text and references related to this suggestion to the Discussion (**lines 364-373**), but as above believe this work to also be beyond the scope of the current study.

References

1. Kopuchian, C. & Ramírez, M. J. Behaviour of resampling methods under different weighting schemes, measures and variable resampling strengths. *Cladistics* **26**, 86–97 (2010).
2. Giraldo, R. Bootstrap versus Jackknife: Confidence intervals, hypothesis testing, density estimation, and kernel regression. *Cienc. En Desarro.* **15**, (2024).
3. Efron, B. Nonparametric estimates of standard error: The jackknife, the bootstrap and other methods. *Biometrika* **68**, 589–599 (1981).
4. Hullahalli, K. & Waldor, M. K. Pathogen clonal expansion underlies multiorgan dissemination and organ-specific outcomes during murine systemic infection. *eLife* **10**, e70910 (2021).
5. Wincott, C. J. *et al.* Cellular barcoding of protozoan pathogens reveals the within-host population dynamics of Toxoplasma gondii host colonization. *Cell Rep. Methods* **2**, 100274 (2022).

6. Campbell, I. W., Hullahalli, K. & Waldor, M. K. Quantifying host-microbe interactions with bacterial lineage tracing. *Science* **391**, 34–40 (2026).
7. Doehl, J. S. P. *et al.* Spatial Point Pattern Analysis Identifies Mechanisms Shaping the Skin Parasite Landscape in Leishmania donovani Infection. *Front. Immunol.* **12**, 795554 (2021).
8. Doehl, J. S. P. *et al.* Skin parasite landscape determines host infectiousness in visceral leishmaniasis. *Nat. Commun.* **8**, 57 (2017).
9. Forrester, S. *et al.* Tissue Specific Dual RNA-Seq Defines Host-Parasite Interplay in Murine Visceral Leishmaniasis Caused by Leishmania donovani and Leishmania infantum. *Microbiol. Spectr.* **10**, e0067922 (2022).
