# Supplementary material for: Bi-directional highways and super-seeder tissues underpin parasite dissemination in experimental Leishmania donovani infection
Source: Nat Commun. 2026 Aug 26;17:10194. doi: 10.1038/s41467-026-77072-4 (PMC13612394; doi:10.1038/s41467-026-77072-4)
Supplement: Supplementary file 4 [file 41467_2026_77072_MOESM2_ESM.docx]

**Description of Additional Supplementary Files**

**Supplementary Data 1:** Correlation of Founder Population (FP) calculated using a range of simulated library sizes, against the true FP (defined as the count of individuals). The test used as a Kendall correlation test.

**Supplementary Data 2:** Correlation of Founder Population (FP) calculated using a range of simulated library sizes, against the true FP (defined as the count of individuals), up to FP of 5000. The test used was a Kendall correlation test.

**Supplementary Data 3:** Mann-Whitney test results for Shannon Index comparisons between early and chronic infection for all tissues

**Supplementary Data 4:** Mann-Whitney test results for Genetic Distance comparisons between early and chronic infection for all tissue pairs

**Supplementary Data 5:** Dissemination events for skin-skin migration across all mice

**Supplementary Data 6:** Dissemination events for skin-viscera migration across all mice

**Supplementary Data 7:** Dissemination events for viscera-viscera migration across all mice

**Supplementary Data 8:** Dissemination events for viscera-skin migration across all mice

**Supplementary Data 9:** Mann-Whitney test results for Out-Degree comparisons between early and chronic infection for all tissues

**Supplementary Data 10:** Mann-Whitney test results for In-Degree comparisons between early and chronic infection for all tissues

**Supplementary Data 11:** Mann-Whitney test results for total TSFP (viscera-skin seeding) between early and chronic infection for all tissues

**Supplementary Data 12:** Mann-Whitney test results for total TSFP (skin-viscera seeding) between early and chronic infection for all tissues

**Supplementary Data 13:** Mann-Whitney test results for individual TSFP (seeding) between early and chronic infection for all tissues

**Supplementary Data 14:** Mann-Whitney test results for total TSFP (skin-skin seeding) between early and chronic infection

**Supplementary Data 15:** Mann-Whitney test results for eigen centrality between early and chronic infection

**Supplementary Data 16:** Mann-Whitney test results for radiance (parasite burden) quantified using the IVIS, between tissues from single or re-infected mice

**Supplementary Data 17:** Mann-Whitney test results for individual TSFP (seeding) between single and re-infection for all tissues

**Supplementary Data 18:** Sub-sampled Mann-Whitney test results for individual TSFP (seeding) between single and re-infection for individual skin biopsies to all tissues

**Supplementary Data 19:** Mann-Whitney test results for individual TSFP (seeding) between single and re-infection for skin to grouped visceral tissues

**Supplementary Data 20:** Sub-sampled Mann-Whitney test results for individual TSFP (seeding) between single and re-infection for skin to grouped visceral tissues

**Supplementary Data 21:** All barcode insertion oligo sequences used in this study

**Supplementary Data 22:** All other oligo sequences used in this study

**Supplementary Data 23:** Sequence and barcode read counts for all samples, noting samples removed for TSFP calculation (those with below 50000 reads)

**Supplementary Data 24:** Total number of barcodes used for TSFP calculation for all samples

**Supplementary Data 25:** Percentage of FFP above thresholds for simulations vs real dataset

**Supplementary Data 26:** Proportion of FFP above thresholds for true migration

**Supplementary Data 27:** Proportion of FFP above threshold for false migration

**Supplementary Data 28:** Out-dgree MW results when applying varying thresholds for FFP

**Supplementary Data 29:** In-degree MW results when applying varying thresholds for FFP

**Supplementary Data 30:** Eigen centrality MW results when applying varying thresholds for FFP

**Supplementary Data 31:** Directionality matching for FFP when applying jack-knife removal of barcodes, compared to the full set of barcodes

**Supplementary Data 32:** Magnitude correlation for FFP when sub-sampling the barcode pool, compared to the full set of barcodes

**Supplementary Data 33:** TSFP correlation when calculated with a reduced sequence read depth, against the full set of sequence reads
